# A Dual‐Responsive Versatile Nanohybrid Orchestrating Tumor Elimination and Tumor‐Associated Osteolysis Restoration via Sequential Release

**DOI:** 10.1002/advs.202518962

**Published:** 2026-03-18

**Authors:** Lan Liu, Han‐Zhe Liu, Zhe‐Nan Liu, Tong Wang, Li‐Li Yu, Qiu‐Jing Li, Zi‐Yi Chen, Guo‐Feng Luo, Zheng‐Jun Shang

**Affiliations:** ^1^ State Key Laboratory of Oral & Maxillofacial Reconstruction and Regeneration Hubei Key Laboratory of Stomatology Key Laboratory of Oral Biomedicine Ministry of Education School & Hospital of Stomatology Wuhan University Wuhan P. R. China; ^2^ Taikang Center for Life and Medical Sciences of Wuhan University Wuhan P. R. China

**Keywords:** bone regeneration, ion‐driven synergistic therapy, sequential release, tumor‐associated bone invasion, versatile nanohybrid

## Abstract

Tumor‐associated bone invasion often occurs in aggressive tumors and strongly compromises the efficacy of tumor treatment, which makes it challenging for comprehensive tumor therapy, highlighting the importance of simultaneous tumor elimination and tumor‐associated osteolysis restoration. To achieve this goal, this study reports a versatile nanohybrid (CaO_2_@CuMOF@HAP) synthesized by coating bimetallic nanoclusters (CaO_2_@CuMOF) with osteogenic growth peptide (OGP)‐modified hyaluronic acid (HAP) for high‐performance oncotherapy. On‐demand sequential release is realized in specific tumor environments, where OGP can be released into matrix metalloproteinase‐9 (MMP9)‐enriched tumor extracellular space through cleavage of the MMP9‐responsive linker between OGP and HA, and dual ions (Cu^2+^ and Ca^2+^) are liberated via pH‐triggered decomposition of the nanohybrid following tumor cell internalization. On the basis of this, small‐sized OGP could penetrate into deep‐seated, tumor‐involved, bone areas for promoting effective osteogenesis, while the excessive ions in targeted tumor cells synergistically disrupted intracellular ion homeostasis for effective metal ion interference therapy. Having killed tumor cells and promoted osteogenesis, CaO_2_@CuMOF@HAP exhibited highly efficient antitumor effects on an orthotopic oral squamous cell carcinoma tumor‐bearing mouse model with mandibular bone invasion. Without cytotoxic drugs, this nanohybrid circumvents drug resistance and nonspecific toxicity, offering excellent biocompatibility and high antitumor efficiency for clinical application.

## Graphical abstract

The versatile CaO_2_@CuMOF@HAP nanohybrid demonstrates an outstanding MMP9/pH‐triggered release performance for sequential release of osteogenic growth peptide into deep‐seated tumor‐infiltrated bone destruction areas and dual ions into tumor cells, enabling efficient tumor‐associated osteolysis restoration and tumor elimination for more comprehensive cancer therapy.

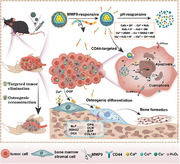

## 1 Introduction

Tumor‐associated bone invasion, characterized by destructive skeletal‐related events (e.g., hypercalcemia, spinal cord compression, and pathologic fractures), represents a prevalent complication in solid malignancies, which results in reduced patient overall survival and quality of life [1, 2, 3]. Oral squamous cell carcinoma (OSCC) is a typical example which frequently invades jawbones due to its anatomical proximity and osteolytic activity, increasing the risks of bone destruction and orofacial dysfunction (e.g., impaired mastication, speech difficulties, and craniofacial deformities) [4, 5, 6, 7]. This bone invasion not only creates a self‐reinforcing effect to exacerbate tumor progression, establishing a vicious cycle of “tumor invasion‐bone destruction‐tumor progression,” but also compromises the effectiveness of therapies, such as chemotherapy and immunotherapy, making it challenging to achieve optimal treatment outcomes [1, 2, 8, 9]. Furthermore, solely eradicating malignant tumors is insufficient to fulfill the comprehensive therapeutic objectives, which must extend beyond complete tumor eradication to encompass bone tissue reconstruction, ensuring both oncological control and functional restoration. However, therapeutic approaches focusing on direct tumor killing regardless of the bones closely adjacent to tumor tissues may inadvertently induce osteonecrosis, disrupt bone homeostasis, and impair the ability of bone regeneration, as a consequence of the inappropriate suppression of osteoblast activity by anticancer agents [10, 11, 12, 13, 14]. Such a dilemma underscores the urgent need for tactics capable of concurrent tumor ablation and bone tissue regeneration, which would encourage more comprehensive and effective cancer therapy.

Nanomedicines with the combination of multiple functionalities have offered a unique solution to surmount the aforementioned challenge. By co‐encapsulating diverse therapeutic agents, different therapeutic functions can be integrated into a single nanosystem and be performed under specific stimulation in the pathological environment of tumors (e.g., hypoxia, high levels of particular enzymes, elevated glutathione (GSH), and low pH) [15, 16, 17, 18, 19, 20]. Cytotoxic molecular agents like doxorubicin and paclitaxel are the most commonly delivered drugs for targeted action on tumor lesions. However, the therapeutic efficiency of these drugs is frequently impeded by the occurrence of drug resistance and nonspecific drug diffusion owing to tumor heterogeneity and off‐target expression of biomarkers on healthy cells for lowering targeting capacity [21, 22]. Recently, metal ion interference therapy (MIIT) mediated by metal ion‐containing nanoparticles (NPs) has emerged as an alternative approach for carcinoma treatment, which can induce selective cell death through disrupting intracellular ion homeostasis (e.g., Ca^2^^+^ overload, Fe^2^^+^‐dependent ferroptosis) [23, 24, 25, 26, 27, 28, 29]. Among various metal ions, Ca^2^^+^ and Cu^2^^+^ were deliberately selected due to their distinct yet synergistic roles in modulating biological processes. Specifically, calcium‐based NPs, particularly calcium peroxide (CaO_2_), upon exposure to the intracellular acidic environment would release abundant Ca^2^^+^, which can initiate mitochondrial‐dependent apoptosis through ΔΨm collapse and cytochrome c release [30, 31, 32, 33, 34, 35, 36, 37, 38, 39, 40]. Concurrently, the H_2_O_2_ generated during CaO_2_ decomposition serves as a source of highly cytotoxic radicals, further amplifying mitochondrial injury [41]. On the other hand, Cu^2^^+^ released from copper‐based NPs not only facilitates the conversion of H_2_O_2_ into •OH via a Fenton‐like reaction to create a synergistic effect with CaO_2_, but also exacerbates mitochondrial damage through mitochondrial aggregation and proteotoxic stress, ultimately inducing cuproptosis [42, 43, 44, 45, 46, 47, 48]. The combination of Ca^2^^+^ and Cu^2^^+^ is therefore expected to synergistically amplify the tumor therapeutic effect. By disrupting intracellular ion homeostasis and synergistically damaging mitochondria, this therapeutic strategy leverages the pathological characteristics of tumor cells, offering enhanced tumor specificity and the potential to bypass traditional drug resistance, thereby providing a promising approach for more effective cancer treatment.

As for treating tumor‐associated osteolysis, some agonists and osteoclast inhibitors have been used to stimulate bone regeneration in the area of bone defects [49, 50, 51, 52, 53, 54]. Amongst, osteogenic growth peptide (OGP), a soluble 14‐amino‐acid linear peptide (NH_2_–ALKRQGRTLYGFGG–OH), has been verified as an effective osteogenic induction factor [55]. It exerts direct regulatory effects on osteoblast proliferation, differentiation, and matrix mineralization, showing great potential in treating bone‐related diseases due to its favorable biocompatibility, bioactive function, and convenient modifiability [55, 56, 57, 58, 59]. Pioneering works have engineered various biomaterials, such as porous scaffolds, hydrogels, and polymeric implants, to directly adhere to osseous defect sites and locally deliver OGP for bone regeneration [54, 55, 56, 57, 58, 59]. However, these strategies are only applicable to local lesions and ineffective for diffuse lesions (such as metastatic cancer), which may also increase the risks of trauma and infection, ultimately intensifying the difficulty of tumor treatment. NPs loaded with osteogenic factors can be endowed with the property of bone tissue regeneration and meet the dual functional requirements of antitumor activity and osteogenic stimulation. The key challenge lies in the prerequisite liberation of OGP from NPs prior to tumor cell internalization, followed by its delivery to bone invasion sites to exert precise therapeutic actions.

**SCHEME 1:**
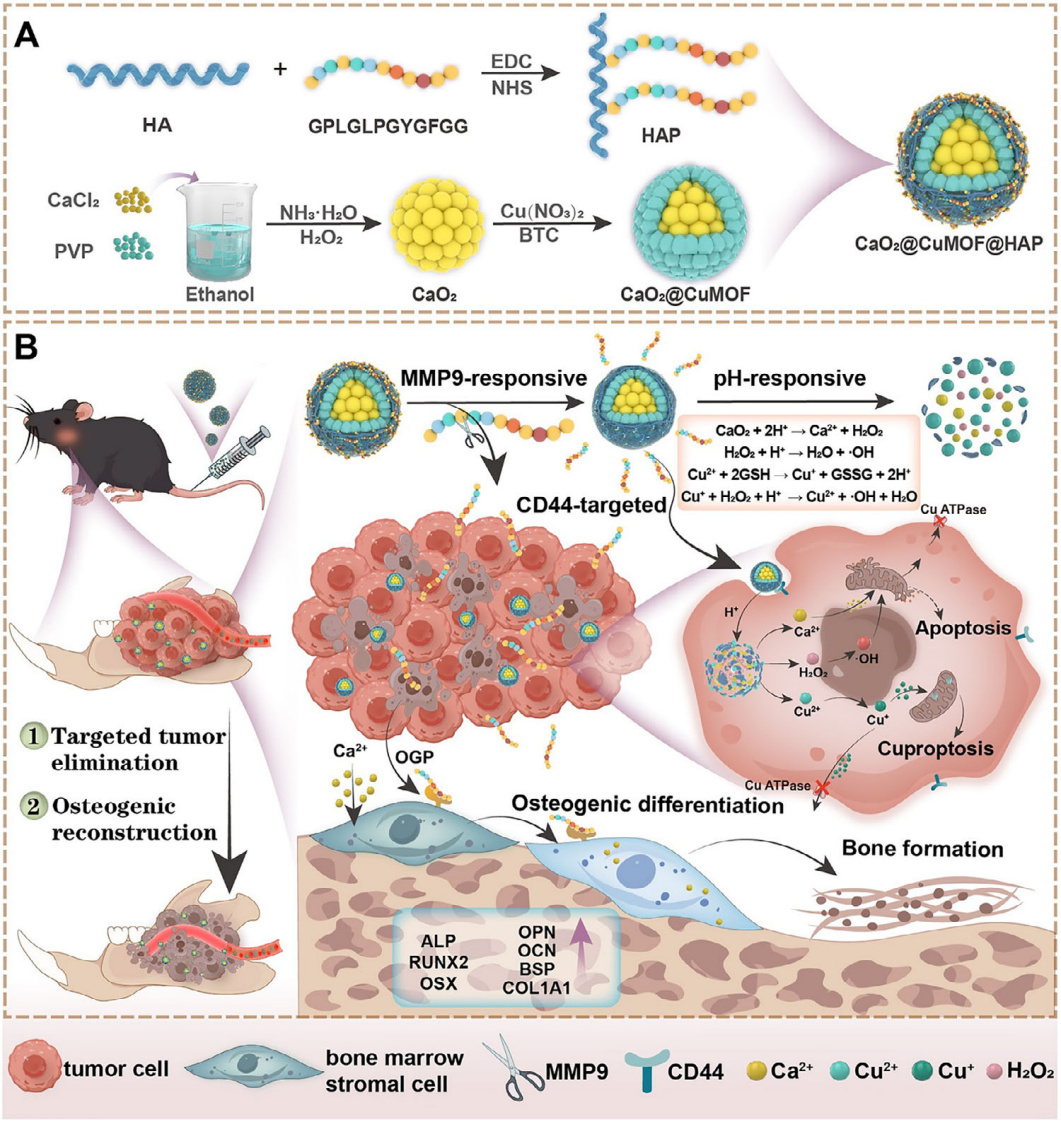
Design of versatile nanohybrid of CaO_2_@CuMOF@HAP for comprehensive tumor treatment. (A) Preparation of stimuli‐responsive CaO_2_@CuMOF@HAP. (B) Schematic illustration of bidirectional mechanisms underlying the simultaneous tumor elimination and tumor‐associated osteolysis restoration of CaO_2_@CuMOF@HAP.

In this study, a versatile nanohybrid (CaO_2_@CuMOF@HAP) with stimuli‐responsive sequential release properties was designed and successfully constructed for simultaneous tumor inhibition and osteogenesis. This CaO_2_@CuMOF@HAP featured a core of bimetallic nanoclusters (CaO_2_@CuMOF) containing dual ions Ca^2+^/Cu^2+^ and surface coated with OGP‐modified hyaluronic acid (HAP) targeting moiety (Scheme 1A). The OGP was conjugated to HA via a matrix metalloproteinase‐9 (MMP9)‐responsive linker for triggered release in MMP9‐enriched tumor microenvironment. Furthermore, the secondary pH‐triggered decomposition of CaO_2_@CuMOF could be achieved in the acidic conditions prevalent in tumor cells (e.g., within lysosomes/endosomes), facilitating the release of Ca^2^^+^ and Cu^2^^+^ ions for MIIT. The resultant nanohybrid demonstrated the potency of selectively accumulating at tumor sites for the first‐step release of OGP, which had a much smaller size that could deeply penetrate into the tumor‐ infiltrated bone destruction area to promote osteogenic differentiation and maturation. Upon specific recognition and endocytosis by tumor cells, CaO_2_@CuMOF@HAP underwent intracellular decomposition, thereby triggering the release of substantial amounts of Ca^2+^ (which activated mitochondrial‐dependent apoptosis) and Cu^2+^ (that caused significant cuproptosis), synergistically promoting tumor cell death. The bidirectional action mechanism of antitumor and osteogenic effects was investigated in the orthotopic buccal tumor mouse model with mandibular bone invasion (Scheme 1B). Collectively, such a multifunctional nanohybrid acting on two therapeutic targets has the potential to offer an effective strategy for treating tumors with severe bone invasion.

## 2 Results and Discussion

### 2.1 Preparation and Characterization of CaO_2_@CuMOF@HAP

**FIGURE 1:**
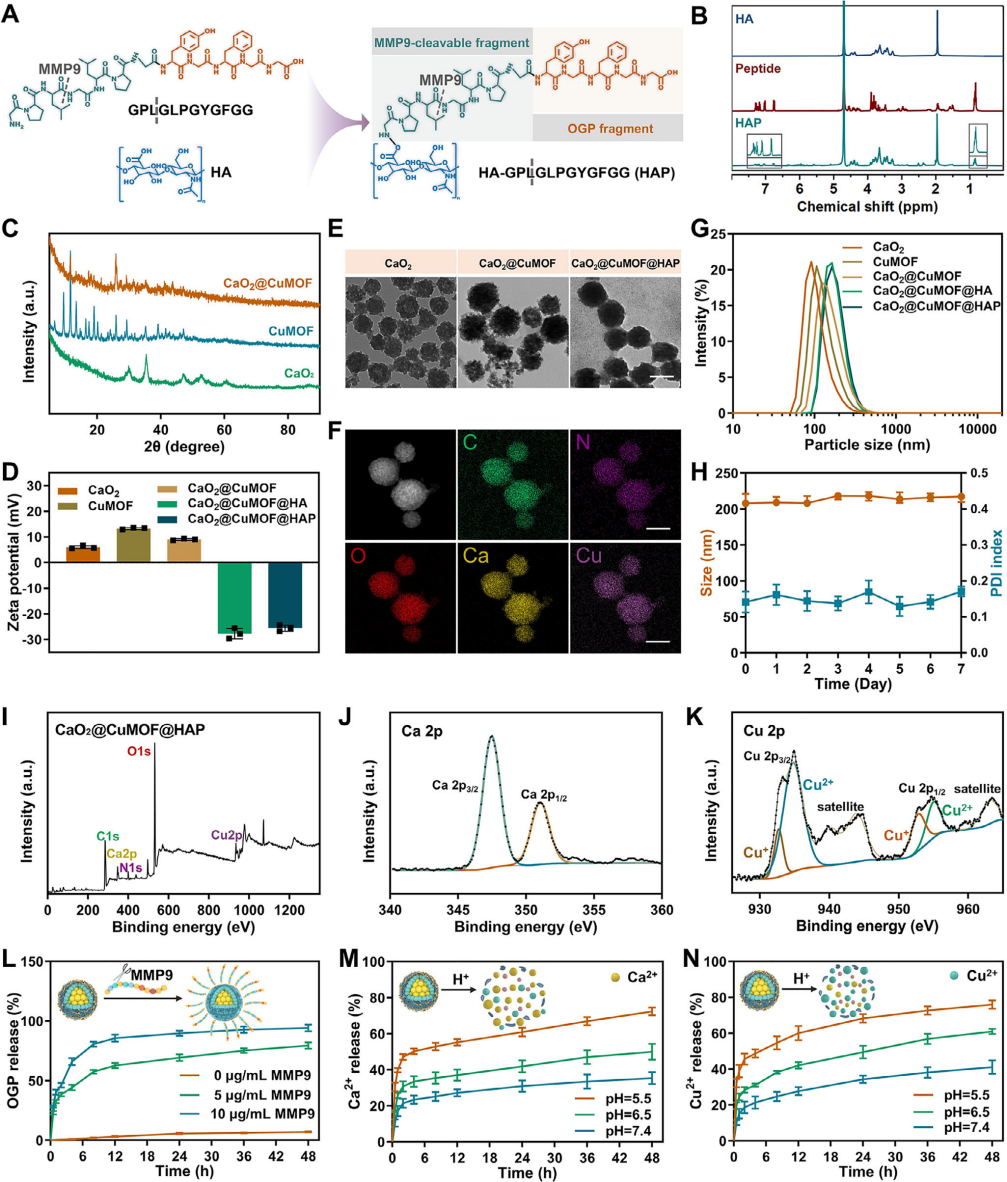
Preparation and characterization of CaO_2_@CuMOF@HAP. (A) Schematic illustration of the preparation of peptide‐modified hyaluronic acid (HAP). (B) ^1^H NMR spectra of HA, peptide, and HAP. (C) XRD patterns of CaO_2_, CuMOF, and CaO_2_@CuMOF. (D) Zeta potentials of CaO_2_, CuMOF, CaO_2_@CuMOF, CaO_2_@CuMOF@HA, and CaO_2_@CuMOF@HAP. (E) TEM images of CaO_2_, CaO_2_@CuMOF, and CaO_2_@CuMOF@HAP. Scale bar: 100 nm. (F) Energy dispersive spectrometer (EDS) element mapping images of CaO_2_@CuMOF@HAP. Scale bar: 100 nm. (G) Hydrodynamic diameters of CaO_2_, CuMOF, CaO_2_@CuMOF, CaO_2_@CuMOF@HA, and CaO_2_@CuMOF@HAP. (H) Hydrodynamic diameters and PDI index of CaO_2_@CuMOF@HAP in PBS (10 mm, pH 7.4) at room temperature over various time intervals. (I) XPS spectrum of CaO_2_@CuMOF@HAP. High‐resolution XPS spectrum of (J) Ca and (K) Cu. (L) Release profiles of OGP from CaO_2_@CuMOF@FITC‐HAP over time in PBS (10 mm, pH 7.4) with different concentrations of MMP9 protein. Release profiles of (M) Ca^2+^ and (N) Cu^2+^ from CaO_2_@CuMOF@HAP over time at different pH values (pH 5.5, 6.5, and 7.4). Data were performed as the mean ± SD (*n* = 3). Two‐way ANOVA with Tukey's post hoc test was used for multiple comparisons in (L), (M), and (N).

To obtain a surface‐coating polymer with both osteogenic and tumor targeting properties, the naturally occurring glycosaminoglycan hyaluronic acid (HA) that can bind to the cluster of differentiation 44 (CD44) receptor on tumor cells was used to be modified with a responsive peptide consisting of an MMP9‐cleavable fragment (GPLGLPG) and an OGP fragment (YGFGG). Specifically, the peptide with the sequence of NH_2_–GPLGLPGYGFGG–OH was grafted onto HA through the condensation reaction between the terminal amino groups of the peptide and the carboxyl groups of HA activated by *N*‐hydroxysuccinimide/*N*‐(3‐dimethylaminopropyl)/*N*′‐ethylcarbodiimide hydrochloride (NHS/EDC), resulting in the formation of peptide‐modified HA (HAP) (Figure 1A). As characterized by ^1^H nuclear magnetic resonance (NMR) spectroscopy, the distinctive peak at 0.82 ppm assigned to hydrogen atoms of the methyl groups and peaks at 6.76–7.22 ppm corresponded to the hydrogen atoms on the benzene ring from the coupled peptide appeared in the spectrum of HAP, as compared with the individual spectra of HA (Figure 1B), demonstrating the successful modification of functional peptide on HA polymer. Based on this, the substitution degree of peptide onto HA was quantified to be 12.67% ± 0.67%. Thereafter, the core nanoparticles of CaO_2_@CuMOF were synthesized by a bottom‐up approach. The powder X‐ray diffraction (XRD) analysis was carried out to explore the crystal structure of synthesized nanoparticles, from which the synthesized CaO_2_ nanoparticles showed a striking similarity in diffraction patterns as those of the tetragonal CaO_2_ standard card (PDF#03‐0865) (Figure S1), confirming the successful crystallization of CaO_2_ cores. In addition, characteristic diffraction peaks of CuMOF appeared in the hybrid nanostructure of CaO_2_@CuMOF (Figure 1C), verifying the integration of CaO_2_ and CuMOF. The obtained CaO_2_@CuMOF exhibited a positively charged surface (ζ‐potential: +9.0 ± 0.4 mV) (Figure 1D), making it suitable for subsequent coating with negative substances. Therefore, the deposition of HAP onto the outer surface of CaO_2_@CuMOF was achieved by mixing the two components under gentle stirring, forming the multifunctional nanohybrid CaO_2_@CuMOF@HAP. Following this, the nanoparticle surface became negatively charged (ζ‐potential: −25.6 ± 1.2 mV), which would be beneficial in resisting protein adsorption in biological systems, thereby promoting prolonged circulation time.

Transmission electron microscopy (TEM) images revealed that, following the stepwise preparation, CaO_2_ nanocrystals assembled into uniformly sized and controllable spherical aggregates, and smaller nanocrystals were observed surrounding the CaO_2_ cores in CaO_2_@CuMOF, leading to a more compact nanostructure and an increasingly irregular morphology (Figure 1E). After coating with HAP, CaO_2_@CuMOF@HAP nanoparticles exhibited a more rounded and regular morphology with a nanoscale size around 120 nm. On the basis of energy dispersive spectrometer (EDS) mapping, the colocalization of multiple elements including C, N, O, Ca, and Cu was identified within the CaO_2_@CuMOF@HAP nanocomposite (Figure 1F), which confirmed the hybridization of different components into a single nanoplatform. Dynamic light scattering (DLS) analysis revealed a gradual increase in the hydrodynamic diameter from 192.6 ± 2.4 nm (for CaO_2_@CuMOF) to 214.0 ± 8.1 nm (for CaO_2_@CuMOF@HAP), with all nanoparticles exhibiting a consistently narrow size distribution (polydispersity index (PDI) < 0.2) (Figure 1G), indicating the excellent suitability of CaO_2_@CuMOF@HAP for biological applications. To evaluate colloidal stability, the resultant CaO_2_@CuMOF@HAP was dispersed in phosphate buffered saline (PBS, 10 mm, pH 7.4) and incubated at room temperature. The hydrodynamic size and PDI of the nanosystem were monitored over 7 days using DLS. As shown in Figure 1H, negligible changes in both size and PDI were observed throughout the 7‐day period, with no visible precipitation. These results demonstrate the excellent colloidal stability of CaO_2_@CuMOF@HAP. Furthermore, the X‐ray photoelectron spectroscopy (XPS) spectrum of CaO_2_@CuMOF@HAP revealed the elemental state of the nanocomposite (Figure 1I). From the high‐resolution XPS spectrum of Ca 2p, two characteristic peaks at 347.47 and 351.05 eV for Ca 2p_3/2_ and Ca 2p_1/2_ were detected (Figure 1J), and the observed peaks at 531.41 and 532.9 eV in the O 1s spectrum could be attributed to C═O and O─O bonds (Figure S2), which demonstrated the presence of HAP and peroxy group. Additionally, the Cu 2p spectrum in Figure 1K exhibited two strong satellites at 943.93 and 963.01 eV, along with the spin–orbit split energy of Cu 2p_3/2_ and Cu 2p_1/2_ at 932.70/934.78 eV and 952.74/954.97 eV, indicating the coexistence of Cu^+^ and Cu^2^^+^ oxidation states in CaO_2_@CuMOF@HAP. Moreover, the presence of organic components in CaO_2_@CuMOF@HAP was also demonstrated by the observation of C─C, C─N, and C═O bonds as well as C─N and N─H bonds in the XPS spectra of C 1s (Figure S3) and N 1s (Figure S4), respectively.

Given that an MMP9‐cleavable fragment was used as a bridging linkage to modify OGP fragment onto HA for obtaining CaO_2_@CuMOF@HAP, OGP was expected to be released in an MMP9‐rich environment, such as the tumor microenvironment, to exert its biological functions after reaching tumor‐invaded bone tissue. To confirm this, the OGP release under the trigger of MMP9 was monitored by using the nanohybrid constructed with FITC‐labeled OGP (denoted as CaO_2_@CuMOF@FITC‐HAP). As shown in Figure 1L, according to the standard curve of FITC‐labeled OGP (Figure S5), efficient OGP release was detected in the presence of MMP9, and the release rate enhanced with the MMP9 concentration increasing, resulting in over 90% of OGP being liberated after post‐treatment with MMP9 (10 µg/mL) for 48 h. Such a triggered release behavior is primarily due to the efficient MMP9‐mediated cleavage of sequence‐specific domain to facilitate the separation of OGP from the nanohybrid. In contrast, a very limited amount of free OGP (< 10%) was released from CaO_2_@CuMOF@FITC‐HAP in the absence of MMP9 owing to the firm bonding of OGP on the nanoparticles. Additionally, it is well demonstrated that CaO_2_ and CuMOF can be decomposed in acidic environment to generate Ca^2+^ and Cu^2+^ [40]. Therefore, CaO_2_@CuMOF@HAP was expected to exhibit acid‐responsive dissociation profiles while remaining stable under physiological conditions. As displayed in Figure 1M,N, a markedly accelerated pH‐triggered release of Ca^2+^ and Cu^2+^ from CaO_2_@CuMOF@HAP was detected in solutions with low pH values (6.5 and 5.5), compared with that in the neutral condition (pH 7.4). Since the tumor microenvironment (with pH around 6.8) and lysosomes (with pH around 5.0) are both slightly acidic, this pH‐controlled release manner can promise the precise delivery of therapeutic agents for targeted therapy in the lesioned tumor region, thereby effectively reducing nontargeted side effects on normal tissues.

### 2.2 Cellular Uptake and Tumor Cell Killing Effects In Vitro

**FIGURE 2:**
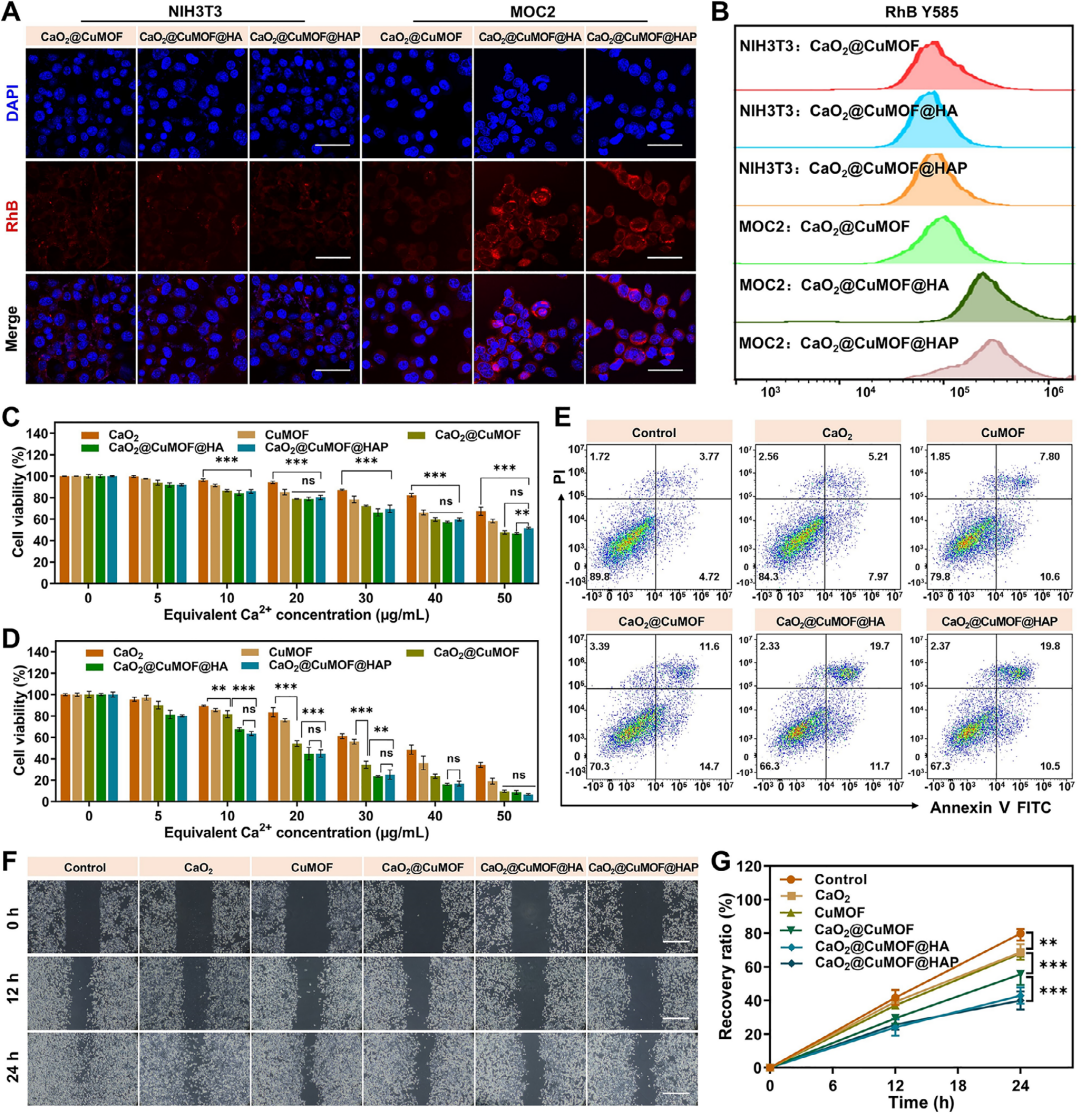
Cellular uptake and tumor cell killing effects in vitro. (A) CLSM images and (B) flow cytometry analysis of NIH3T3 cells and MOC2 cells treated with rhodamine B‐labeled CaO_2_@CuMOF(RhB), CaO_2_@CuMOF@HA(RhB), and CaO_2_@CuMOF@HAP(RhB) for 4 h. Red: rhodamine B; Blue: DAPI‐stained nuclei. Scale bar: 50 µm. Cell viability analysis of (C) NIH3T3 cells and (D) MOC2 cells with different treatments for 24 h. (E) Flow cytometry analysis of apoptosis/necrosis of MOC2 cells with different treatments for 24 h. (F) Cell migration images of MOC2 cells with different treatments. Scale bar: 500 µm. (G) Cell migration ratios of MOC2 cells with different treatments. Data were performed as the mean ± SD (*n* = 3). Two‐way ANOVA with Tukey's post hoc test was used for multiple comparisons in (C), (D), and (G). *p < 0.05, **p < 0.01, ***p < 0.001.

Taking advantage of HA's binding affinity toward CD44 receptor, which is abundantly localized on malignant cell membranes, CaO_2_@CuMOF@HAP is anticipated to exhibit favorable tumor targeting ability. To confirm this, the cellular uptake efficiency was investigated in CD44‐negative NIH3T3 cells (murine fibroblast cell line) and CD44‐positive MOC2 cells (murine oral squamous cell carcinoma cell line), respectively. For direct confocal laser scanning microscope (CLSM) observation, nanoparticles without (CaO_2_@CuMOF) or with HA modification (CaO_2_@CuMOF@HA and CaO_2_@CuMOF@HAP) were labeled with the fluorescent dye rhodamine B (RhB) to noninvasively image the intracellular uptake. As displayed in Figure 2A, it was difficult to detect red fluorescence intracellularly in all treated groups after incubation with NIH3T3 cells for 4 h, and no significant difference in fluorescence intensity following the treatments of either HA‐coated nanoparticles or HA‐lacking ones was observed, indicating the inefficient cellular uptake of all nanotherapeutics by normal cells. In contrast, the CD44‐overexpressed MOC2 cells demonstrated markedly elevated fluorescence signals upon treatment with HA‐coated nanoparticles relative to the non‐HA CaO_2_@CuMOF(RhB)‐treated group. Also, the treatment of HA‐coated nanoparticle toward MOC2 cells displayed a much stronger fluorescent signal relative to NIH3T3 cells, demonstrating that HA modification could endow nanoparticles with favorable tumor cell selectivity. Similar trends were found via flow cytometric evaluation, which showed a 2.30‐fold increase in mean fluorescence intensity (MFI) in CaO_2_@CuMOF@HA(RhB)‐ and CaO_2_@CuMOF@HAP(RhB)‐treated MOC2 cells, compared with those treated with CaO_2_@CuMOF(RhB) (Figure 2B; Figure S6). Notably, no comparable fluorescence enhancement was observed in normal cell line, which maintained consistently low MFI levels across all treatment groups. These results strongly indicate that the designed CaO_2_@CuMOF@HAP undergoes preferential internalization by tumor cells, thereby enabling the targeted delivery of tumor‐specific therapeutic agents.

Following efficient cellular internalization by tumor cells, the antitumor capability of CaO_2_@CuMOF@HAP in vitro was systematically investigated. The cytotoxic effect was evaluated on both NIH3T3 and MOC2 cells by cell counting kit‐8 (CCK‐8) assay. For NIH3T3 cells treated with various nanoformulations, the cell viability maintained consistently high levels, even when exposed to HA‐coated nanoparticles at equivalent Ca^2+^ concentrations up to 50 µg/mL (survival ratio: > 50%) (Figure 2C). It should be ascribed to the feeble cellular uptake by CD44‐negative cells, which limits the in situ production of highly cytotoxic Ca^2+^/Cu^2+^ species within cells. In contrast, for MOC2 cells (Figure 2D), CaO_2_ and CuMOF manifested a concentration‐dependent cytotoxicity, albeit at a relatively low level, owing to their intrinsic cell death‐inducing ability of these two kinds of nanoparticles. The number of dead cells was greatly increased by the treatment of CaO_2_@CuMOF, which integrated the two components to achieve a synergistic cytotoxic response. Furthermore, by coating nanoparticles with HA, CaO_2_@CuMOF@HA manifested a dramatically improved cell‐killing efficacy because of the facilitated intracellular uptake of the nanohybrid by MOC2 cells. Similar cytotoxic effects were observed for CaO_2_@CuMOF@HAP nanoparticles, resulting in a substantial increase in therapeutic potency.

To elucidate the cell death patterns of MOC2 cells following exposure to diverse treatments, annexin V‐FITC/propidium iodide (PI) staining of apoptotic/necrotic cells was quantified by flow cytometry (Figure 2E; Figure S7). Compared with the low fraction of apoptotic cells in PBS group, CaO_2_ and CuMOF individually led to slightly higher apoptosis proportions, with 13.32% ± 0.89% and 17.60% ± 1.08% of the cells in apoptotic status, respectively. For CaO_2_@CuMOF‐treated cells, the percentage of apoptosis/necrosis increased to 24.83% ± 2.20%, indicating the combined effect on the therapeutic response. In case of CaO_2_@CuMOF@HA‐ and CaO_2_@CuMOF@HAP‐treated cells, a markedly elevated apoptosis‐inducing capacity was detected (∼ 34% of cellular apoptosis), signifying the importance of additional HA modification in boosting therapeutic efficiency.

To assess the impact of CaO_2_@CuMOF@HAP on tumor cell invasion and metastasis, we further evaluated the migratory property of MOC2 cells by a wound‐healing test. As displayed in Figure 2F, cells in PBS group exhibited excellent cell migration, with numerous cells moving directionally into the pre‐created denuded area to effectively close the “wound” within 24 h. For cells coincubated with CaO_2_, CuMOF, CaO_2_@CuMOF, CaO_2_@CuMOF@HA, and CaO_2_@CuMOF@HAP, the cell migration was progressively declined, with only a few cells migrated across the wound edges ultimately. As quantified in Figure 2G, the recovery ratio of the wound area was calculated to be remarkably decreased from 79.31% (blank control) to 39.98% (cells treated with CaO_2_@CuMOF@HAP), further demonstrating the great potential of CaO_2_@CuMOF@HAP for blocking the invasive tumor cell behaviors.

### 2.3 Antitumor Mechanism Evaluated In Vitro

**FIGURE 3:**
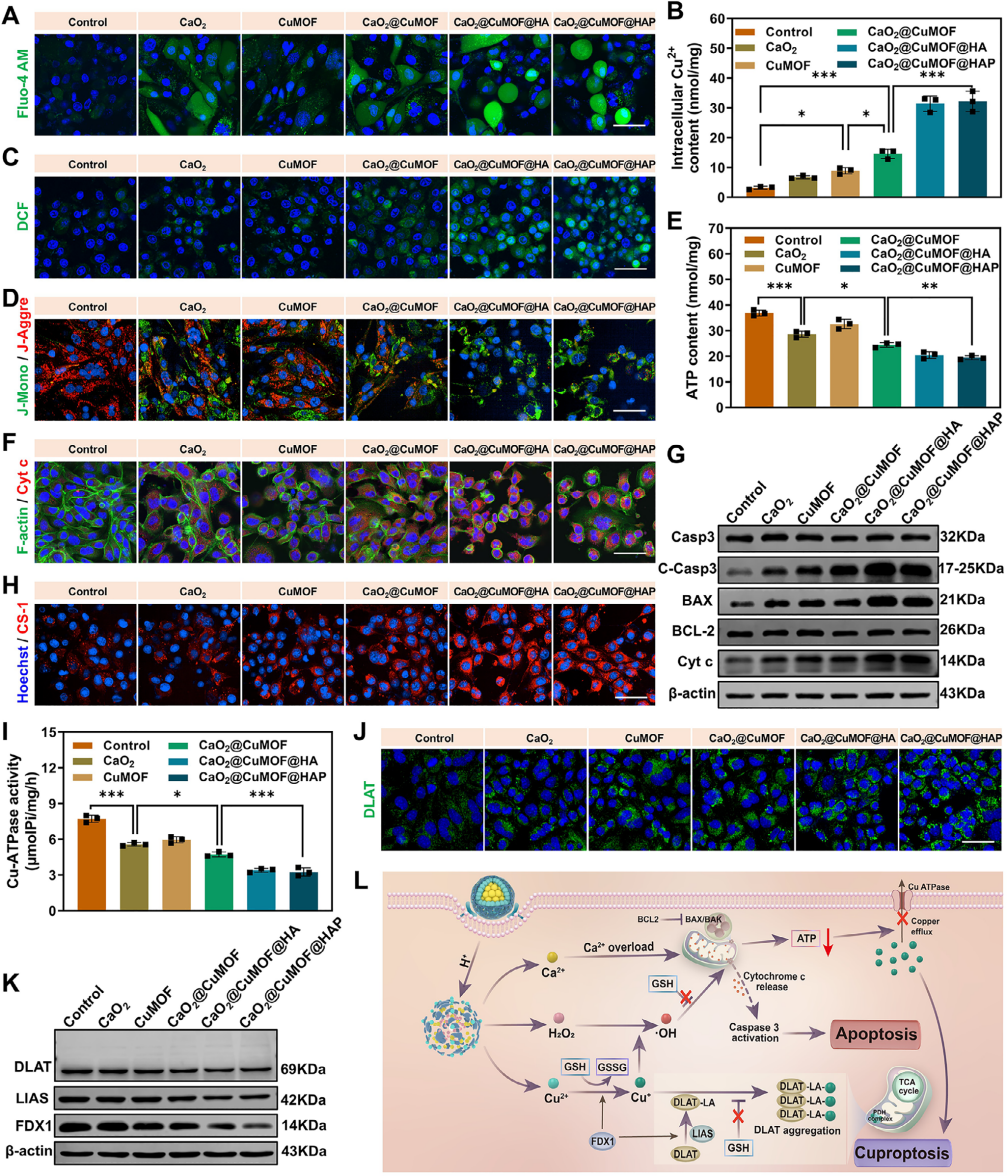
Antitumor mechanism evaluated in vitro. (A) Intracellular Ca^2+^ in MOC2 cells detecting with Fluo‐4 AM after various treatments. Scale bar: 50 µm. (B) Intracellular Cu^2+^ levels of MOC2 cells. (C) Intracellular reactive oxygen species (ROS) in MOC2 cells detecting with DCFH‐DA. Scale bar: 50 µm. (D) Mitochondrial membrane potential of MOC2 cells assessed by JC‐1. Green: JC‐1 monomers; Red: JC‐1 aggregates; Blue: Hoechst 33342‐stained nuclei. Scale bar: 50 µm. (E) Intracellular ATP levels of MOC2 cells. (F) IF staining analysis of Cyt c expression. (G) Western blot analysis of caspase 3, cleaved caspase‐3, BCL‐2, BAX, and Cyt c. (H) Intracellular Cu^+^ in MOC2 cells detecting with CS‐1. Scale bar: 50 µm. (I) The Cu‐ATPase activity of MOC2 cells. (J) IF staining analysis of DLAT aggregates. (K) Western blot analysis of DLAT, LIAS, and FDX1. (L) Schematic illustration of CaO_2_@CuMOF@HAP inducing cascade mitochondrial dysfunction through dual apoptosis and cuproptosis pathways. Data were performed as the mean ± SD (*n* = 3). One‐way ANOVA with Tukey's post hoc test was used for multiple comparisons in (B), (E), and (I). *p < 0.05, **p < 0.01, ***p < 0.001.

Encouraged by the promising antitumor effects observed at the cellular level, the underlying therapeutic mechanisms were further explored in MOC2 cells. Given that the CaO_2_@CuMOF core of CaO_2_@CuMOF@HAP decomposes under intracellular acidic conditions (e.g., lysosomes/endosomes) to release Ca^2+^ and Cu^2+^, the intracellular ion levels were assessed after cellular internalization. As displayed in Figure 3A, using Fluo‐4 acetoxymethyl ester (Fluo‐4 AM) for intracellular Ca^2+^ level detection, cells treated with CaO_2_ and CaO_2_@CuMOF presented enhanced green fluorescence compared to the weak fluorescence observed in the control group. The brightest green fluorescence signal exhibited in cells treated with HA‐bearing nanoparticles. These findings were further validated through flow cytometric assessment of Fluo‐4 AM fluorescence intensity (Figure S8). Additionally, a calcium colorimetric assay was carried out to precisely quantify intracellular Ca^2+^ levels. The results confirmed that CaO_2_@CuMOF@HA and CaO_2_@CuMOF@HAP groups exhibited a 3.49‐fold and a 3.44‐fold change relative to the control group (Figure S9). Moreover, the intracellular copper accumulation was quantified using a copper colorimetric assay kit. As displayed in Figure 3B, the CuMOF group exhibited a 2.81‐fold increase (vs. control group) in intracellular Cu^2+^ concentration, which reached the highest level after incubation with HA‐bearing nanoparticles, owing to their tumor‐targeting capability and acid‐triggered decomposition for efficient copper delivery into MOC2 cells. During the acid‐mediated decomposition of CaO_2_@CuMOF@HAP, H_2_O_2_ could also be generated in the CaO_2_‐involved reaction, while the liberated Cu^2+^ could be reduced by intracellular GSH to Cu^+^ for catalyzing the subsequent production of •OH radicals. These oxidizing substances are typical reactive oxygen species (ROS) for causing severe cell damage. Therefore, we analyzed the ROS levels in cells under different treatments using a dichlorodihydrofluorescein diacetate (DCFH‐DA) probe, which can be rapidly hydrolyzed after entering cells and oxidized into green fluorescent DCF for direct imaging. Compared with the blank control, we could figure out that the green fluorescence was remarkably increased by the treatment of CaO_2_, and additionally enhanced by incubating the cells with CaO_2_@CuMOF (Figure 3C), attributing to the cooperative effect of the two ROS generators. Moreover, HA‐modified nanoparticles (CaO_2_@CuMOF@HA and CaO_2_@CuMOF@HAP) further reinforced the intracellular green fluorescence, giving rise to nearly tenfold higher MFI in cells relative to the nontargeted group (Figure S10).

Since the Ca^2+^ overload and excessive production of ROS by pH‐triggered CaO_2_@CuMOF@HAP degradation are able to induce direct mitochondria dysfunction, we further evaluated the mitochondrial status of MOC2 cells using the 5,5′,6,6′‐tetrachloro‐1,1′,3,3′‐tetraethylbenzimidazolocarbocyanine iodide (JC‐1) probe, which selectively forms red‐fluorescent aggregates in healthy mitochondria while remains as green‐fluorescent monomers in damaged mitochondria with decreased membrane potential. Confocal images showed that cells treated with CaO_2_ displayed a shift from red‐fluorescent aggregates to green‐fluorescent monomers, indicating mitochondrial membrane depolarization (Figure 3D). This effect was further enhanced in the CaO_2_@CuMOF group and became the most pronounced in the CaO_2_@CuMOF@HA and CaO_2_@CuMOF@HAP groups, suggesting progressively aggravated mitochondrial dysfunction and severe cellular damage. Consistent with this observation, intracellular ATP assay demonstrated a ∼ 50% reduction in ATP levels in CaO_2_@CuMOF@HAP‐treated MOC2 cells (vs. the control group) (Figure 3E). This insufficient ATP production further manifested a serious mitochondrial damage. To investigate the effects of our nanosystem on mitochondrial function, we performed Seahorse XF analysis. As shown in Figure S11, CaO_2_ treatment significantly suppressed basal respiration in MOC2 cells, resulting in an approximate 35% reduction compared to the control group, while CuMOF led to a slight decrease of about 18%. These results indicate that although both CaO_2_ and CuMOF influence basal mitochondrial respiration, CaO_2_ exerts a more pronounced inhibitory effect. In terms of ATP‐linked respiration, which reflects mitochondrial ATP production capacity, CaO_2_ treatment reduced the level by approximately 40% relative to the control group, and the CaO_2_@CuMOF treatment further decreased ATP‐linked respiration by about 50%. These results suggest that CaO_2_ impairs oxidative phosphorylation and that this damaging effect is exacerbated by the presence of Cu^2^^+^. Furthermore, maximal respiration was reduced by approximately 32% in the CaO_2_‐treated group and 28% in the CuMOF‐treated group, indicating impaired electron transport chain function. Taken together, these findings reveal that our nanomaterials, especially the CaO_2_ component, negatively regulate mitochondrial function, leading to reduced ATP production. As mitochondria serve as the central regulatory hub of programmed cell death, which play pivotal roles in the intrinsic apoptotic pathway involving pore formation in the mitochondrial outer membranes, leading to cytochrome c (Cyt c) release and subsequent caspase activation [30]. Therefore, key markers related to mitochondrial apoptotic pathway were detected. Immunofluorescence (IF) staining of Cyt c revealed a significantly upregulated expression in the cells treated with CaO_2_@CuMOF@HAP (Figure 3F). Additional apoptotic markers such as the activated executioner proteases of cleaved caspase‐3, the proapoptotic protein BAX and the antiapoptotic protein BCL‐2 were analyzed through western blotting. Results showed that CaO_2_@CuMOF@HAP treatment markedly upregulated proapoptotic proteins (cleaved caspase‐3 and BAX) levels while slightly suppressed BCL‐2 levels in MOC2 cells (Figure 3G; Figure S12), confirming the induction of mitochondrial apoptosis.

Recent studies have elucidated a copper‐dependent cell death modality characterized by mitochondrial proteotoxic stress and abnormal aggregation of lipoylated proteins in the tricarboxylic acid cycle, named “cuproptosis,” which is quite distinct from the canonical copper ion toxicity driven by ROS‐mediated oxidative damage [42, 43, 44, 45]. In order to investigate whether the emerging programmed cell death of cuproptosis plays a crucial role in the cell damage induced by our designed nanohybrid, the intracellular Cu^+^, which is able to induce the lipoylated protein aggregation, iron–sulfur cluster protein loss for eliciting cuproptosis, was quantified using Cu^+^‐specific fluorescent probe coppersensor‐1 (CS‐1). As displayed in Figure 3H, elevated Cu^+^ contents were detected in cells treated with CaO_2_@CuMOF@HAP, as evidenced by strong red fluorescence in CLSM images and a 2.62‐fold increase in MFI via flow cytometry (Figure S13), confirming substantial Cu^+^ accumulation in CaO_2_@CuMOF@HAP‐treated cells. Notably, this exogenous copper influx would disrupt cellular copper homeostasis, which was cooperatively exacerbated by CaO_2_@CuMOF@HAP‐induced suppression of copper‐transporting ATPases (key regulators of intracellular copper balance) (Figure 3I) owing to the mitochondrial dysfunction‐induced ATP reduction. Besides, after treatment with CaO_2_@CuMOF@HAP, the intracellular reducing agent GSH, which could bind to unstable Cu^+^ and alleviate its toxicity, was depleted exceeding 55% in tumor cells (Figure S14). Immunofluorescence analysis of lipoylated DLAT aggregates (Figure 3J) revealed that treatment with CuMOF‐induced pronounced formation of DLAT foci, a hallmark cytological characteristic of cuproptosis, providing direct evidence of copper‐dependent protein aggregation. Notably, this effect was further enhanced in cells treated with CaO_2_@CuMOF and reached the highest level in the CaO_2_@CuMOF@HA and CaO_2_@CuMOF@HAP groups, where the most abundant DLAT aggregation was observed. These findings provide compelling evidence for copper‐dependent protein aggregation and confirm the induction of cuproptosis. Furthermore, key components of the cuproptosis signaling pathway, including FDX1, DLAT monomer, and LIAS, were significantly downregulated upon CaO_2_@CuMOF@HAP treatment (Figure 3K; Figures S15 and S16), collectively confirming the activation of cuproptosis.

Altogether, it can be concluded that CaO_2_@CuMOF@HAP, even without the loading of cytotoxic drugs, activates the intrinsic apoptotic pathway via calcium overload and excessive oxidative stress, and triggers cuproptosis for inducing direct cell death, establishing a potent strategy by using dual ions‐containing nanohybrid for efficient tumor therapy (Figure 3L).

### 2.4 Osteogenesis Promotion In Vitro

**FIGURE 4:**
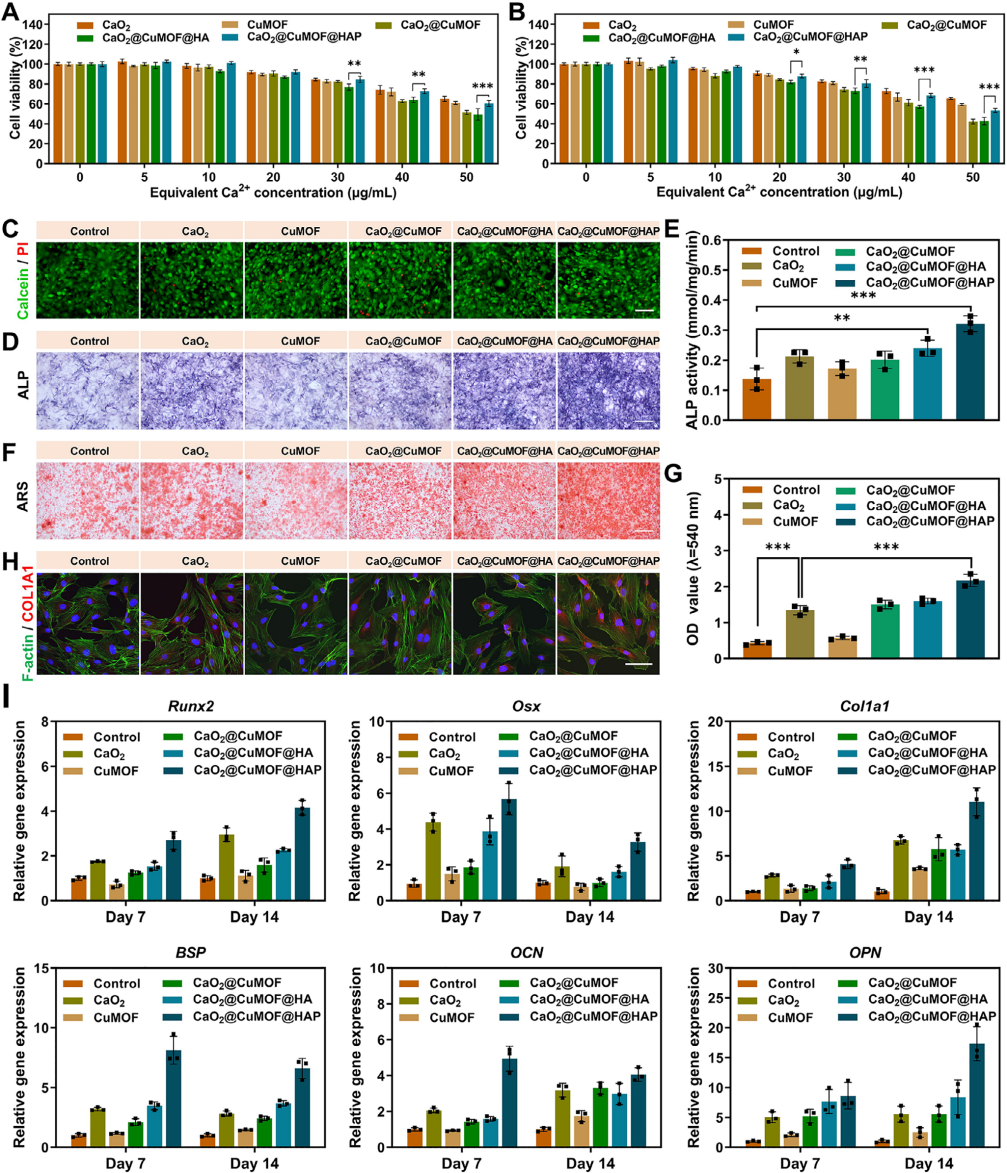
Osteogenesis promotion in vitro. (A) Cell viability of MC3T3 cells with different treatments for 24 h. (B) Cell viability of MC3T3 cells with different treatments for 72 h. (C) Calcein/PI staining of MC3T3 cells after various treatments for 72 h. Scale bar: 100 µm. (D) ALP staining and (E) quantified ALP activity of MC3T3 cells after various treatments for 7 days. Scale bar: 500 µm. (F) ARS staining of MC3T3 cells after various treatments for 21 days. Scale bar: 500 µm. (G) Quantitative analysis of calcium nodules after various treatments for 21 days. (H) IF staining of COL1A1 expression. Scale bar: 50 µm. (I) The qRT‐PCR analysis of the relative expression levels of *Runx2*, *Osx*, *Col1a1*, *BSP*, *OPN*, and *OCN*. Data were performed as the mean ± SD (*n* = 3). Two‐way ANOVA with Tukey's post hoc test was used for multiple comparisons in (A) and (B). One‐way ANOVA with Tukey's post hoc test was used for multiple comparisons in (E) and (G). *p < 0.05, **p < 0.01, ***p < 0.001.

To investigate the osteogenic regenerative capacity of CaO_2_@CuMOF@HAP, one of the most commonly used murine osteoblast‐like cell lines (MC3T3 cells) was used for osteogenesis evaluation. CCK‐8 assays demonstrated excellent biocompatibility of CaO_2_@CuMOF@HAP, with > 50% of MC3T3 cells remaining viable even at high Ca^2^^+^ equivalent concentrations (50 µg/mL) following both 24 and 72 h treatments (Figure 4A,B). This finding was further corroborated by calcein‐AM/PI double staining, where minimal red fluorescence was visualized, validating an extremely small proportion of dead cells (Figure 4C; Figure S17). These results highlight the outstanding cytocompatibility of CaO_2_@CuMOF@HAP, which is favorable for osteogenic applications. After that, we used an optimally designed transwell coculture system to simulate the tumor‐bone microenvironment and assessed the uptake of OGP by MC3T3 cells. As illustrated in Figure S18A, MC3T3 cells were seeded in the lower chamber, while MOC2 cells embedded in matrigel were placed in the upper chamber to mimic the tumor and its extracellular matrix. CaO_2_@CuMOF@FITC‐HAP was added to the upper chamber medium under three conditions: (1) 0 µg/mL MMP9, (2) 5 µg/mL MMP9, and (3) 10 µg/mL MMP9. After coincubation for 24 h, OGP uptake by MC3T3 cells in the lower chamber was evaluated using confocal microscopy. Confocal images showed that the uptake of FITC‐labeled OGP by MC3T3 cells was enhanced in an MMP9‐dependent manner. Specifically, compared with the 0 µg/mL control group, the fluorescence intensity in the 5 µg/mL MMP9 treatment group was significantly increased, and the increase was the most obvious in the 10 µg/mL group (Figure S18B). These findings suggest that MMP9‐mediated cleavage enhances the transport of OGP across the tumor barrier, thereby increasing its accessibility to cells in the underlying bone region.

Subsequently, alkaline phosphatase (ALP) activity, a well‐established early‐stage osteogenic differentiation marker for osteoblast maturation and calcification, was assessed at 7 days post‐osteogenic induction. The blue‐purple coloration in both control and CuMOF groups exhibited limited staining intensity and spatial distribution, and became significantly deeper and more extensive by the treatment of Ca^2+^‐containing nanoformulations (CaO_2_, CaO_2_@CuMOF, and CaO_2_@CuMOF@HA), revealing the increased expression levels of ALP (Figure 4D). With the additional integration of OGP into the nanosystem, the strongest staining intensity was observed in CaO_2_@CuMOF@HAP group, attributed to the osteoinductive ability of OGP for amplifying the osteogenic differentiation. The quantified ALP activity of cells treated with CaO_2_@CuMOF@HAP was 2.34‐fold higher than in the control ones (*p* < 0.001), further validating its superior osteoinductive potential (Figure 4E). Furthermore, alizarin red S (ARS) staining was performed to visualize mineralized nodules and evaluate calcium accumulation along with mineralization. Consistent with above ALP staining results, after 21 days post‐osteogenic induction, numerous orange‐red clusters were observed in the CaO_2_@CuMOF@HAP group via ARS–calcium chelation, which manifested the abundant formation of calcified nodules (Figure 4F). The mineralized nodules were quantified by dissolving the calcium deposits with 10% chlorohexadecyl pyridine followed by determining absorbance values at 540 nm. Results showed that the multifunctional CaO_2_@CuMOF@HAP exhibited 1.62‐fold potentiated capacity for stimulating calcium nodule formation compared with the non‐functional CaO_2_‐treated group and a 5.08‐fold enhancement compared with the control group (Figure 4G). Besides, to compare the bioactivity of free OGP and OGP released from the nanohybrid following MMP9 cleavage, we collected supernatants from CaO_2_@CuMOF@HAP incubation media with or without MMP9 (10 µg/mL) for the subsequent osteogenic induction assay. Free OGP was used as a positive control, while PBS treatment served as a negative control. Osteogenic differentiation was assessed after 7 days using ALP staining and after 21 days using ARS staining. As shown in Figure S19A,B, both free OGP and OGP released from the nanohybrid in the presence of MMP9 significantly enhanced ALP activity, compared to the negative control and the nanohybrid group without MMP9. Notably, no statistically significant difference was observed between the free OGP and released OGP group (CaO_2_@CuMOF@HAP + 10 µg/mL MMP9), indicating that the released OGP retains full bioactivity. Consistent results were obtained from ARS staining after 21 days of induction (Figure S19C,D). Together, these findings confirm that OGP released from the CaO_2_@CuMOF@HAP nanohybrid upon MMP9 cleavage maintains its osteogenic bioactivity. Furthermore, as an essential indicator for new bone formation, the production of collagen fibers was evaluated through IF staining of COL1A1. As a result, CaO_2_@CuMOF@HAP significantly upregulated the expression of COL1A1, which was supported by the enhanced red fluorescence signals localized in the cytoplasmic compartment (Figure 4H).

To further verify the role of CaO_2_@CuMOF@HAP in promoting bone regeneration, the expression of key osteogenesis‐related genes, including runt‐related transcription factor 2 (*Runx2*), osterix (*Osx*), collagen type I alpha 1 chain (*Col1a1*), bone sialoprotein (*BSP*), osteopontin (*OPN*), and osteocalcin (*OCN*), was evaluated through quantitative real‐time reverse transcription polymerase chain reaction (qRT‐PCR). After 7 days of osteogenic induction, CaO_2_@CuMOF@HAP significantly upregulated the expression of *Runx2* (∼ 2.7‐fold), *Osx* (∼ 5.7‐fold), *Col1a1* (∼ 4.1‐fold), and *OCN* (∼ 4.9‐fold) compared to those in the control group (Figure 4I). *BSP* and *OPN* exhibited even more pronounced increases by at least eightfold in CaO_2_@CuMOF@HAP‐treated cells. Such a change tendency was similarly observed at 14 days postinduction. Collectively, these findings indicated that the hybrid nanoformulation of CaO_2_@CuMOF@HAP effectively promotes the osteogenic differentiation of MC3T3 cells by initiating osteogenic differentiation, regulating osteoblast maturation, stimulating bone matrix synthesis and promoting mineralization.

To further elucidate the osteogenic mechanism mediated by CaO_2_@CuMOF@HAP, we conducted western blot analysis to evaluate the expression of key proteins involved in osteogenic differentiation. Specifically, after coincubating MC3T3 cells with different formulations for 14 days, the protein levels of Runx2 and OCN were assessed. As shown in Figure S20, Runx2 expression was significantly upregulated by the treatment of CaO_2_@CuMOF@HAP. Given that Runx2 serves as a convergence point for multiple osteogenic cascades, this result suggests that the OGP likely initiates osteogenic differentiation by activating the canonical pathways. Regarding OCN expression, all CaO_2_‐containing formulations (CaO_2_, CaO_2_@CuMOF, CaO_2_@CuMOF@HA, and CaO_2_@CuMOF@HAP) exhibited significantly higher OCN levels compared to the control group. This indicates that the CaO_2_ component may contribute to late‐stage osteogenic maturation, potentially through the release of calcium ions, thereby synergizing with OGP to promote overall osteogenic differentiation.

### 2.5 In Vivo Antitumor Effects and Mechanism Evaluation

**FIGURE 5:**
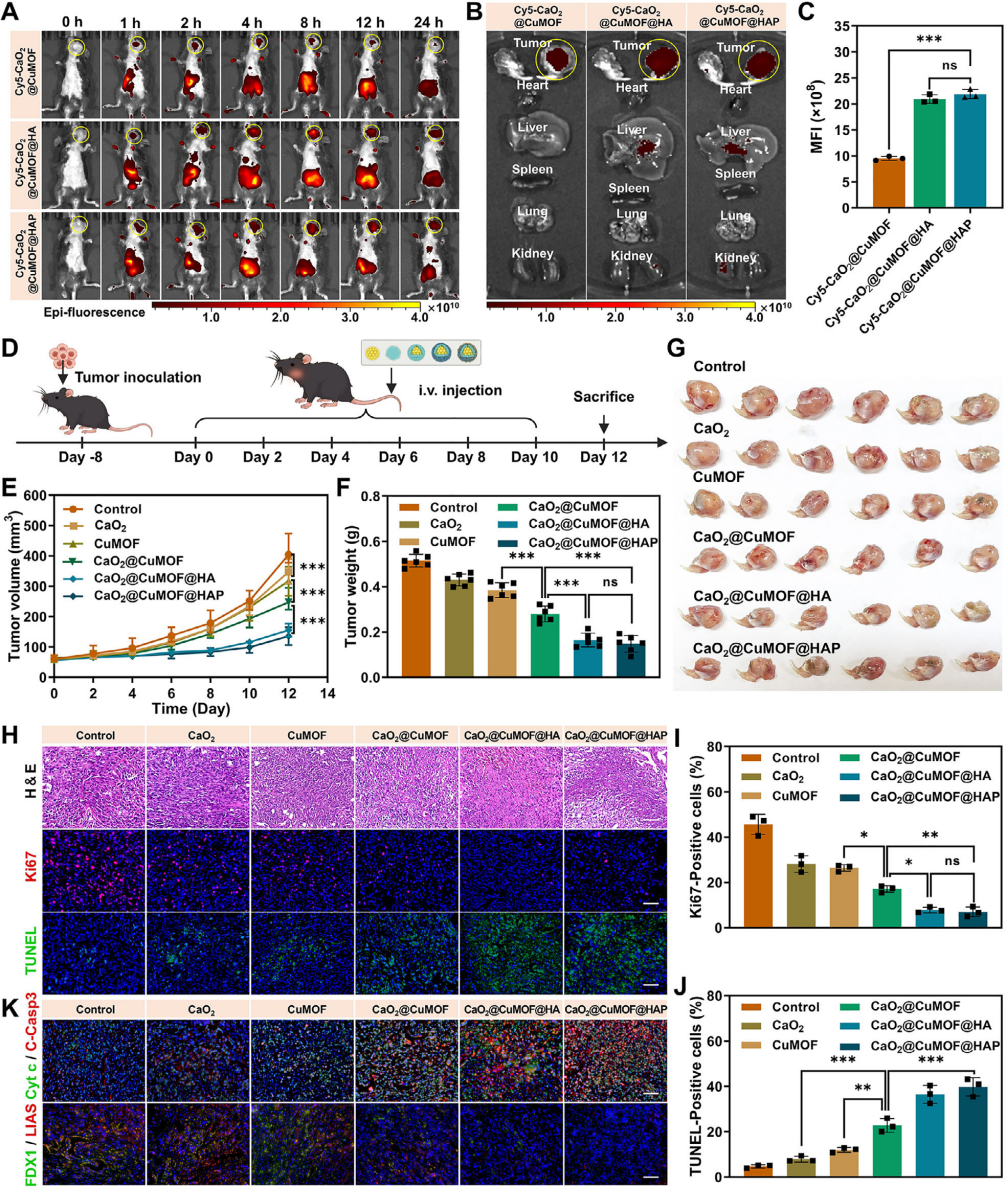
In vivo antitumor effects and mechanism evaluation. (A) In vivo imaging of tumor targetability on MOC2 tumor‐bearing mice intravenously injected with Cy5‐CaO_2_@CuMOF, Cy5‐CaO_2_@CuMOF@HA, and Cy5‐CaO_2_@CuMOF@HAP at different time points. Yellow circle: tumor site. (B) Ex vivo imaging of isolated organs and tumors at 24 h postinjection. (C) The mean fluorescence intensity (MFI) of main organs and tumors after 24 h administration. Quantitative analysis of fluorescence signals was performed using the IVIS system by delineating standardized regions of interest (ROIs) with Living Image software and calculating the average radiance. Data were performed as the mean ± SD (*n* = 3). (D) Schematic illustration of the treatment schedule in vivo. (E) Tumor growth curves. Data were performed as the mean ± SD (*n* = 6). (F) Average tumor weight. Data were performed as the mean ± SD (*n* = 6). (G) Images of excised tumor‐bearing mandibles. (H) H&E, Ki67, and TUNEL staining of the harvested tumors. Scale bar: 100 µm. (I) Quantitative analysis of Ki67‐positive cells in different groups. Data were performed as the mean ± SD (*n* = 3). (J) Quantitative analysis of TUNEL‐positive cells in different groups. Data were performed as the mean ± SD (*n* = 3). (K) IF staining of cleaved caspase‐3, Cyt c, LIAS, and FDX1 in harvested tumors with different treatments. Scale bar: 100 µm. One‐way ANOVA with Tukey's post hoc test was used for multiple comparisons in (C), (F), (I), and (J). Two‐way ANOVA with Tukey's post hoc test was used for multiple comparisons in (E). *p < 0.05, **p < 0.01, ***p < 0.001.

Inspired by its favorable physicochemical properties, high in vitro cytotoxicity against tumor cells, and excellent in vitro osteogenic effect, the systematic in vivo performance of CaO_2_@CuMOF@HAP was further evaluated in a mouse model bearing orthotopic OSCC tumors with mandibular bone invasion, which was established via orthotopic inoculation of MOC2 cells in the left buccal region of mice. Prior to performing therapeutic evaluation, an in vivo tumor targeting test was performed to confirm whether the HA‐mediated recognition of tumor cells could enable preferential accumulation of CaO_2_@CuMOF@HAP at the tumor site. The near‐infrared fluorophore cyanine5 (Cy5) was used to label the nanoparticles due to its excellent fluorescence properties, resulting in Cy5‐CaO_2_@CuMOF, Cy5‐CaO_2_@CuMOF@HA, and Cy5‐CaO_2_@CuMOF@HAP, which were intravenously injected into mice and visualized using a small animal imaging device. As displayed in Figure 5A, the HA‐contained nanoparticles (Cy5‐CaO_2_@CuMOF@HA and Cy5‐CaO_2_@CuMOF@HAP) showed progressively increased and sustained fluorescence intensity in the tumor region, peaking at 8 h postinjection and lasting for 24 h, whereas the nontargeted Cy5‐CaO_2_@CuMOF displayed minimal tumorous fluorescence signal. This confirmed that HA‐coating markedly enhanced the tumor‐specific targeting efficiency and accumulation of the targeted nanosystem at pathological tumor sites and, at the same time, prolonged nanoparticle retention. Additionally, as displayed by ex vivo fluorescence images of isolated tumors and major organs at 24 h postinjection, the HA‐modified nanosystem‐treated mice exhibited much fluorescence intensity in tumors (Figure 5B), which were 2.14‐fold relative to that of nontargeted group (Figure 5C; Figure S21), thereby further confirming the excellent tumor‐homing properties of CaO_2_@CuMOF@HAP.

Having demonstrated the superior tumor‐targeting effects of CaO_2_@CuMOF@HAP, the in vivo antitumor performance was further evaluated in the orthotopic MOC2 tumor‐bearing mouse model with mandibular bone invasion. When the tumor reached ∼ 60 mm^3^, all mice were randomly divided into six groups (*n* = 6 mice/group) including (1) control (PBS), (2) CaO_2_, (3) CuMOF, (4) CaO_2_@CuMOF, (5) CaO_2_@CuMOF@HA, and (6) CaO_2_@CuMOF@HAP. The nanoformulations were administered every 2 days for a total of 6 times with an injection dose corresponding to 20 mg/kg of Ca^2+^ (Figure 5D). Throughout the entire treatment period, body weight and tumor size were monitored and recorded every 2 days. Relative to the rapid tumor growth observed in negative control group, CaO_2_‐ and CuMOF‐treated groups showed slight tumor suppression, and the inhibitory effect was further reinforced by the treatment of CaO_2_@CuMOF, as a consequence of the synergistic killing effects of Ca^2+^ and Cu^2+^ (Figure 5E; Figure S22). Furthermore, owing to the precise and efficient delivery of therapeutic agents (e.g., Ca^2+^ and Cu^2+^) to the targeted tumor site, CaO_2_@CuMOF@HA and CaO_2_@CuMOF@HAP with the modification of targeting ligand revealed the maximum tumor suppression. The therapeutic efficacy was further reflected by the average tumor weights (Figure 5F) and corresponding images (Figure 5G) of excised tumor tissues at day 12. Notably, the CaO_2_@CuMOF@HA and CaO_2_@CuMOF@HAP‐treated groups achieved satisfactory tumor suppression with the tumor inhibition rates of approximately 68.03% ± 5.84% and 71.32% ± 7.11%, which were much higher than those in the CaO_2_ (16.46% ± 5.07%), CuMOF (25.26% ± 6.37%), and CaO_2_@CuMOF (45.60% ± 6.75%) groups.

Moreover, hematoxylin and eosin (H&E), terminal deoxynucleotidyl transferase dUTP nick end labeling (TUNEL), and Ki67 staining were conducted to verify the antitumor mechanism of CaO_2_@CuMOF@HAP. As shown in Figure 5H, H&E staining showed that severe cell damage such as noticeable cell shrinkage and nuclear dissociation was observed in both CaO_2_@CuMOF@HA‐ and CaO_2_@CuMOF@HAP‐treated groups, in contrast to the abundant tightly packed cells observed in the control group. Additionally, the treatment of CaO_2_@CuMOF@HA and CaO_2_@CuMOF@HAP significantly inhibited the proliferation of tumor cells, as evidenced by weak red fluorescence displayed in the Ki67 staining images, and the inhibition rate of cell proliferation ratio reached 37.89% and 38.68%, respectively (Figure 5I). Furthermore, the apoptosis analysis by TUNEL staining revealed that CaO_2_@CuMOF@HA and CaO_2_@CuMOF@HAP groups exhibited the highest number of apoptotic cells with ratios for 36.49% and 39.83%, while the treatment of CaO_2_, CuMOF, and CaO_2_@CuMOF induced limited apoptosis in tumor tissues (CaO_2_: 7.90%; CuMOF: 11.97%, CaO_2_@CuMOF: 22.76%) (Figure 5J). The potent antitumor activity of CaO_2_@CuMOF@HAP was also validated by IF staining, which revealed a marked increase in apoptosis biomarkers (Cyt c and cleaved caspase‐3) coupled with the significant downregulation of FDX1 and LIAS (Figure 5K), demonstrating the synergistic ion‐mediated therapeutic effect of Ca^2+^ and Cu^2+^ in triggering tumor cell death.

### 2.6 In Vivo Osteogenesis Promotion

**FIGURE 6:**
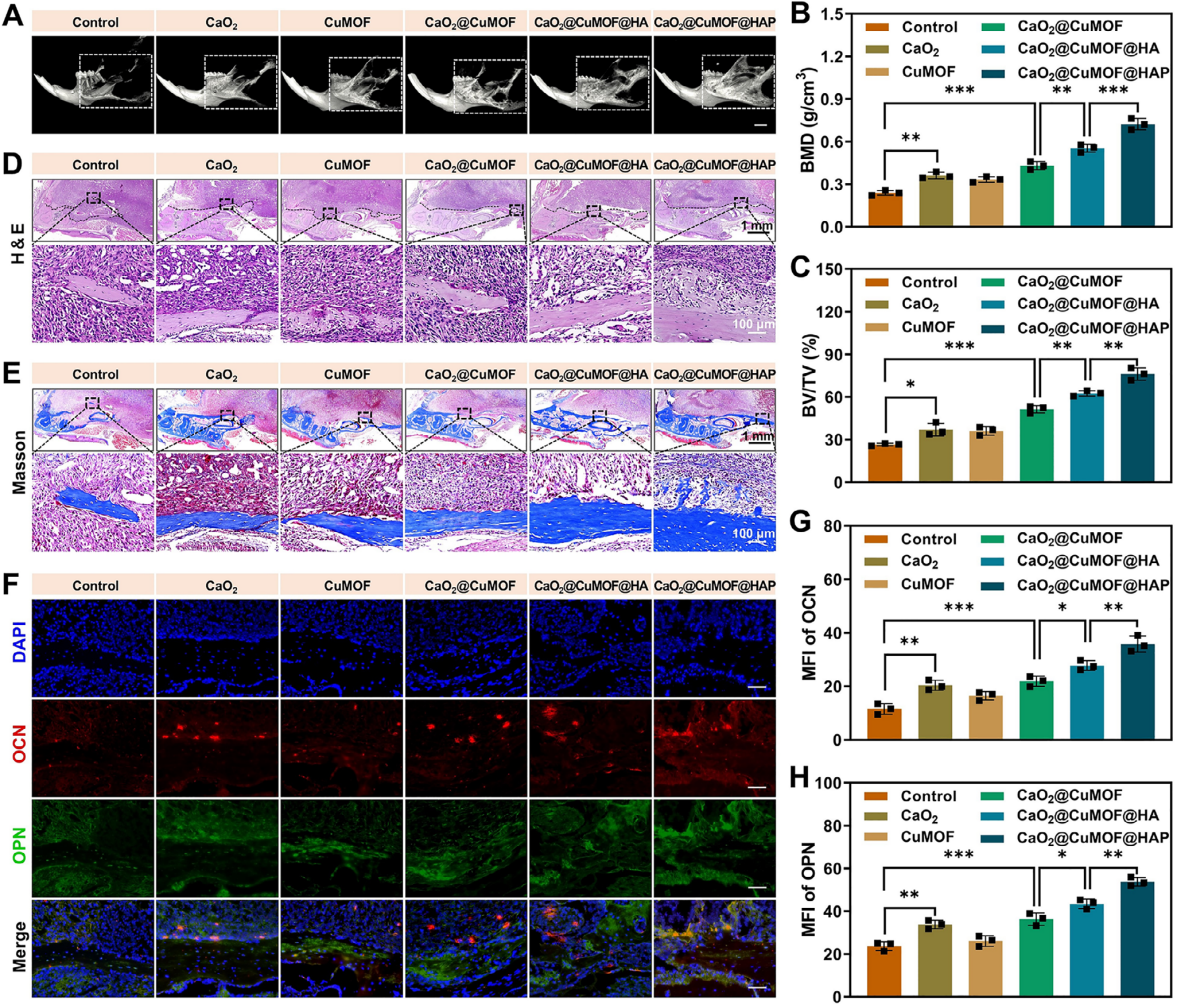
In vivo osteogenesis promotion. (A) Micro‐CT images of mandibles invaded by OSCC after different treatments. (B) Quantitative analysis of BMD obtained from micro‐CT results. (C) Quantitative analysis of BV/TV obtained from micro‐CT results. (D) H&E staining of the harvested mandibles invaded by OSCC. (E) Masson staining of the harvested mandibles invaded by OSCC. (F) IF staining of OCN (red) and OPN (green) in the harvested mandibles invaded by OSCC. Scale bar: 100 µm. (G) MFI of OCN expression in different groups. (H) MFI of OPN expression in different groups. Data were performed as the mean ± SD (*n* = 3). One‐way ANOVA with Tukey's post hoc test was used for multiple comparisons in (B), (C), (G), and (H). *p < 0.05, **p < 0.01, ***p < 0.001.

The severe bone destruction induced by aggressive tumor progression can lead to therapeutic complications, highlighting the importance of promoting osteogenesis as a critical consideration in comprehensive cancer therapy. On this basis, the osteogenic effect of CaO_2_@CuMOF@HAP was also evaluated in orthotopic MOC2 tumor‐bearing mice with mandibular bone invasion. After 12 days of treatment, the mandibles invaded by orthotopic MOC2 tumors were collected and scanned by micro‐CT to observe the bone morphology. 3D reconstruction images revealed severe bone destruction in the PBS‐treated control group, including near‐total resorption of the mandibular ramus and erosion of the alveolar bone on both buccal and lingual sides, consistent with the aggressive osteolytic nature of OSCC (Figure 6A). Following effective tumor therapy with CaO_2_@CuMOF@HA, bone destruction was significantly reduced, but the cortical bone persisted discontinuous in the affected area, manifested as through‐hole bone defects in the mandibular ramus and partial loss of buccal alveolar bone. This indicated that while the therapeutic agents (e.g., Ca^2+^ and Cu^2+^) partially inhibited osteolytic progression induced by the tumor, they failed to fully restore the architectural integrity of osseous structures. Notably, compared with CaO_2_@CuMOF@HA group, the CaO_2_@CuMOF@HAP group displayed clearly defined bone tissue boundaries and exhibited marked improvement in bone structural integrity. This could be attributed to the suppression of tumor‐induced osteolytic destruction, coupled with synergistic promotion of osteogenesis mediated by OGP. Quantitative osteomorphometric parameters, such as bone mineral density (BMD) and bone volume to total volume ratio (BV/TV), were assessed and presented in Figure 6B,C, both of which revealed the highest level of osteogenesis by CaO_2_@CuMOF@HAP treatment.

To further investigate the osteogenic capacity of CaO_2_@CuMOF@HAP, a comprehensive histological analysis, including H&E staining and Masson staining, on the harvested mandibles invaded by OSCC, was performed to directly observe the bone state. H&E staining images of the control group indicated that OSCC displayed pronounced invasiveness and destructive potential in the mandible, pathologically characterized by extensive infiltrative lesions in the mandibular and alveolar bone regions, along with extensive osseous defects (Figure 6D). Comparatively, mice treated with CaO_2_ and CaO_2_@CuMOF exhibited slight augmentation in bone tissue area, while extensive osseous defects still remained, indicating their limited promoting efficacy in bone mass accrual. Besides, after treatment with CaO_2_@CuMOF@HA, the cortical bone discontinuity persisted in the lesioned area, suggesting that the tumor‐induced osteolytic progression was partially suppressed. In marked contrast, the CaO_2_@CuMOF@HAP (with OGP modification) group demonstrated well‐defined bone tissue margins and increased cortical bone thickness, which might be attributed to the bone protective effect of OGP for promoting bone restoration. Similarly, a significantly elevated level of bone tissue in the CaO_2_@CuMOF@HAP group was observed from Masson staining images (Figure 6E), further confirming that CaO_2_@CuMOF@HAP can effectively promote osteogenesis in vivo. The above results demonstrated that CaO_2_@CuMOF@HAP could effectively promote new bone formation, thus increasing bone volume and bone density.

A favorable bone recovery is usually reflected by the upregulation of osteogenesis‐related markers. To this end, the expression of OCN and OPN was assessed by IF staining. As shown in Figure 6F, CaO_2_@CuMOF@HAP significantly upregulated the expression of both OPN and OCN proteins, demonstrating its ability to enhance osteogenic differentiation and maturation, thereby promoting osseous regeneration. Quantitative fluorescence analysis further confirmed the remarkable osteogenic potential of CaO_2_@CuMOF@HAP (Figure 6G,H), which can be attributed to its calcium‐rich composition and incorporated osteogenic promoting ligand (OGP). Overall, these results indicated that CaO_2_@CuMOF@HAP not only suppressed tumor‐induced osteolytic destruction through its robust antitumor efficacy, but also promoted osteoblast differentiation and maturation owing to the introduction of osteogenic components (e.g., Ca^2+^ and OGP), thereby enhancing bone formation and improving bone quality. More importantly, the unique combination of tumoricidal and osteogenic properties provides CaO_2_@CuMOF@HAP with distinct advantages for the comprehensive treatment of bone‐invasive OSCC, endowing this nanohybrid with great therapeutic potential for treating aggressive malignancies with severe bone invasion.

### 2.7 In Vivo Biosafety Evaluation

Ideal tumor treatment should combine good therapeutic efficacy with minimal side effects, so that physiological and biochemical metrics were recorded to evaluate the potential systemic toxicity of the designed nanosystem. In all treated groups, no obvious changes of body weight were observed throughout the entire treatment period (Figure S23). And serum biochemical analysis revealed that hepatic function markers, including aspartate aminotransferase (AST) and alanine aminotransferase (ALT), as well as renal function markers, such as blood urea nitrogen (BUN) and creatinine (CR), maintained values within reference intervals, confirming the absence of detectable hepatorenal toxicity (Figure S24). Furthermore, blood samples were obtained for complete blood count analysis, with all parameters demonstrating comparable levels relative to the control group (Figure S25). Besides, H&E staining of the major organs (heart, liver, spleen, lung, and kidney) showed no detectable pathological abnormalities, suggesting the low systemic toxicity of CaO_2_@CuMOF@HAP (Figure S26), even with a longer time evaluation (Figure S27). These findings demonstrate the excellent in vivo biocompatibility of the nanohybrid, further highlighting its promise for future clinical applications.

## 3 Conclusion

In summary, a multifunctional nanohybrid (CaO_2_@CuMOF@HAP) was successfully synthesized as a dual‐responsive delivery nanosystem for simultaneous tumor elimination and osteogenesis promotion. With the embedment of bimetallic nanoclusters (CaO_2_@CuMOF) and modification of HAP, the obtained CaO_2_@CuMOF@HAP could specifically accumulate at tumor sites to enable sequential release: (1) MMP9‐triggered OGP release in tumor extracellular space and (2) pH‐triggered decomposition of the nanohybrid in tumor cells for intracellular Cu^2+^/Ca^2+^ release. As such, tumor cells could be targetedly killed via the synergistic apoptosis/cuproptosis‐inducing effect of the dual ions (e.g., Cu^2+^ and Ca^2+^). Meanwhile, effective regeneration and repair of lesioned osseous tissue was realized by the deep‐penetrating OGP to exert osteogenesis‐promoting effect, with preserved osteoblastic viability, markedly enhanced osteoblast maturation, matrix secretion, and mineralization cascades. As evidenced in MOC2 tumor‐bearing mice with mandibular bone invasion, a significant antitumor effect was achieved and the bone destruction was effectively alleviated, demonstrating the effectiveness of CaO_2_@CuMOF@HAP for treating tumors with serious bone invasion. This versatile nanoplatform without the use of cytotoxic drugs exhibits excellent biocompatibility as well as high specificity, exhibiting strong potential for clinical translation. Within the complex bone metastatic microenvironment, other key bone cells, such as osteoclasts and osteocytes, have not been sufficiently explored. Future efforts focusing on systematically dissecting the key molecular targets and regulatory pathways within this microenvironment will better support the precise design and translational application of targeted intervention strategies.

## 4 Experimental Section

### 4.1 Materials

Hyaluronic acid (30–45 kDa) was obtained from Shanghai Macklin Biochemical Technology Co., Ltd. (China). Peptides containing the sequences of GPLGLPGYGFGG and GPLGLPGK(FITC)YGFGG were purchased from GL Biochem Co., Ltd. (China), and their corresponding characterizations were shown in the Supporting Information (Figures S28‐S31). Polyvinylpyrrolidone (PVP), EDC, NHS, copper nitrate trihydrate (Cu(NO_3_)_2_·3H_2_O), calcium chloride (CaCl_2_), and trimesic acid (BTC) were obtained from Shanghai Aladdin Biochemical Technology Co., Ltd. (China). Cyanine5 (Cy5), rhodamine B, dexamethasone, ascorbic acid, β‐glycerophosphate disodium salt hydrate, and hexadecylpyridinium chloride were obtained from Sigma‐Aldrich Co., LLC (USA). Methanol, ethanol, NH_3_·H_2_O, and H_2_O_2_ were obtained from Sinopharm Chemical Reagent Co., Ltd. (China). Cell Counting Kit‐8, Fluo‐4 acetoxymethyl ester (Fluo‐4 AM) probe, dichlorodihydrofluorescein diacetate (DCFH‐DA) probe, 5,5′,6,6′‐tetrachloro‐1,1′,3,3′‐tetraethylbenzimidazolocarbocyanine iodide (JC‐1) probe, Hoechst 33342, 4′,6‐diamidino‐2′‐phenylindole (DAPI) staining solution, GSH and GSSG assay kit, ATP assay kit, Calcein/PI live/dead assay kit, 5‐bromo‐4‐chloro‐3‐indolyl phosphate/nitrotetrazolium blue chloride (BCIP/NBT) alkaline phosphatase color development kit, alkaline phosphatase assay kit, Alizarin Red S staining kit for osteogenesis, one step TUNEL apoptosis assay kit, Alexa Fluor 594 conjugated goat anti‐rabbit IgG, and Alexa Fluor 488 conjugated goat anti‐mouse IgG were provided by Beyotime Biotechnology Co., Ltd. (China). Double‐Fluorescence immunohistochemical mouse/rabbit kit was purchased from Immunoway Biotechnology Co., Ltd. (USA). MMP9 recombinant protein, and coppersensor 1 (CS‐1) probe were purchased from MedChemExpress (USA). Ki67 rabbit pAb (Cat: GB111499), Masson staining kit, and H&E staining kit were obtained from Wuhan Servicebio Technology Co., Ltd. (China). FDX1 rabbit mAb (Cat: A20895), cytochrome c rabbit pAb (Cat: A13430) and COL1A1 rabbit pAb (Cat: A1352) were obtained from ABclonal Biotechnology Co., Ltd. (China). DLAT mouse mAb (Cat: 68303‐1‐Ig), LIAS rabbit pAb (Cat: 11577‐1‐AP), BAX mouse mAb (Cat: 60267‐1‐Ig), BCL2 rabbit pAb (Cat: 26593‐1‐AP), and cleaved caspase‐3 rabbit pAb (Cat: 25128‐1‐AP) were obtained from Proteintech Group, Inc. (China). Annexin V‐FITC/PI apoptosis kit, Calcium (Ca^2+^) colorimetric assay kit, and copper (Cu^2+^) colorimetric assay kit were obtained from Elabscience Biotechnology Co., Ltd. (China). Fetal bovine serum (FBS, Cat: 10099141C), PBS (Cat: C10010500BT), alpha‐Minimum Essential Medium (α‐MEM, Cat: 12571500BT), Dulbecco's Modified Eagle's Medium (DMEM, Cat: 11995500BT), penicillin–streptomycin (P/S, Cat: 15140122), and trypsin (Cat: 25200056) were purchased from Gibco Invitrogen Corp (USA). Ham's F12 nutrient medium (F12, Cat:SH30026.01) and Iscove's Modified Dulbecco's Medium (IMDM, Cat: SH30228.01) were purchased from Thermo Scientific HyClone (USA).

### 4.2 Apparatus

The ^1^H NMR spectra were obtained by a Bruker AVANCE NEO 600 spectrometer (600 MHz). The morphologies of nanoparticles were observed using TEM (HT7700, Japan). TEM (JEM‐F200, Japan) integrated with an EDS detector was used for elemental mapping of CaO_2_@CuMOF@HAP. X‐ray diffraction patterns were detected by an X‐ray diffractometer (Smartlab SE, Japan). XPS analysis was performed using an ESCALAB 250XI spectrometer (Thermo Scientific, USA). A Zetasizer Nano ZS (Malvern Instruments Ltd, UK) was used to determine hydrodynamic diameters and zeta potentials of nanoparticles. Flow cytometry analysis was conducted by a flow cytometer (CytoFLEX S, Beckman, USA). Fluorescence images were obtained by CLSM (Olympus FV2000, Japan). Ca^2+^ content, Cu^2+^ content, cell viability, ATP content, and GSH content were recorded by a microplate reader (Bio‐Rad, Microplate Reader 550).

### 4.3 Cell Culture

The mouse oral cancer 2 cell line (MOC2, Cat: EWL002‐FP, RRID: CVCL_ZD33) was acquired from Kerafast, Inc. (USA), and was cultured in a 5% CO_2_, 37°C incubator using IMDM/F12 at a 2:1 mixture with 5% FBS, 1% P/S, 1% amphotericin, 5 ng/mL epidermal growth factor, 400 ng/mL hydrocortisone, and 5 µg/mL insulin. Murine embryonic fibroblast cell line (NIH/3T3, Cat: CL‐0171, RRID:CVCL_0594) purchased from Wuhan Pricella Biotechnology Co., Ltd. (China) was provided by the School of Stomatology, Wuhan University (China), and was cultured in a 5% CO_2_, 37°C incubator using DMEM with 10% FBS and 1% P/S. Mouse embryonic calvaria‐derived osteoprogenitor cell line (MC3T3, Cat: CL‐0378, RRID:CVCL_5437) was purchased from Wuhan Pricella Biotechnology Co., Ltd. (China), and was cultured in a 5% CO_2_, 37°C incubator using α‐MEM with 10% FBS and 1% P/S. The cell lines were free of mycoplasma contamination for the described experiments.

### 4.4 Synthesis of Peptide‐Modified Hyaluronic Acid (HAP)

HA (37.9 mg, 30–45 kDa) was dissolved in PBS (5 mL, pH 6.0) within a 25 mL single‐neck round‐bottom flask. EDC (23.00 mg) and NHS (13.81 mg) were dissolved separately in 1 mL of PBS (pH 6.0) and subsequently introduced into the HA solution. Having reacted at room temperature (25°C) for 2 h, the peptide GPLGLPGYGFGG (54.55 mg) was dissolved in distilled water and then added to the mixture. After stirring for another 18–24 h, the mixture was dialyzed for 3 days and lyophilized. The product was stored at −80°C.

### 4.5 Preparation of CaO_2_, CaO_2_@CuMOF, CaO_2_@CuMOF@HA, and CaO_2_@CuMOF@HAP

CaO_2_ nanoparticles were synthesized following a previously reported protocol with slight modifications [60]. Briefly, 2 g of CaCl_2_ and 5 g of PVP were dissolved in 100 mL of ethanol under ultrasonication. Subsequently, 20 mL of NH_3_·H_2_O was added while stirring. Then, 2 mL of H_2_O_2_ solution was added dropwise under vigorous stirring conditions using a syringe. After reacting for 1 h, the product was collected via centrifugation at 12 000 rpm for 10 min, and washed with ethanol for obtaining CaO_2_ nanoparticles. Thereafter, 1 mL of the as‐prepared CaO_2_ (20 mg/mL) suspension was mixed with 7 mL of ethanol and 1 mL of BTC solution (10 mg/mL) for 1 h, and then 1 mL of Cu(NO_3_)_2_ solution (10 mg/mL) was added under vigorous stirring. After reacting at room temperature for another 1 h, the resulting mixture was subjected to centrifugation and washing procedures to isolate CaO_2_@CuMOF nanoparticles.

The HA‐coated nanoparticles (CaO_2_@CuMOF@HA and CaO_2_@CuMOF@HAP) were prepared by mixing CaO_2_@CuMOF nanoparticles with HA or HAP at a mass ratio of 2:1 under continuous stirring for 24 h, followed by centrifugation and repeated washing to yield the resultant nanoparticles. CaO_2_@CuMOF@HAP was dispersed in PBS buffer (pH 7.4) and stored at 4°C for use within one month.

### 4.6 MMP9‐Triggered Peptide Release

To explore the MMP9‐triggered release performance of OGP from CaO_2_@CuMOF@HAP nanoparticles over time, the FITC‐modified peptide (GPLGLPGK(FITC)YGFGG) was conjugated to HA to prepare FITC‐HAP, and the substitution degree of FITC‐modified peptide onto HA was 13.85% ± 1.13% detected by fluorescence spectrometer (RF‐530/PC, Shimadzu). Then CaO_2_@CuMOF@FITC‐HAP nanoparticles were prepared following similar procedures as CaO_2_@CuMOF@HAP nanoparticles. 1 mL of as‐prepared CaO_2_@CuMOF@FITC‐HAP nanoparticles suspension (10 mg/mL) with different concentrations of MMP9 protein: 0, 5, and 10 µg/mL was sealed in a dialysis bag and immersed in 30 mL of PBS solution. And all of the samples were shaken gently at 200 rpm at 37°C. 1 mL of the dialysate containing free peptide was collected and replaced with an equal volume of fresh PBS solution at the predetermined time points (0, 0.5, 1, 2, 4, 8, 12, 24, 36, and 48 h). The concentration of released peptide was measured by fluorescence spectrometer.

### 4.7 pH‐Controlled Ca^2+^ and Cu^2+^ Release

To explore the pH‐controlled release performance of Ca^2+^ and Cu^2+^ from CaO_2_@CuMOF@HAP over time, 1 mL of as‐prepared CaO_2_@CuMOF@HAP nanoparticles suspension (10 mg/mL) was sealed in a dialysis bag and immersed in 30 mL of PBS solutions with different pH conditions: 5.5, 6.5, and 7.4. All of the samples were shaken gently at 200 rpm at 37°C. 1 mL of the dialysate containing Ca^2+^ and Cu^2+^ was collected and replaced with an equal volume of fresh corresponding PBS solution at the predetermined time points (0, 0.5, 1, 2, 4, 8, 12, 24, 36, and 48 h). The concentrations of released Ca^2+^ and Cu^2+^ were quantified by a microplate reader with calcium (Ca^2+^) colorimetric assay kit and copper (Cu^2+^) colorimetric assay kit.

### 4.8 Cellular Uptake In Vitro

To investigate the cellular uptake efficiency, we prepared rhodamine B‐labeled CaO_2_@CuMOF, CaO_2_@CuMOF@HA, and CaO_2_@CuMOF@HAP nanoparticles, and incubated them with NIH3T3 cells and MOC2 cells for 4 h, respectively. After removing the culture media, fixing with 4% paraformaldehyde, and staining nuclei with DAPI, fluorescence images were taken by CLSM with laser at the Ex of 559 nm. For flow cytometry analysis, cells incubated with rhodamine B‐labeled CaO_2_@CuMOF, CaO_2_@CuMOF@HA, and CaO_2_@CuMOF@HAP nanoparticles were detached from the plates using trypsin (depleted of disodium ethylenediaminetetraacetate (EDTA)) and subsequently collected by centrifugation. After washing with PBS, the intracellular fluorescence intensity was analyzed by flow cytometry.

### 4.9 Cell Viability Assay In Vitro

Cells were seeded in 96‐well plates and cultured until the cell density reached approximately 80%. After incubation with PBS, CaO_2_, CuMOF, CaO_2_@CuMOF, CaO_2_@CuMOF@HA, and CaO_2_@CuMOF@HAP nanoparticles at various concentrations (equivalent Ca^2+^ concentrations: 0, 5, 10, 20, 30, 40, and 50 µg/mL) for 24 h, respectively, the cells were further incubated with 10 µL of CCK‐8 solutions for an additional 1 h. The absorbance at 450 nm was measured using a microplate reader. Cell viability was calculated using the following formula: [(OD450_Sample_—OD450_Blank_)/(OD450_Control_—OD450_Blank_)]×100%.

### 4.10 Apoptosis/Necrosis Analysis

MOC2 cells incubated with PBS, CaO_2_, CuMOF, CaO_2_@CuMOF, CaO_2_@CuMOF@HA, and CaO_2_@CuMOF@HAP (equivalent Ca^2+^ concentrations: 20 µg/mL) were detached from the plates using trypsin (depleted of EDTA) and subsequently collected by centrifugation. After washing with PBS, the collected cells were incubated in 500 µL of binding buffer containing 5 µL of Annexin V and 5 µL of PI at room temperature in the dark for 20 min. Then the cells were subjected to detection via flow cytometry.

### 4.11 Wound Healing Assay

MOC2 cells were seeded in 6‐well plates and cultured until the cell density reached approximately 80%. The straight scratches among cultured MOC2 cells were created using a pipette tip. The media were removed and then substituted with fresh media supplemented with PBS, CaO_2_, CuMOF, CaO_2_@CuMOF, CaO_2_@CuMOF@HA, and CaO_2_@CuMOF@HAP. The images of cell migration were taken at the predetermined time points (0, 12, and 24 h). The captured images were analyzed using ImageJ software with a standardized protocol. The wound area at each time point was measured by manually delineating the cell‐free area using the polygon selection tool. The recovery ratio was calculated using the following formula: Recovery ratio (%) = [(Area_0h_—Area_nh_)/Area_0h_]×100% (where Area_0h_ is the initial wound area at 0 h, and Area_nh_ is the remaining wound area after n hours).

### 4.12 In Vitro Assessment of Antitumor Mechanism

MOC2 cells were treated with PBS, CaO_2_, CuMOF, CaO_2_@CuMOF, CaO_2_@CuMOF@HA, and CaO_2_@CuMOF@HAP for 6 h. Thereafter, cells were analyzed using probes, including JC‐1 probe (for mitochondrial membrane potential assay), DCFH‐DA (10 µm, 30 min) (for intracellular ROS detection), Fluo‐4 AM (5 µm, 30 min) (for Ca^2+^ detection), Ca^2+^ colorimetric assay kit, Cu^2+^ colorimetric assay kit, CS‐1 probe (for Cu^+^ level detection), ATP assay kit, and Cu‐ATPase activity assay kit, for CLSM observation as well as flow cytometry analysis.

### 4.13 Western Blot Analysis

Cells were treated with PBS, CaO_2_, CuMOF, CaO_2_@CuMOF, CaO_2_@CuMOF@HA, and CaO_2_@CuMOF@HAP, respectively. After removing the culture medium and washing with PBS, the cells were scraped off, and lysed with protein lysis buffer to collect proteins. Protein samples were separated by SDS‐PAGE and transferred onto PVDF membranes. Following a 30 min blocking step with a rapid western blocking solution at room temperature, the membranes were incubated with primary antibodies overnight at 4°C. After washing with TBST buffer, the membranes were incubated with HRP‐conjugated secondary antibodies for an additional 1 h. The immunoreactive bands were visualized using a chemiluminescence detection system and quantitatively analyzed with ImageJ software.

### 4.14 Immunofluorescence Staining

Cells treated with PBS, CaO_2_, CuMOF, CaO_2_@CuMOF, CaO_2_@CuMOF@HA, and CaO_2_@CuMOF@HAP were sequentially rinsed with PBS, fixed with 4% paraformaldehyde, incubated with primary antibody at 4°C overnight, exposed to fluorescent secondary antibody at room temperature for 1 h, stained with Actin‐Tracker Green‐488 for 30 min, counterstained with DAPI for 15 min, and visualized by CLSM.

### 4.15 Live/Dead Staining

MC3T3 cells treated with PBS, CaO_2_, CuMOF, CaO_2_@CuMOF, CaO_2_@CuMOF@HA, and CaO_2_@CuMOF@HAP for 3 days were sequentially rinsed with PBS, and stained with a Calcein/PI live/dead assay kit. Fluorescence images were captured using an inverted fluorescence microscope (Olympus, Japan).

### 4.16 ALP Staining and Activity Detection

MC3T3 cells were seeded into 24‐well plates and cultured until the cell density reached approximately 60%. The culture medium was then replaced with osteogenic supplement‐containing medium, and the cells were treated with PBS, CaO_2_, CuMOF, CaO_2_@CuMOF, CaO_2_@CuMOF@HA, and CaO_2_@CuMOF@HAP (equivalent Ca^2+^ concentration: 20 µg/mL) for 7 days, respectively. The cells were sequentially washed with PBS, fixed with 4% paraformaldehyde, followed by ALP staining protocol. ALP activity was detected using an alkaline phosphatase assay kit, along with assessment of total protein content using a BCA protein assay kit.

### 4.17 ARS Staining and Quantitative Analysis

MC3T3 cells were seeded into 6‐well plates and cultured until the cell density reached approximately 60%. The culture medium was then replaced with osteogenic supplement‐containing medium for further culture and treated with PBS, CaO_2_, CuMOF, CaO_2_@CuMOF, CaO_2_@CuMOF@HA, and CaO_2_@CuMOF@HAP (equivalent Ca^2+^ concentrations: 20 µg/mL) for 21 days, respectively. The cells were sequentially washed with PBS, fixed with 4% paraformaldehyde, followed by ARS staining protocol. In order to quantify calcium mineralization, the stained MC3T3 cells were incubated with 10% hexadecylpyridinium chloride solution at room temperature for 1 h, and absorbance values were quantified via spectrophotometric detection at 540 nm using a microplate reader.

### 4.18 Quantitative Real‐Time Reverse Transcription Polymerase Chain Reaction (qRT‐PCR)

MC3T3 cells were seeded into 6‐well plates and cultured until the cell density reached approximately 60%. The culture medium was then replaced with osteogenic supplement‐containing medium for further culture and the cells were treated with PBS, CaO_2_, CuMOF, CaO_2_@CuMOF, CaO_2_@CuMOF@HA, and CaO_2_@CuMOF@HAP (equivalent Ca^2+^ concentrations: 20 µg/mL) for 7 days and 14 days, respectively. The cells were sequentially washed with PBS, lysed with Trizol reagent to extract the total RNA. QRT‐PCR was performed using a Quant Studio 6 (Applied Biosystems) with ChamQ SYBR qPCR Master Mix in triplicate. The primer sequences are listed in Table S1. The relative fold changes of the *Runx2*, *Osx*, *Col1a1*, *BSP*, *OPN*, and *OCN* expression were calculated and normalized based on the 2^−ΔΔCt^ method.

### 4.19 Animals and Tumor Model

Six‐week‐old female C57BL/6 mice were procured from the Cyagen Institute and used to establish mandibular bone invasion models. All animal experiments were conducted in accordance with the guidelines established by the Institutional Animal Care and Use Committee of the Animal Experiment Center of Wuhan University (Wuhan, P. R. China) and were performed following the guidelines of the Ethics Committee of School of Stomatology, Wuhan University (Project Number: S07924010H). To establish the experimental model of OSCC invading the mandibular bone, 2×10^5^ of MOC2 cells suspended in 50 µL of PBS solution were injected into the left buccal region of mice.

### 4.20 In Vivo Tumor Targeting and Biodistribution

Cy5‐labeled CaO_2_@CuMOF, CaO_2_@CuMOF@HA, and CaO_2_@CuMOF@HAP nanoparticles (Cy5 concentration: 1 mg/mL) were synthesized and intravenously injected to evaluate the tumor targeting efficacy in vivo. After being anesthetized, the mice were subjected to longitudinal imaging in vivo using an IVIS imaging system (IVIS Spectrum, PerkinElmer, excitation: 650 nm, emission: 670 nm) at predetermined time points (0, 1, 2, 4, 8, 12, and 24 h). Following 24‐h observation, the mice were sacrificed to collect tumor tissues and major organs (heart, liver, spleen, lung, kidney) for ex vivo biodistribution analysis. Quantitative analysis of fluorescence signals in ex vivo tumor tissues and major organs (heart, liver, spleen, lung, and kidney) was performed using the IVIS system by delineating standardized regions of interest with Living Image software and calculating the average radiance.

### 4.21 In Vivo Evaluation of Antitumor Effects

When the tumor volume reached ∼ 60 mm^3^, the mice bearing orthotopic OSCC tumors were randomly and blindly divided into 6 groups (*n* = 6 biologically independent samples per group): (1) control (PBS), (2) CaO_2_, (3) CuMOF, (4) CaO_2_@CuMOF, (5) CaO_2_@CuMOF@HA, and (6) CaO_2_@CuMOF@HAP. The therapeutic agents were administered every 2 days for a total of 6 intravenous injections, at a dose equivalent to 20 mg/kg of Ca^2+^ (corresponding to 3.71 mg/kg of Cu^2+^). Body weight and tumor size were monitored at two‐day intervals, with tumor volume calculated using the formula: (tumor length×tumor width^2^)/2. On day 12 post‐treatment, all mice were euthanized, and the excised tumors and major organs (heart, liver, spleen, lung, and kidney) were collected for H&E staining, TUNEL staining, Ki67 staining, and IF staining. Furthermore, whole blood samples were also collected to conduct comprehensive hematological profiling (complete blood count) and serum biochemical analysis (ALT, AST, BUN, CR levels).

### 4.22 In Vivo Evaluation of Osteogenesis Effects

The mandibles of different groups were collected and scanned using a SkyScan 1276 micro‐CT system (Bruker) at 60 kV and 6 µm resolution to observe bone defects. 3D models were reconstructed with NRecon software and visualized with CTVox. Quantification of BMD and BV/TV was performed using CTAn software. For histological studies, the mandible samples were decalcified in EDTA solution for 1 month, dehydrated, embedded in paraffin, and sectioned into 4 µm slices. H&E staining and Masson staining were carried out in accordance with the manufacturer's instructions. For IF staining, after slices were dewaxed, rehydrated, subjected to antigen retrieval, and blocked, they were incubated with primary antibodies overnight at 4°C, followed by incubation with Cy3‐ or FITC‐conjugated secondary antibodies at room temperature for 1 h. After sealing with an antifluorescence quenching agent, images were captured using CLSM.

### 4.23 In Vivo Evaluation of Long‐Term Biosafety

PBS and the therapeutic agent CaO_2_@CuMOF@HAP were administered every 2 days for a total of 13 intravenous injections, at a dose equivalent to 20 mg/kg of Ca^2+^ (corresponding to 3.71 mg/kg of Cu^2+^). On day 28 postadministration, all mice were euthanized, and major organs (heart, liver, spleen, lung, and kidney) were collected for H&E staining to evaluate the long‐term biosafety.

### 4.24 Statistical Analysis

The GraphPad Prism software (version 9.5.0) was used for the statistical analysis. All data were expressed as mean ± standard deviation (SD). Multiple comparisons were performed using one‐way or two‐way analysis of variance (ANOVA) with Tukey's multiple comparison test. A probability value of *p* < 0.05 was considered statistically significant.

## Conflicts of Interest

The authors declare no conflicts of interest.

## Supporting information




**Supporting File**: advs74864‐sup‐0001‐SuppMat.docx.

## Data Availability

The data that support the findings of this study are available from the corresponding author upon reasonable request.

## References

[1] M. Tsukasaki , “Dive Into the Bone: New Insights Into Molecular Mechanisms of Cancer Bone Invasion,” Journal of Bone and Mineral Research 40, no. 7 (2025): 827–833, 10.1093/jbmr/zjaf054. 40247712

[2] P. Clézardin , R. Coleman , M. Puppo , et al., “Bone Metastasis: Mechanisms, Therapies, and Biomarkers,” Physiological Reviews 101, no. 3 (2021): 797–855, 10.1152/physrev.00012.2019. 33356915

[3] R. E. Coleman , P. I. Croucher , A. R. Padhani , et al., “Bone Metastases,” Nature Reviews Disease Primers 6, no. 1 (2020): 83, 10.1038/s41572-020-00216-3. 33060614

[4] F. Bray , M. Laversanne , H. Sung , et al., “Global Cancer Statistics 2022: GLOBOCAN Estimates of Incidence and Mortality Worldwide for 36 Cancers in 185 Countries,” CA‐A Cancer Journal for Clinicians 74, no. 3 (2024): 229–263, 10.3322/caac.21834. 38572751

[5] Y. Tan , Z. Wang , M. Xu , et al., “Oral Squamous Cell Carcinomas: State of the Field and Emerging Directions,” International Journal of Oral Science 15, no. 1 (2023): 44, 10.1038/s41368-023-00249-w. 37736748 PMC10517027

[6] T. Tada , M. Shin , H. Fukushima , et al., “Oral Squamous Cell Carcinoma Cells Modulate Osteoclast Function by RANKL‐Dependent and ‐Independent Mechanisms,” Cancer Letters 274, no. 1 (2009): 126–131, 10.1016/j.canlet.2008.09.015. 18930344

[7] L. E. Yue , K. F. Sharif , J. R. Sims , et al., “Oral Squamous Carcinoma: Aggressive Tumor Pattern of Invasion Predicts Direct Mandible Invasion,” Head Neck 42, no. 11 (2020): 3171–3178, 10.1002/hed.26360. 32710523

[8] H. Fan , Z. Xu , K. Yao , et al., “Osteoclast Cancer Cell Metabolic Cross‐Talk Confers Parp Inhibitor Resistance in Bone Metastatic Breast Cancer,” Cancer Research 84, no. 3 (2024): 449–467, 10.1158/0008-5472.CAN-23-1443. 38038966

[9] S. Jiao , S. K. Subudhi , A. Aparicio , et al., “Differences in Tumor Microenvironment Dictate T Helper Lineage Polarization and Response to Immune Checkpoint Therapy,” Cell 179, no. 5 (2019): 1177–1190.e13, 10.1016/j.cell.2019.10.029. 31730856

[10] A. Ebrahimi , R. Murali , K. Gao , M. S. Elliott , and J. R. Clark , “The Prognostic and Staging Implications of Bone Invasion in Oral Squamous Cell Carcinoma,” Cancer 117, no. 19 (2011): 4460–4467, 10.1002/cncr.26032. 21437887

[11] N. Zhu , H. Ni , S. Guo , Y.‐Q. Shen , and Q. Chen , “Bone Complications of Cancer Treatment,” Cancer Treatment Reviews 130 (2024): 102828, 10.1016/j.ctrv.2024.102828. 39270364

[12] J. Bai , Y. Wang , J. Wang , J. Zhai , F. He , and G. Zhu , “Irradiation‐Induced Senescence of Bone Marrow Mesenchymal Stem Cells Aggravates Osteogenic Differentiation Dysfunction via Paracrine Signaling,” American Journal of Physiology‐Cell Physiology 318, no. 5 (2020): C1005–C1017, 10.1152/ajpcell.00520.2019. 32233952

[13] A. M. C. Lee , J. M. Bowen , Y.‐W. Su , et al., “Individual or Combination Treatments With Lapatinib and Paclitaxel Cause Potential Bone Loss and Bone Marrow Adiposity in Rats,” Journal of Cellular Biochemistry 120, no. 3 (2019): 4180–4191, 10.1002/jcb.27705. 30260048

[14] H.‐J. Park , S.‐Y. Yoon , J.‐N. Park , J.‐H. Suh , and H.‐S. Choi , “Doxorubicin Induces Bone Loss by Increasing Autophagy Through a Mitochondrial ROS/TRPML1/TFEB Axis in Osteoclasts,” Antioxidants 11, no. 8 (2022): 1476, 10.3390/antiox11081476. 36009195 PMC9404930

[15] Y. Opoku‐Damoah , R. Wang , J. Zhou , and Y. Ding , “Versatile Nanosystem‐Based Cancer Theranostics: Design Inspiration and Predetermined Routing,” Theranostics 6, no. 7 (2016): 986–1003, 10.7150/thno.14860. 27217832 PMC4876623

[16] L. Sun , H. Liu , Y. Ye , et al., “Smart Nanoparticles for Cancer Therapy,” Signal Transduction and Targeted Therapy 8, no. 1 (2023): 418, 10.1038/s41392-023-01642-x. 37919282 PMC10622502

[17] Z. Zhang , C. Ding , T. Sun , L. Wang , and C. Chen , “Tumor Therapy Strategies Based on Microenvironment‐Specific Responsive Nanomaterials,” Advanced Healthcare Materials 12 (2023): 2300153, 10.1002/adhm.202300153. 36933000

[18] H. Li , Y. Feng , Q. Luo , et al., “Stimuli‐Activatable Nanomedicine Meets Cancer Theranostics,” Theranostics 13, no. 15 (2023): 5386–5417, 10.7150/thno.87854. 37908735 PMC10614691

[19] H. Cai , P. Tan , X. Chen , et al., “Stimuli‐Sensitive Linear–Dendritic Block Copolymer–Drug Prodrug as a Nanoplatform for Tumor Combination Therapy,” Advanced Materials 34, no. 8 (2022): 2108049, 10.1002/adma.202108049. 34875724

[20] L. Lin , Z. Fang , G. Liu , et al., “Prodrug‐Based Combinational Nanomedicine Remodels Lipid Metabolism for Reinforced Ferroptosis and Immune Activation,” Acta Pharmaceutica Sinica B 15, no. 5 (2025): 2746–2763, 10.1016/j.apsb.2025.03.016. 40487644 PMC12145006

[21] Y. Shi , R. van der Meel , X. Chen , and T. Lammers , “The EPR Effect and Beyond: Strategies to Improve Tumor Targeting and Cancer Nanomedicine Treatment Efficacy,” Theranostics 10, no. 17 (2020): 7921–7924, 10.7150/thno.49577. 32685029 PMC7359085

[22] T. Lammers , “Nanomedicine Tumor Targeting,” Advanced Materials 36, no. 26 (2024): 2312169, 10.1002/adma.202312169. 38361435

[23] Y. Li , Y. Wang , L. Zhao , M. H. Stenzel , and Y. Jiang , “Metal Ion Interference Therapy: Metal‐Based Nanomaterial‐Mediated Mechanisms and Strategies to Boost Intracellular “Ion Overload” for Cancer Treatment,” Materials Horizons 11, no. 18 (2024): 4275–4310, 10.1039/d4mh00470a. 39007354

[24] Y. Liu , Y. Wang , S. Song , and H. Zhang , “Cancer Therapeutic Strategies Based on Metal Ions,” Chemical Science 12, no. 37 (2021): 12234–12247, 10.1039/d1sc03516a. 34603654 PMC8480331

[25] X. Sun , X. Zhou , X. Shi , et al., “Strategies for the Development of Metalloimmunotherapies,” Nature Biomedical Engineering 8, no. 9 (2024): 1073–1091, 10.1038/s41551-024-01221-7. PMC11410547 38914800

[26] S. Li , Y. Cui , H. Liu , et al., “Application of Bioactive Metal Ions in the Treatment of Bone Defects,” Journal of Materials Chemistry B 10, no. 45 (2022): 9369–9388, 10.1039/d2tb01684b. 36378123

[27] Y. Wang , H. Li , J. Lin , et al., “Engineering Nanozyme Immunomodulator With Magnetic Targeting Effect for Cascade‐Enzyodynamic and Ultrasound‐Reinforced Metallo‐Immunotherapy in Prostate Carcinoma,” Nature Communications 16, no. 1 (2025): 1876, 10.1038/s41467-025-57190-1. PMC11846840 39987131

[28] L. Zhao , F. Chang , Y. Tong , et al., “A Multifunctional Bimetallic Nanoplatform for Synergic Local Hyperthermia and Chemotherapy Targeting HER2‐Positive Breast Cancer,” Advanced Science 11, no. 16 (2024): 2308316, 10.1002/advs.202308316. 38380506 PMC11040336

[29] B. Liu , H. Zhou , L. Tan , K. T. H. Siu , and X.‐Y. Guan , “Exploring Treatment Options in Cancer: Tumor Treatment Strategies,” Signal Transduction and Targeted Therapy 9, no. 1 (2024): 175, 10.1038/s41392-024-01856-7. 39013849 PMC11252281

[30] B. A. Carneiro and W. S. El‐Deiry , “Targeting Apoptosis in Cancer Therapy,” Nature Reviews Clinical Oncology 17, no. 7 (2020): 395–417, 10.1038/s41571-020-0341-y. PMC8211386 32203277

[31] G. R. Monteith , N. Prevarskaya , and S. J. Roberts‐Thomson , “The Calcium–Cancer Signalling Nexus,” Nature Reviews Cancer 17, no. 6 (2017): 373–380, 10.1038/nrc.2017.18. 28386091

[32] S. Bai , Y. Lan , S. Fu , H. Cheng , Z. Lu , and G. Liu , “Connecting Calcium‐Based Nanomaterials and Cancer: From Diagnosis to Therapy,” Nano‐Micro Letters 14, no. 1 (2022): 145, 10.1007/s40820-022-00894-6. 35849180 PMC9294135

[33] C. Qi , J. Lin , L.‐H. Fu , and P. Huang , “Calcium‐Based Biomaterials for Diagnosis, Treatment, and Theranostics,” Chemical Society Reviews 47, no. 2 (2018): 357–403, 10.1039/c6cs00746e. 29261194

[34] Y. Kang , L. Xu , J. Dong , et al., “Calcium‐Based Nanotechnology for Cancer Therapy,” Coordination Chemistry Reviews 481, no. 15 (2023): 215050, 10.1016/j.ccr.2023.215050.

[35] C. Wang , F. Yu , X. Liu , et al., “Cancer‐Specific Therapy by Artificial Modulation of Intracellular Calcium Concentration,” Advanced Healthcare Materials 8, no. 18 (2019): 1900501, 10.1002/adhm.201900501. 31368208

[36] M. Zhang , R. Song , Y. Liu , et al., “Calcium‐Overload‐Mediated Tumor Therapy by Calcium Peroxide Nanoparticles,” Chem 5, no. 8 (2019): 2171–2182, 10.1016/j.chempr.2019.06.003.

[37] G. Luo , X. Li , J. Lin , et al., “Multifunctional Calcium–Manganese Nanomodulator Provides Antitumor Treatment and Improved Immunotherapy via Reprogramming of the Tumor Microenvironment,” ACS Nano 17, no. 16 (2023): 15449–15465, 10.1021/acsnano.3c01215. 37530575 PMC10448754

[38] Z. Liu , W. Hu , Y. Cai , et al., “Calcium Peroxide Functionalized Mesoporous Polydopamine Nanoparticles Triggered Calcium Overload for Synergistic Tumor Gas/Photothermal Therapy,” Journal of Colloid and Interface Science 690 (2025): 137332, 10.1016/j.jcis.2025.137332. 40088813

[39] W. Zheng , Y. Liu , J. Liu , et al., “Copper/Calcium Co‐Doped Carbon Dots for Targeted Cancer Therapy With Dual‐Mode Imaging and Synergistic Induction of Cuproptosis and Calcium‐Mediated Apoptosis,” Journal of Colloid and Interface Science 690 (2025): 137337, 10.1016/j.jcis.2025.137337. 40117884

[40] C. Du , X. Guo , X. Qiu , et al., “Self‐Reinforced Bimetallic Mito‐Jammer for Ca^2+^ Overload‐Mediated Cascade Mitochondrial Damage for Cancer Cuproptosis Sensitization,” Advanced Science 11, no. 15 (2024): 2306031, 10.1002/advs.202306031. 38342617 PMC11022715

[41] M. Tang , J. Ni , Z. Yue , et al., “Polyoxometalate‐Nanozyme‐Integrated Nanomotors (POMotors) for Self‐Propulsion‐Promoted Synergistic Photothermal‐Catalytic Tumor Therapy,” Angewandte Chemie, International Edition in English 63, no. 6 (2024): 202315031, 10.1002/anie.202315031. 38117015

[42] H. Gu , J. Li , P. Dai , et al., “Polyphenol Oxidase‐Like Nanozymes,” Advanced Materials 37, no. 43 (2025): 09346, 10.1002/adma.202509346. 40847695

[43] Q. Xue , R. Kang , D. J. Klionsky , D. Tang , J. Liu , and X. Chen , “Copper Metabolism in Cell Death and Autophagy,” Autophagy 19, no. 8 (2023): 2175–2195, 10.1080/15548627.2023.2200554. 37055935 PMC10351475

[44] S.‐R. Li , S.‐Y. Tao , Q. Li , C.‐Y. Hu , and Z.‐J. Sun , “Harnessing Nanomaterials for Copper‐Induced Cell Death,” Biomaterials 313 (2025): 122805, 10.1016/j.biomaterials.2024.122805. 39250865

[45] P. Tsvetkov , S. Coy , B. Petrova , et al., “Copper Induces Cell Death by Targeting Lipoylated TCA Cycle Proteins,” Science 375, no. 6586 (2022): 1254–1261, 10.1126/science.abf0529. 35298263 PMC9273333

[46] B. Liu , X. Chen , Y. Zhu , et al., “One‐Step Symbiosis of Bimetallic Peroxides Nanoparticles to Induce Ferroptosis/Cuproptosis and Activate cGAS‐STING Pathway for Enhanced Tumor Immunotherapy,” Advanced Materials 37, no. 21 (2025): 2500337, 10.1002/adma.202500337. 40181655

[47] W. Xu , A. Suo , A. J. M. Aldai , et al., “Hollow Calcium/Copper Bimetallic Amplifier for Cuproptosis/Paraptosis/Apoptosis Cancer Therapy via Cascade Reinforcement of Endoplasmic Reticulum Stress and Mitochondrial Dysfunction,” ACS Nano 18, no. 43 (2024): 30053–30068, 10.1021/acsnano.4c11455. 39412236

[48] N. Zheng , Q. Wang , Z. Cao , et al., “Multifunctional H_2_S‐Activated Metal–Organic Framework Systems for Targeted Colorectal Cancer Imaging and Synergistic Copper‐Induced Tumor Regression,” BMEMat 3, no. 4 (2025): e70029, 10.1002/bmm2.70029.

[49] Y. Guan , W. Zhang , Y. Mao , and S. Li , “Nanoparticles and Bone Microenvironment: A Comprehensive Review for Malignant Bone Tumor Diagnosis and Treatment,” Molecular Cancer 23, no. 1 (2024): 246, 10.1186/s12943-024-02161-1. 39487487 PMC11529338

[50] J. Liao , R. Han , Y. Wu , and Z. Qian , “Review of a New Bone Tumor Therapy Strategy Based on Bifunctional Biomaterials,” Bone Research 9, no. 1 (2021): 18, 10.1038/s41413-021-00139-z. 33727543 PMC7966774

[51] F. E. Freeman , P. Dosta , L. C. Shanley , et al., “Localized Nanoparticle‐Mediated Delivery of miR‐29b Normalizes the Dysregulation of Bone Homeostasis Caused by Osteosarcoma Whilst Simultaneously Inhibiting Tumor Growth,” Advanced Materials 35, no. 23 (2023): 2207877, 10.1002/adma.202207877. 36994935

[52] W. Wang , W. Kang , X. Zhang , et al., “Microenvironment‐Responsive Targeted Nanomedicine for a Collaborative Integration of Tumor Theranostics and Bone Defect Repair,” Advanced Healthcare Materials 13, no. 27 (2024): 2400715, 10.1002/adhm.202400715. 38822808

[53] Y. Zhang , Y. Liu , Z. Li , Q. Zhang , and J. Li , “Breast Cancer Bone Metastasis Therapy and Tumor‐Associated Bone Destruction Repair by Versatile Semiconducting Nanointegrators With X‐Ray Adjuvant,” Advanced Functional Materials 35, no. 2 (2025): 2412165, 10.1002/adfm.202412165.

[54] C. K. Martin , J. L. Werbeck , N. K. Thudi , et al., “Zoledronic Acid Reduces Bone Loss and Tumor Growth in an Orthotopic Xenograft Model of Osteolytic Oral Squamous Cell Carcinoma,” Cancer Research 70, no. 21 (2010): 8607–8616, 10.1158/0008-5472.CAN-10-0850. 20959474 PMC2970642

[55] S. Pigossi , M. Medeiros , S. Saska , J. Cirelli , and R. Scarel‐Caminaga , “Role of Osteogenic Growth Peptide (OGP) and OGP(10–14) in Bone Regeneration: A Review,” International Journal of Molecular Sciences 17, no. 11 (2016): 1885, 10.3390/ijms17111885. 27879684 PMC5133884

[56] Y. Qiao , X. Liu , X. Zhou , et al., “Gelatin Templated Polypeptide Co‐Cross‐Linked Hydrogel for Bone Regeneration,” Advanced Healthcare Materials 9, no. 1 (2020): 1901239, 10.1002/adhm.201901239. 31814318

[57] S. Wang , W. He , H. Wang , et al., “Hematoma‐Like Dynamic Hydrogelation through Natural Glycopeptide Molecular Recognition for Infected Bone Fracture Repair,” Bioactive Materials 30 (2023): 73–84, 10.1016/j.bioactmat.2023.07.018. 37575878 PMC10413008

[58] M. Chen , Y. Sun , Y. Hou , et al., “Constructions of ROS‐Responsive Titanium‐Hydroxyapatite Implant for Mesenchymal Stem Cell Recruitment in Peri‐Implant Space and Bone Formation in Osteoporosis Microenvironment,” Bioactive Materials 18 (2022): 56–71, 10.1016/j.bioactmat.2022.02.006. 35387165 PMC8961459

[59] M. Li , J. Bai , H. Tao , et al., “Rational Integration of Defense and Repair Synergy on PEEK Osteoimplants via Biomimetic Peptide Clicking Strategy,” Bioactive Materials 8 (2021): 309–324, 10.1016/j.bioactmat.2021.07.002. 34541403 PMC8427090

[60] S. Shen , M. Mamat , S. Zhang , et al., “Synthesis of CaO_2_ Nanocrystals and Their Spherical Aggregates With Uniform Sizes for Use as a Biodegradable Bacteriostatic Agent,” Small 15, no. 36 (2019): 1902118, 10.1002/smll.201902118. 31328882

