# Supplementary material for: A Dual‐Responsive Versatile Nanohybrid Orchestrating Tumor Elimination and Tumor‐Associated Osteolysis Restoration via Sequential Release
Source: Adv Sci (Weinh). 2026 Mar 18;13(30):e18962. doi: 10.1002/advs.202518962 (PMC13248762; doi:10.1002/advs.202518962)
Supplement: Supplementary file 1 — Supporting File: advs74864‐sup‐0001‐SuppMat.docx. [file ADVS-13-e18962-s001.docx]

Supporting Information

A Dual-Responsive Versatile Nanohybrid Orchestrating Tumor Elimination and Tumor-Associated Osteolysis Restoration via Sequential Release

*Lan Liu, Han-Zhe Liu, Zhe-Nan Liu, Tong Wang, Li-Li Yu, Qiu-Jing Li, Zi-Yi Chen, Guo-Feng Luo*, and Zheng-Jun Shang**

L. Liu, H.-Z. Liu, Z.-N. Liu, T. Wang, L.-L. Yu, Q.-J. Li, Z.-Y. Chen, Prof. G.-F. Luo, and Prof. Z.-J. Shang

State Key Laboratory of Oral & Maxillofacial Reconstruction and Regeneration, Key Laboratory of Oral Biomedicine Ministry of Education, Hubei Key Laboratory of Stomatology, School & Hospital of Stomatology, Wuhan University, Wuhan 430079, P. R. China

E-mail: luo.guofeng@whu.edu.cn (Prof. G.-F. Luo); shangzhengjun@whu.edu.cn (Prof. Z.-J. Shang)

Prof. Z.-J. Shang

Taikang Center for Life and Medical Sciences of Wuhan University, Wuhan 430079, P. R. China

E-mail: shangzhengjun@whu.edu.cn (Prof. Z.-J. Shang)

*Corresponding authors.

**
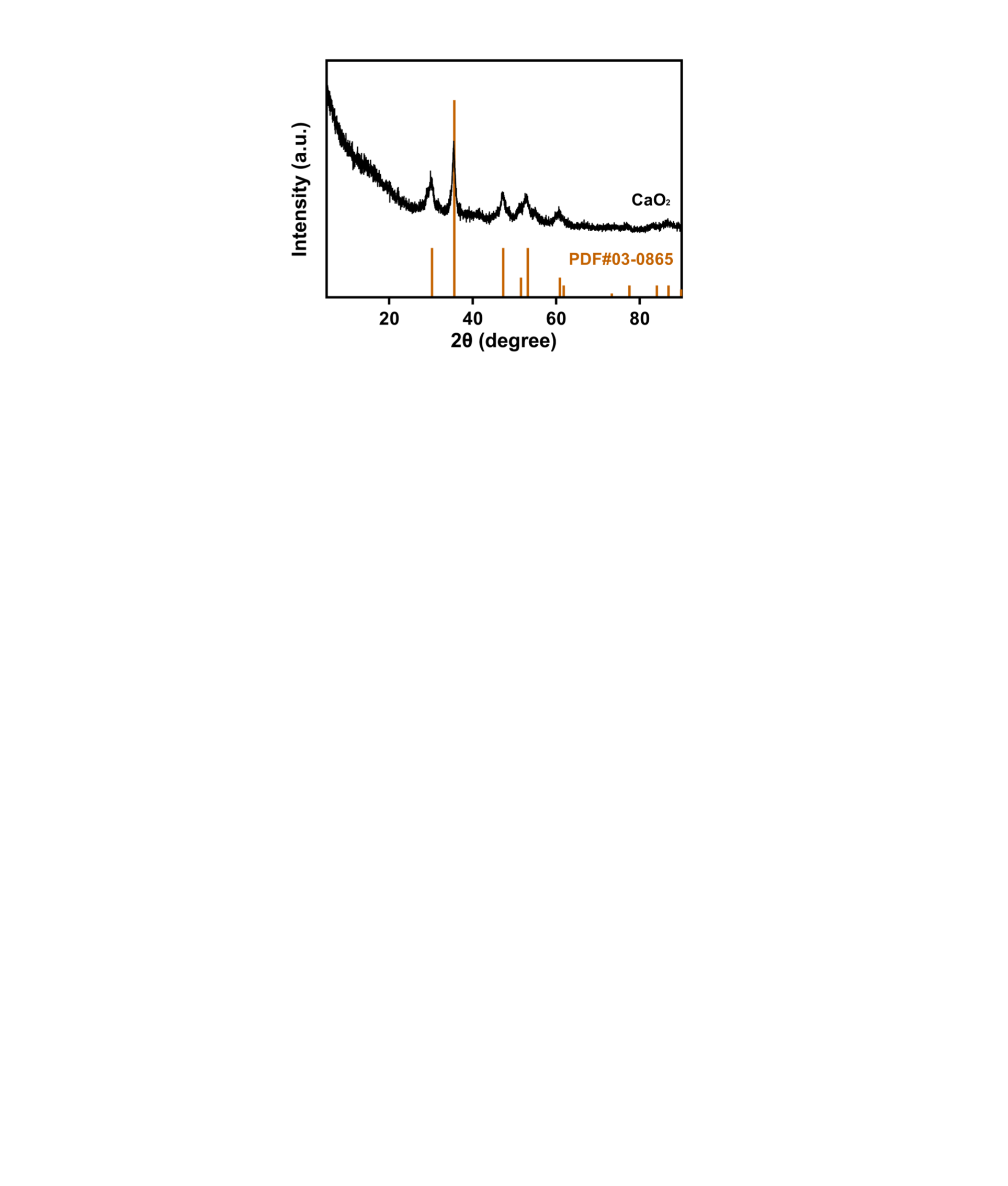
**

**Figure S1.** XRD patterns of CaO_2_ and tetragonal CaO_2_ standard card (PDF#03-0865).


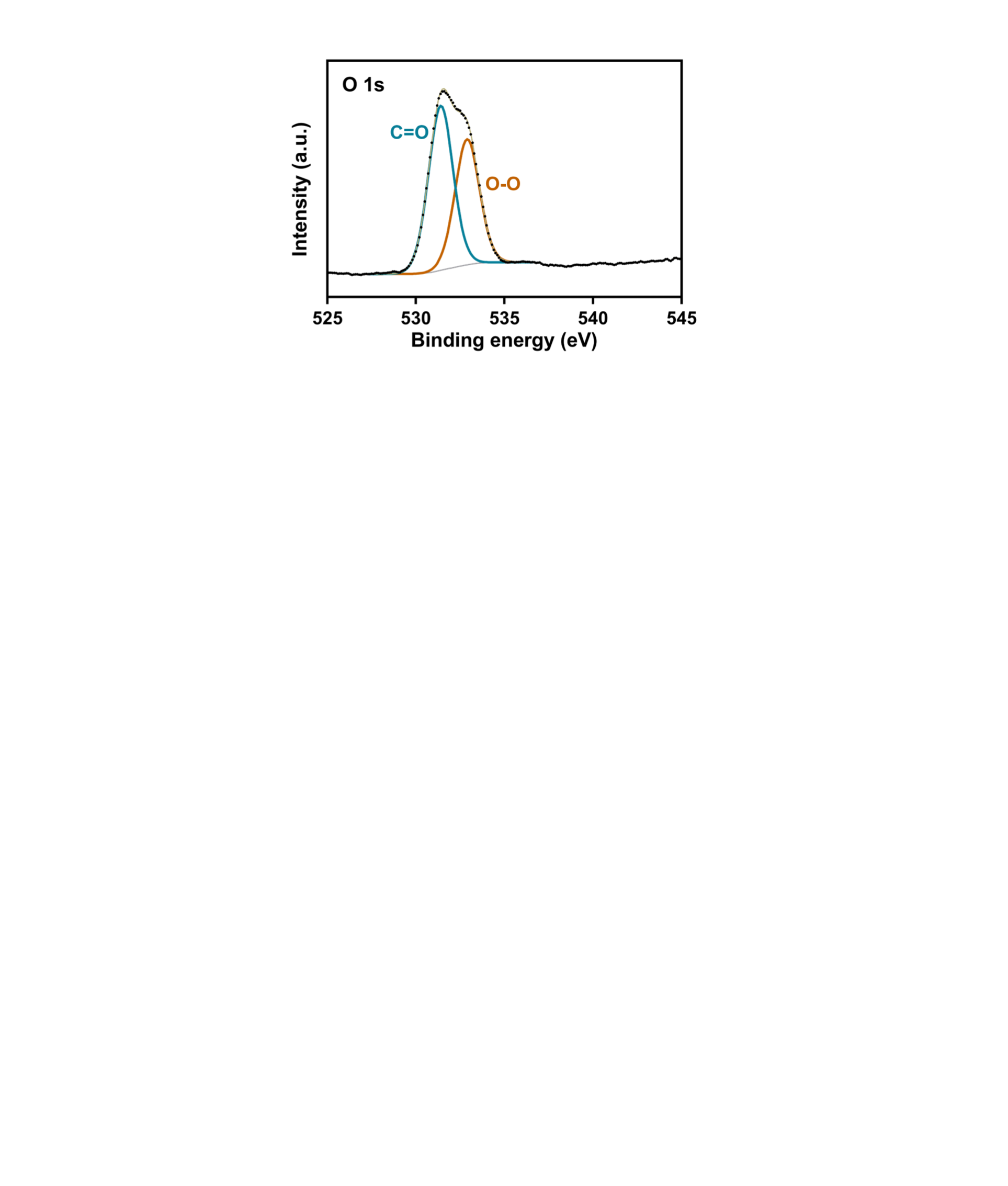


**Figure S2.** High-resolution XPS spectrum of O in CaO_2_@CuMOF@HAP.

**
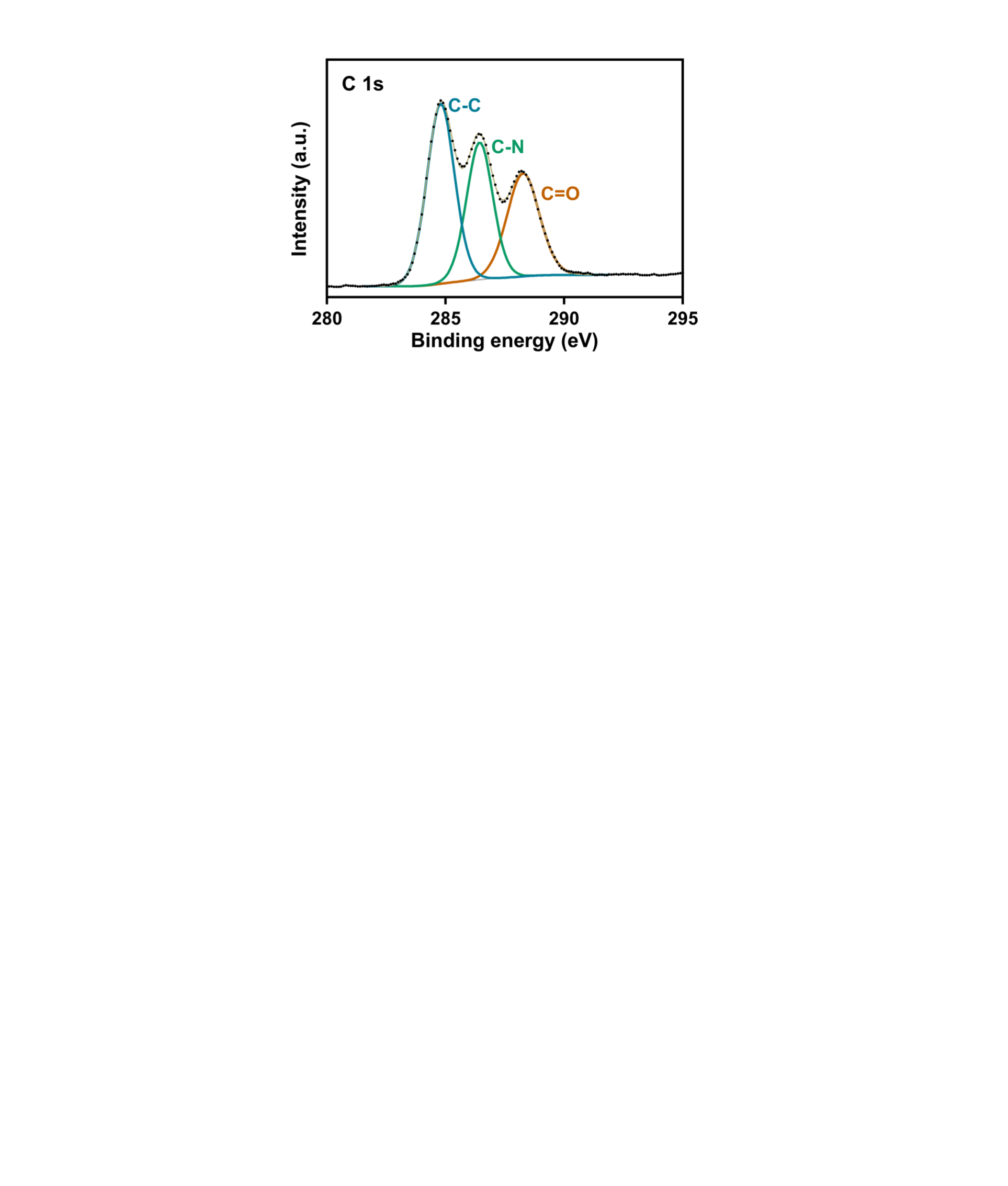
**

**Figure S3.** High-resolution XPS spectrum of C in CaO_2_@CuMOF@HAP.

**
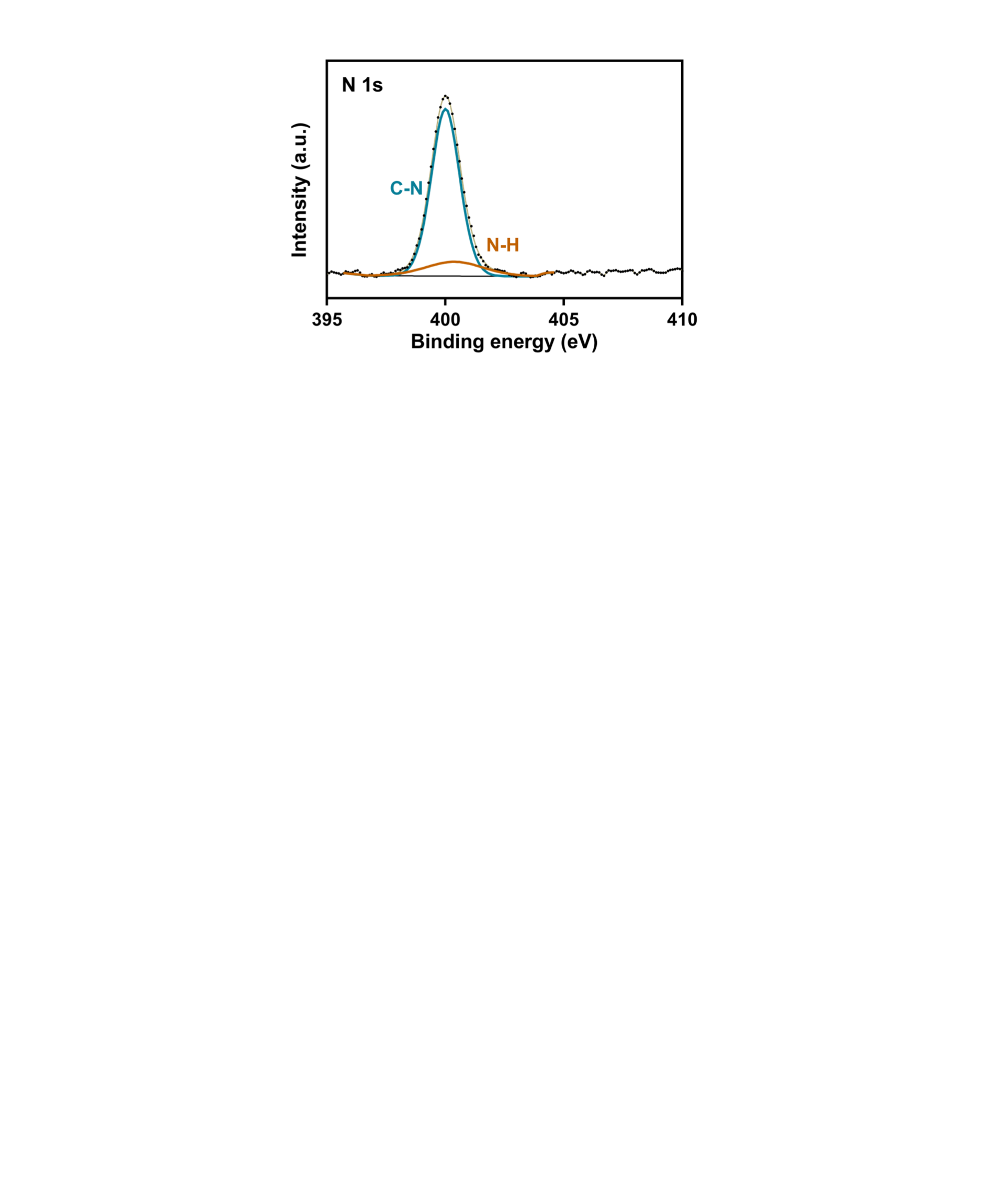
**

**Figure S4.** High-resolution XPS spectrum of N in CaO_2_@CuMOF@HAP.


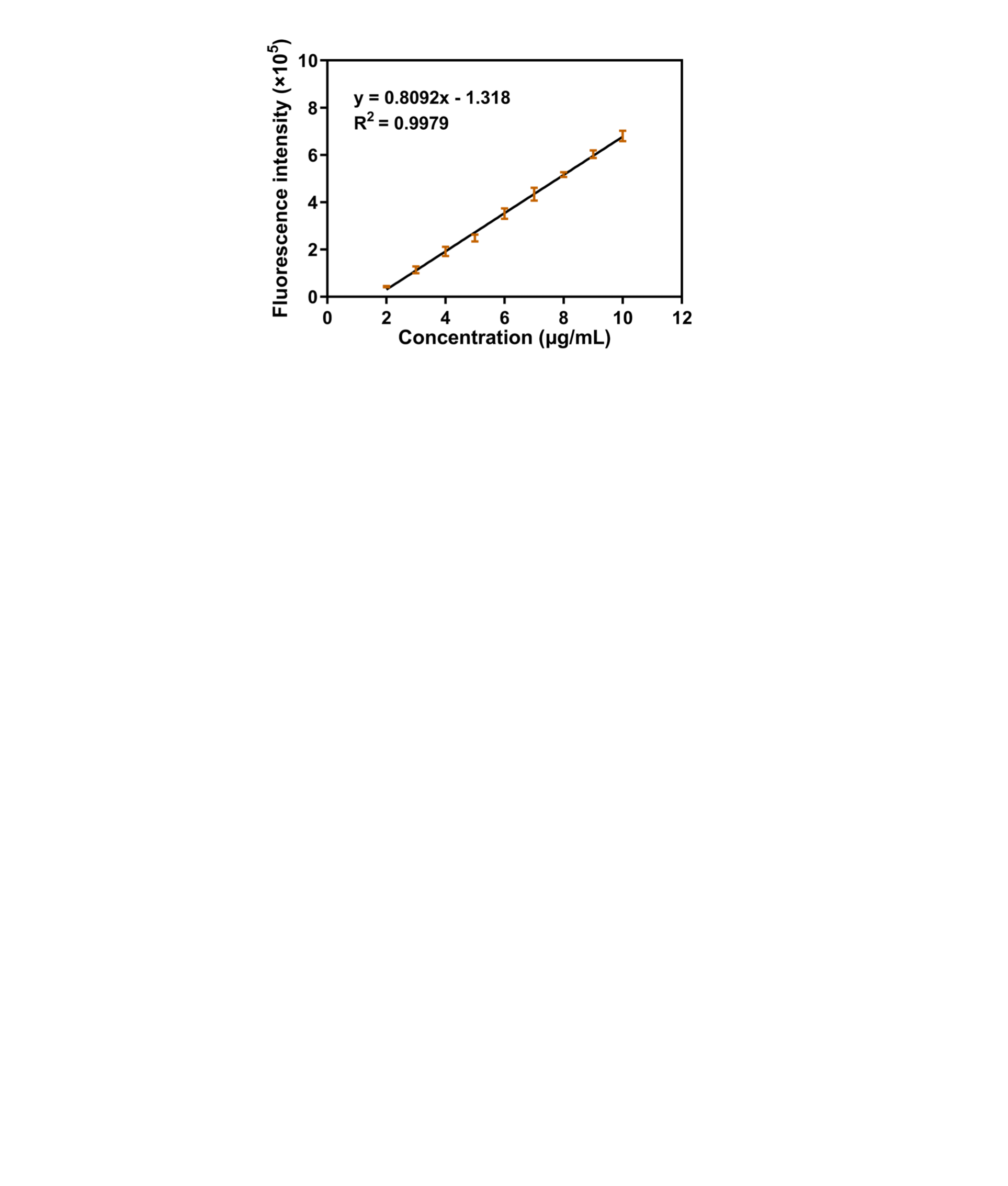


**Figure S5.** Calibration curve of standard FITC-OGP in PBS at 520 nm. Data were performed as the mean ± SD (n = 3 biologically independent samples).


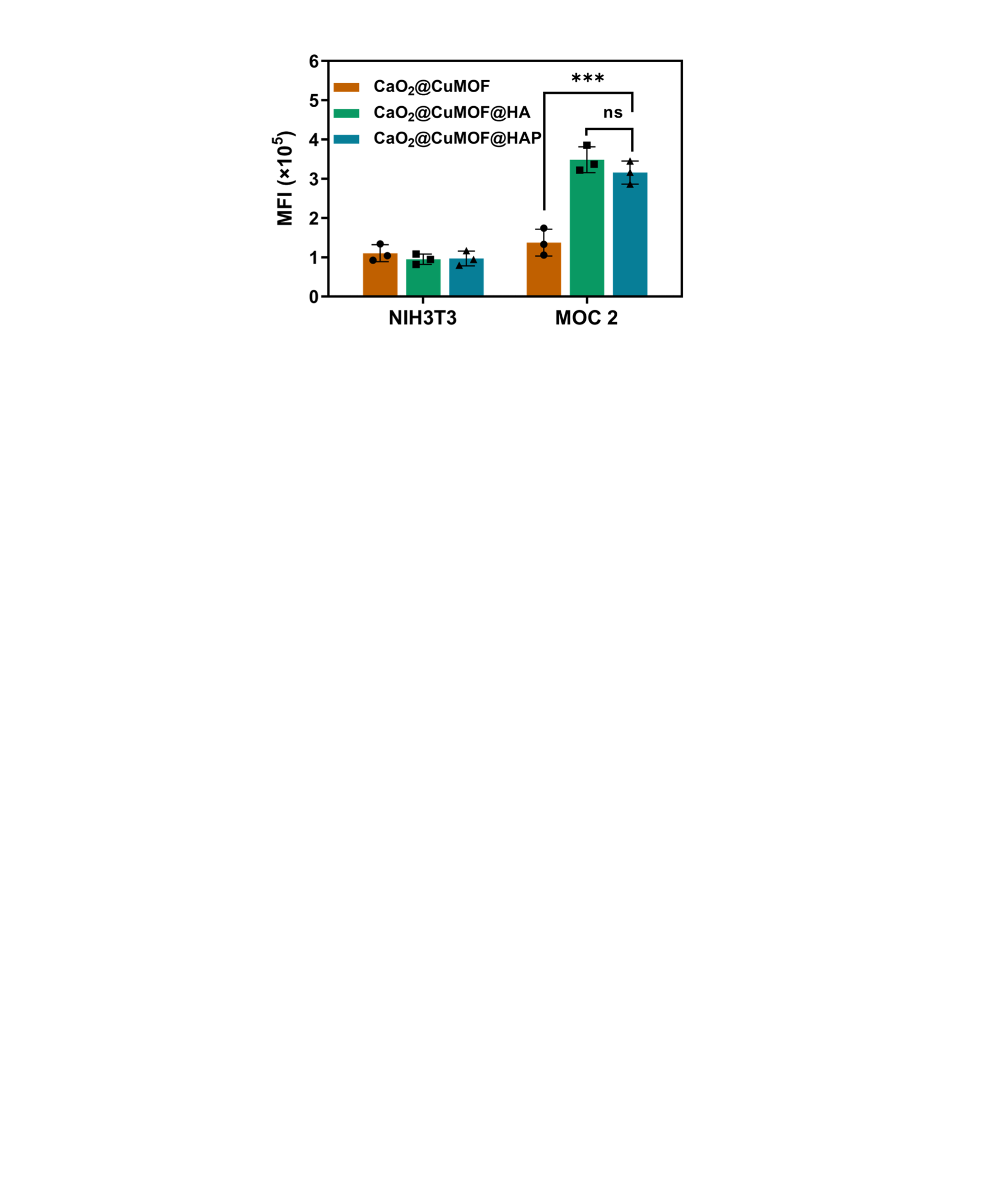


**Figure S6.** The mean fluorescence intensity (MFI) of flow cytometry analysis after NIH3T3 cells and MOC2 cells were treated with rhodamine B-labeled CaO_2_@CuMOF, CaO_2_@CuMOF@HA, and CaO_2_@CuMOF@HAP for 4 h. Data were performed as the mean ± SD (n = 3 biologically independent samples). Two-way ANOVA with Tukey’s post-hoc test was used for multiple comparisons. ****p* < 0.001.


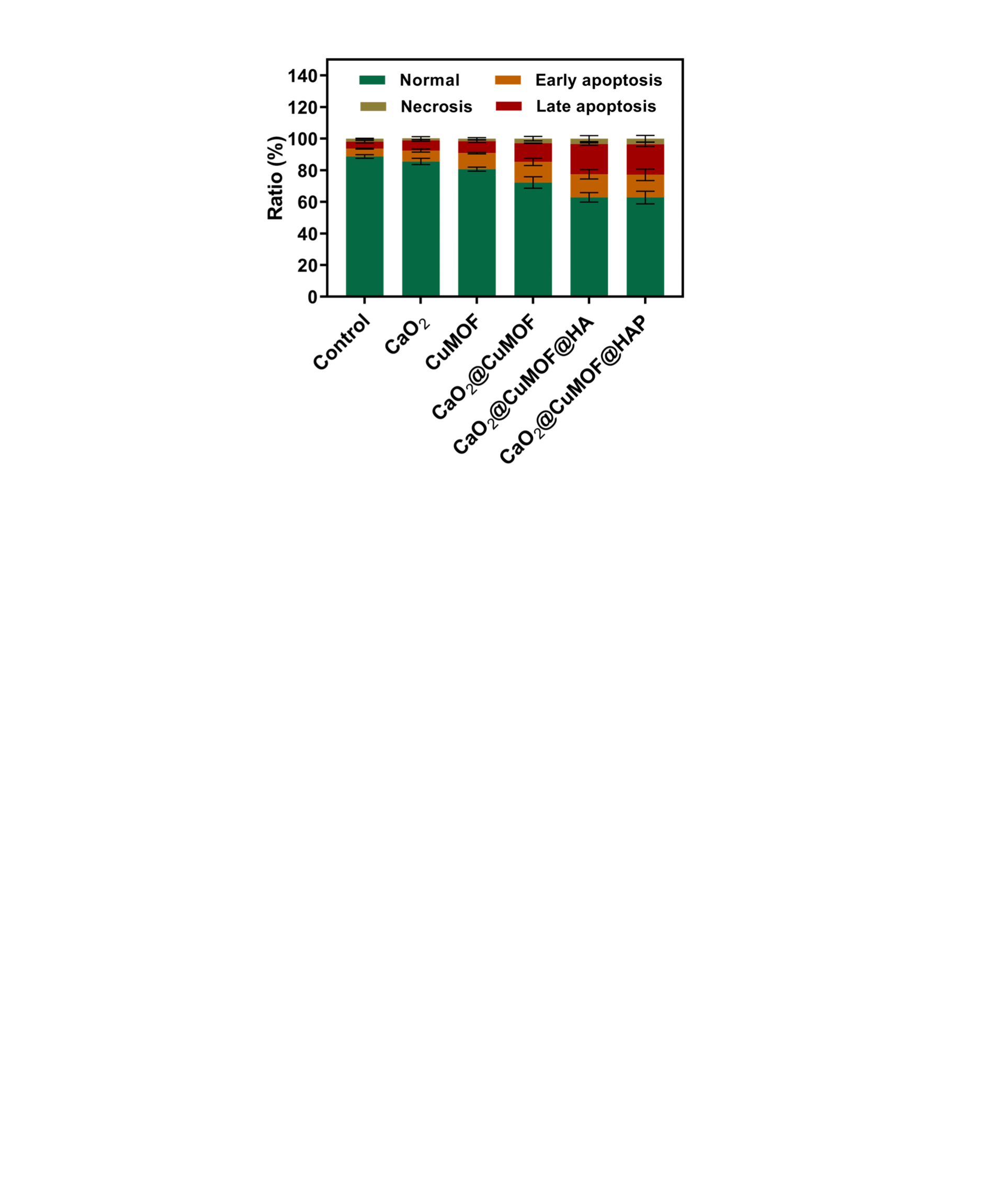


**Figure S7.** Apoptosis/necrosis analysis of MOC2 cells treated with PBS, CaO_2_, CuMOF, CaO_2_@CuMOF, CaO_2_@CuMOF@HA, and CaO_2_@CuMOF@HAP nanoparticles for 24 h. Data were performed as the mean ± SD (n = 3 biologically independent samples).


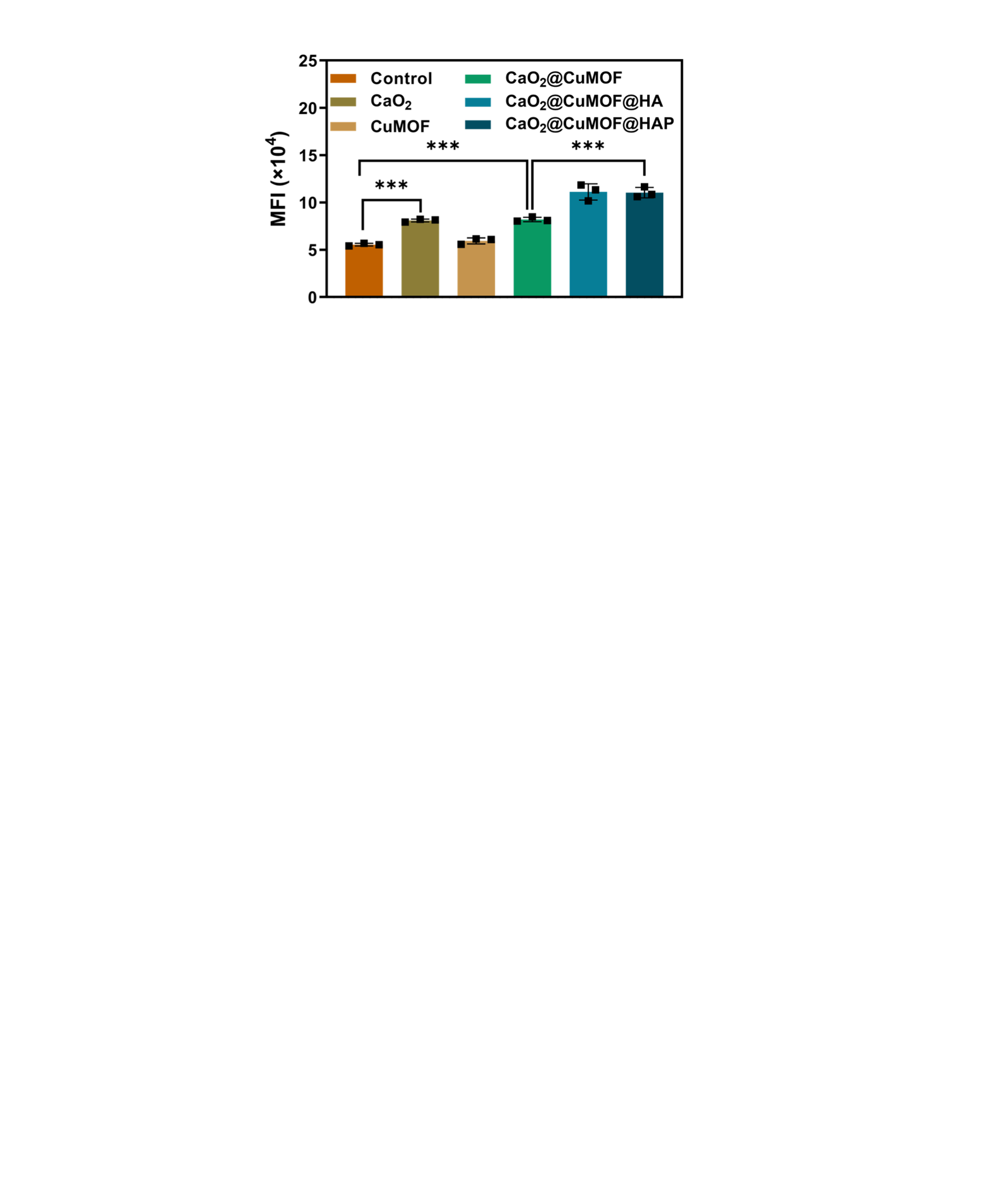


**Figure S8.** MFI of Fluo-4 AM in MOC2 cells after treated with PBS, CaO_2_, CuMOF, CaO_2_@CuMOF, CaO_2_@CuMOF@HA, and CaO_2_@CuMOF@HAP for 6 h detecting with flow cytometry. Data were performed as the mean ± SD (n = 3 biologically independent samples). One-way ANOVA with Tukey’s post-hoc test was used for multiple comparisons. ****p* < 0.001.


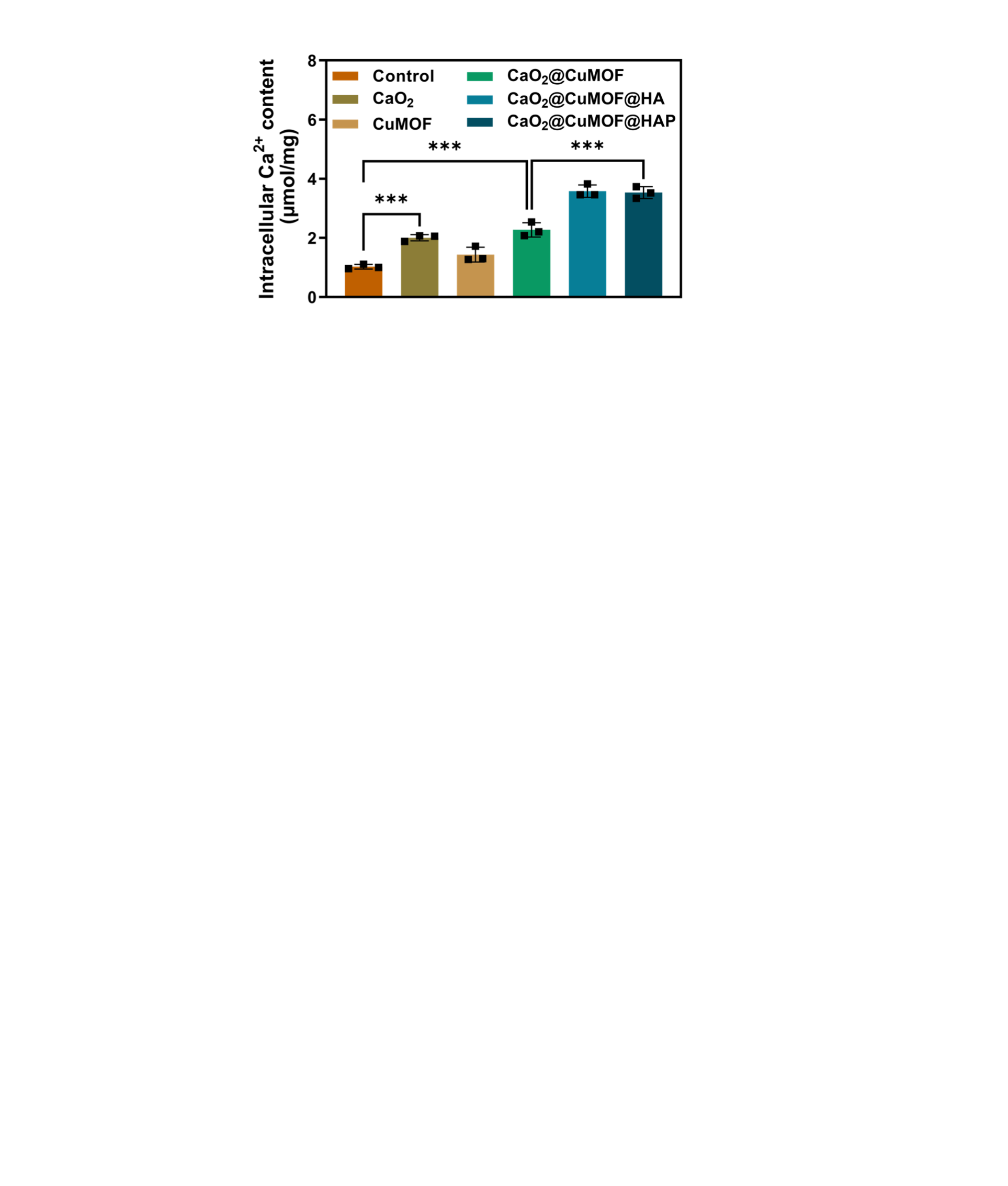


**Figure S9.** Intracellular Ca^2+^ levels of MOC2 cells after treated with PBS, CaO_2_, CuMOF, CaO_2_@CuMOF, CaO_2_@CuMOF@HA, and CaO_2_@CuMOF@HAP for 6 h detecting with a Calcium (Ca) Colorimetric Assay Kit. Data were performed as the mean ± SD (n = 3 biologically independent samples). One-way ANOVA with Tukey’s post-hoc test was used for multiple comparisons. ****p* < 0.001.


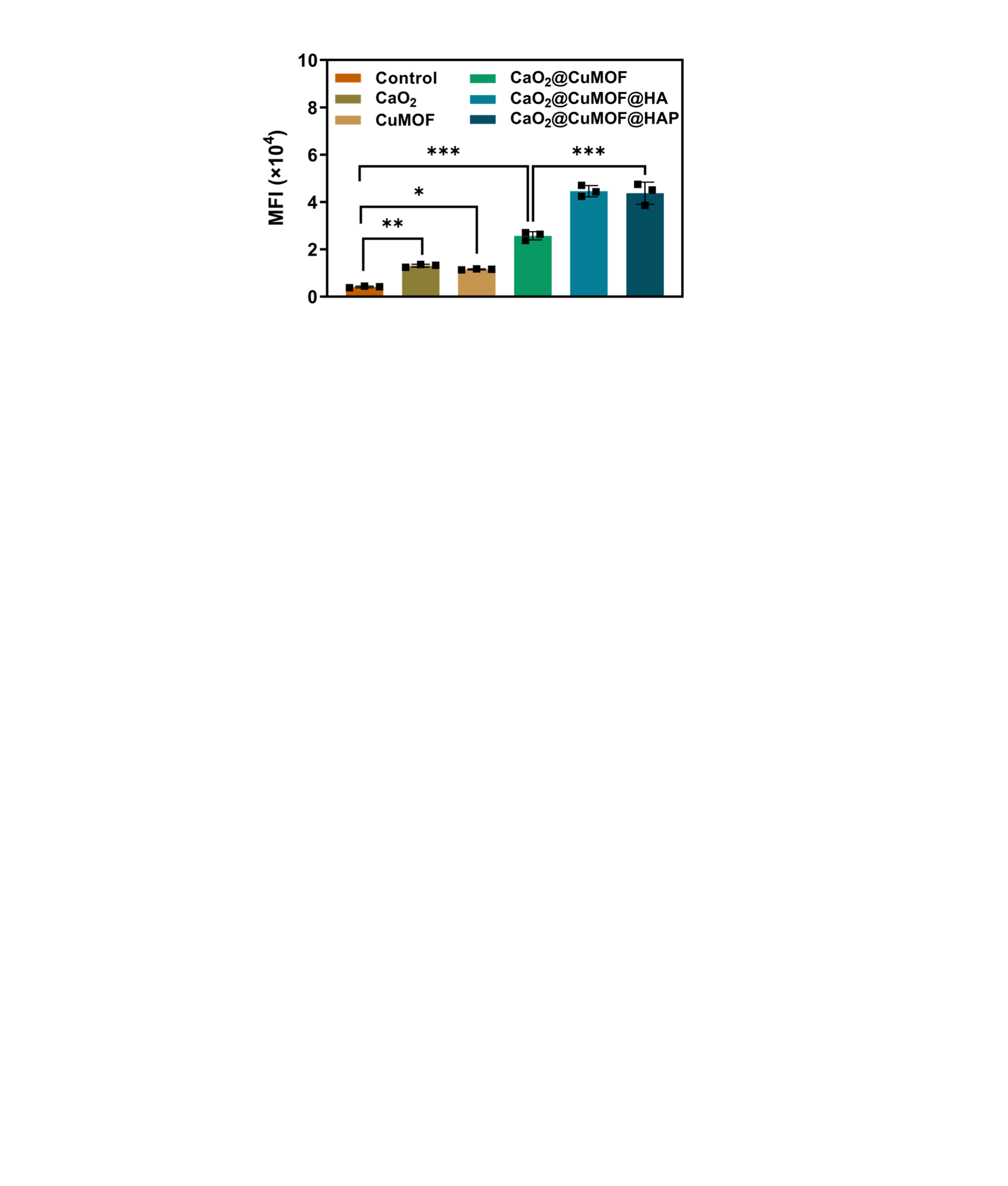


**Figure S10.** MFI of DCF in MOC2 cells after treated with PBS, CaO_2_, CuMOF, CaO_2_@CuMOF, CaO_2_@CuMOF@HA, and CaO_2_@CuMOF@HAP for 6 h detecting with flow cytometry. Data were performed as the mean ± SD (n = 3 biologically independent samples). One-way ANOVA with Tukey’s post-hoc test was used for multiple comparisons. **p* < 0.05, ***p* < 0.01, ****p* < 0.001.


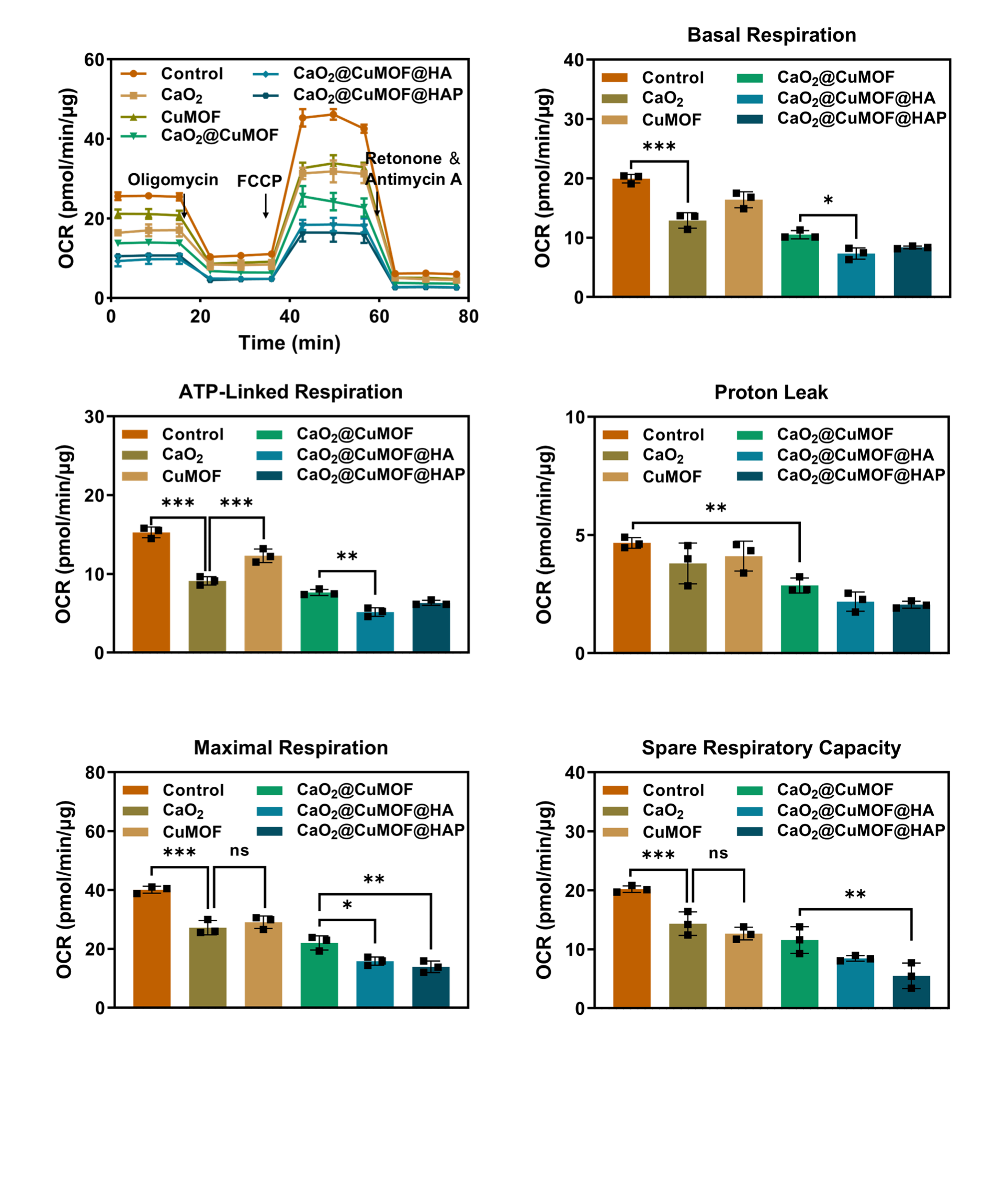


**Figure S11.** OCR from Seahorse XF Mito stress test of MOC2 cells after treated with PBS, CaO_2_, CuMOF, CaO_2_@CuMOF, CaO_2_@CuMOF@HA, and CaO_2_@CuMOF@HAP for 6  h. Data were performed as the mean ± SD (n = 3 biologically independent samples). One-way ANOVA with Tukey’s post-hoc test was used for multiple comparisons. **p* < 0.05, ***p* < 0.01, ****p* < 0.001.


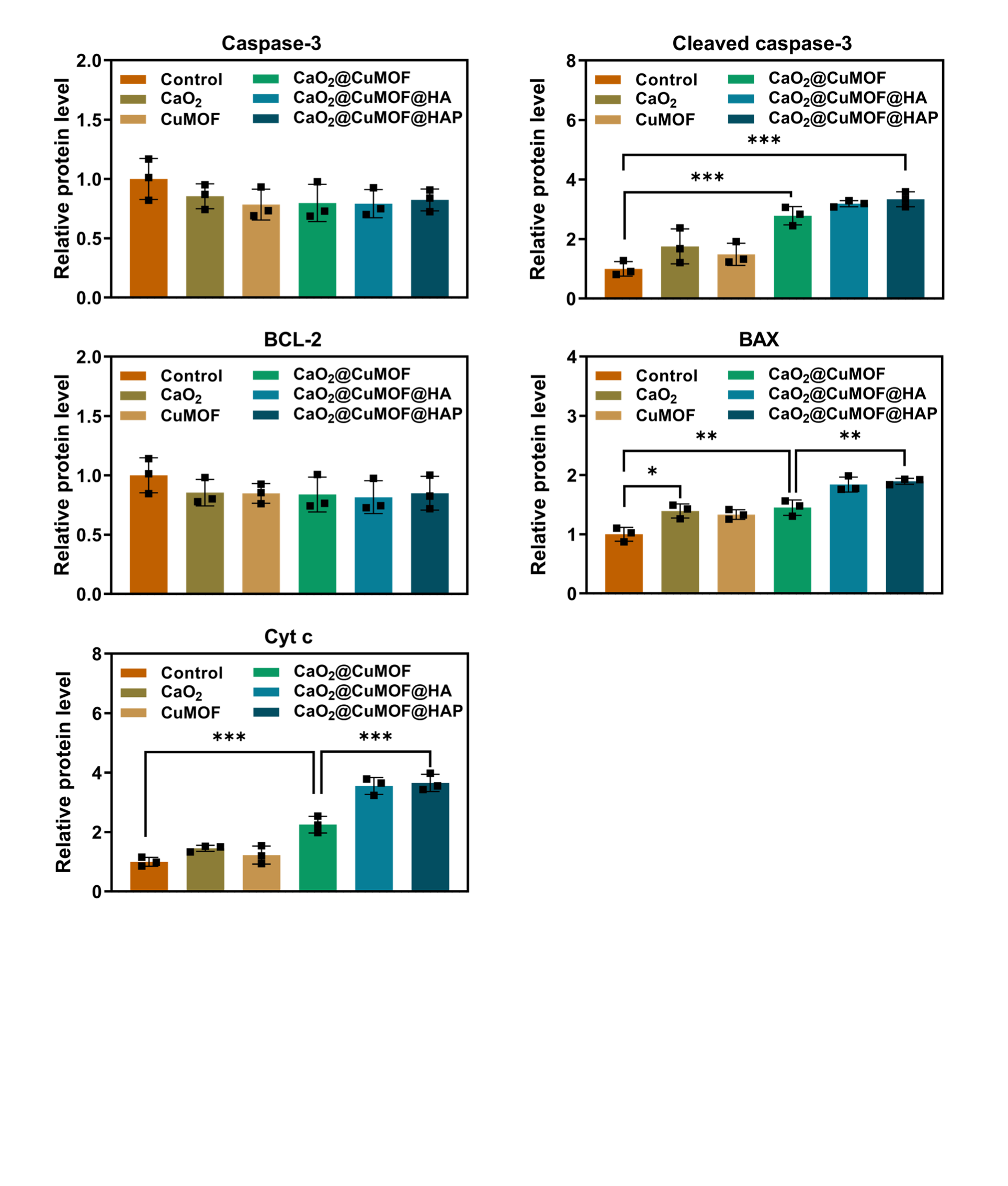


**Figure S12.** The quantitative analysis of caspase-3, cleaved caspase-3, BCL-2, BAX, and cytochrome c (Cyt c) via western blotting. Data were performed as the mean ± SD (n = 3 biologically independent samples). One-way ANOVA with Tukey’s post-hoc test was used for multiple comparisons. **p* < 0.05, ***p* < 0.01, ****p* < 0.001.


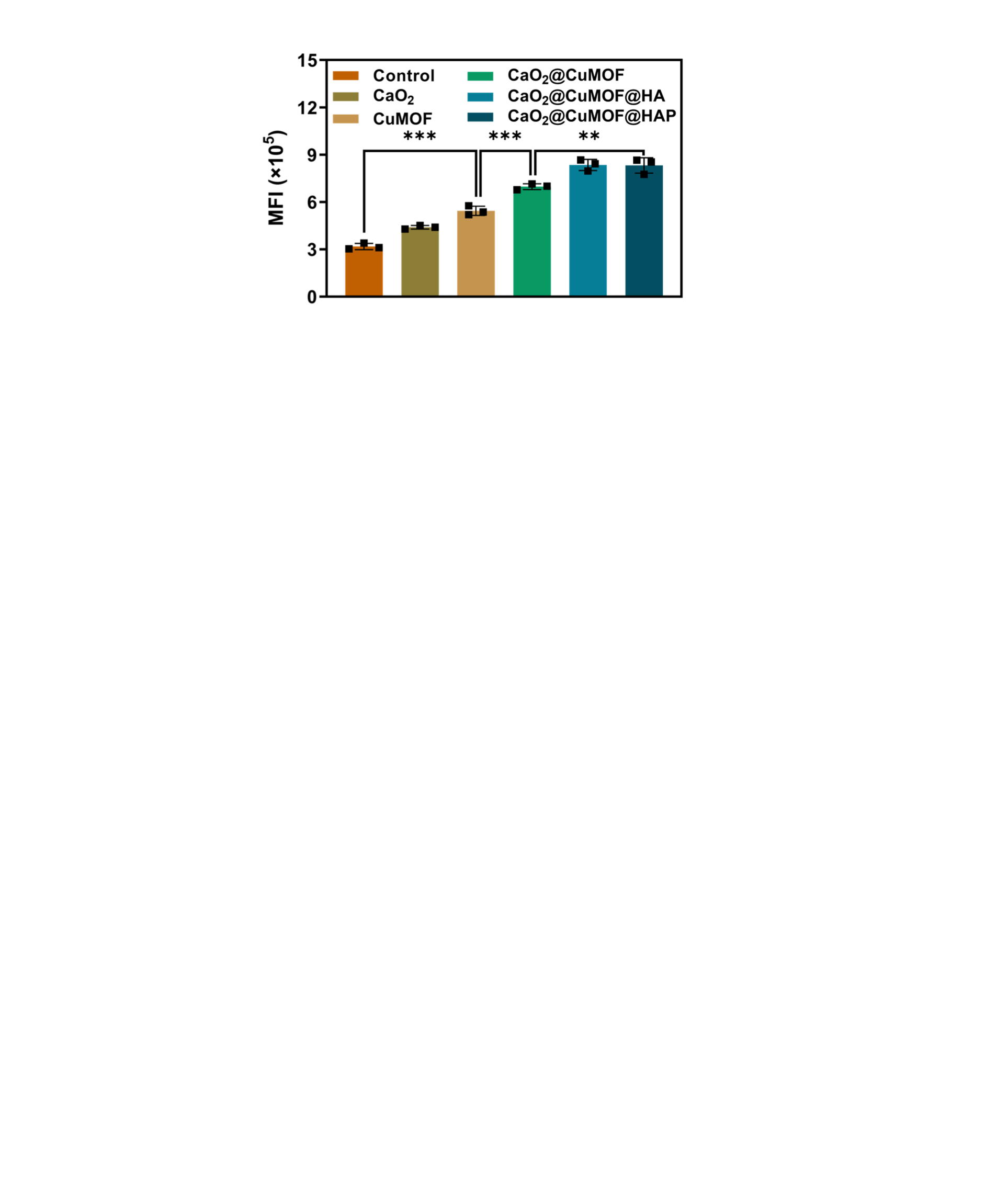


**Figure S13.** MFI of CS-1 in MOC2 cells after treated with PBS, CaO_2_, CuMOF, CaO_2_@CuMOF, CaO_2_@CuMOF@HA, and CaO_2_@CuMOF@HAP for 6 h detecting with flow cytometry. Data were performed as the mean ± SD (n = 3 biologically independent samples). One-way ANOVA with Tukey’s post-hoc test was used for multiple comparisons. ***p* < 0.01, ****p* < 0.001.


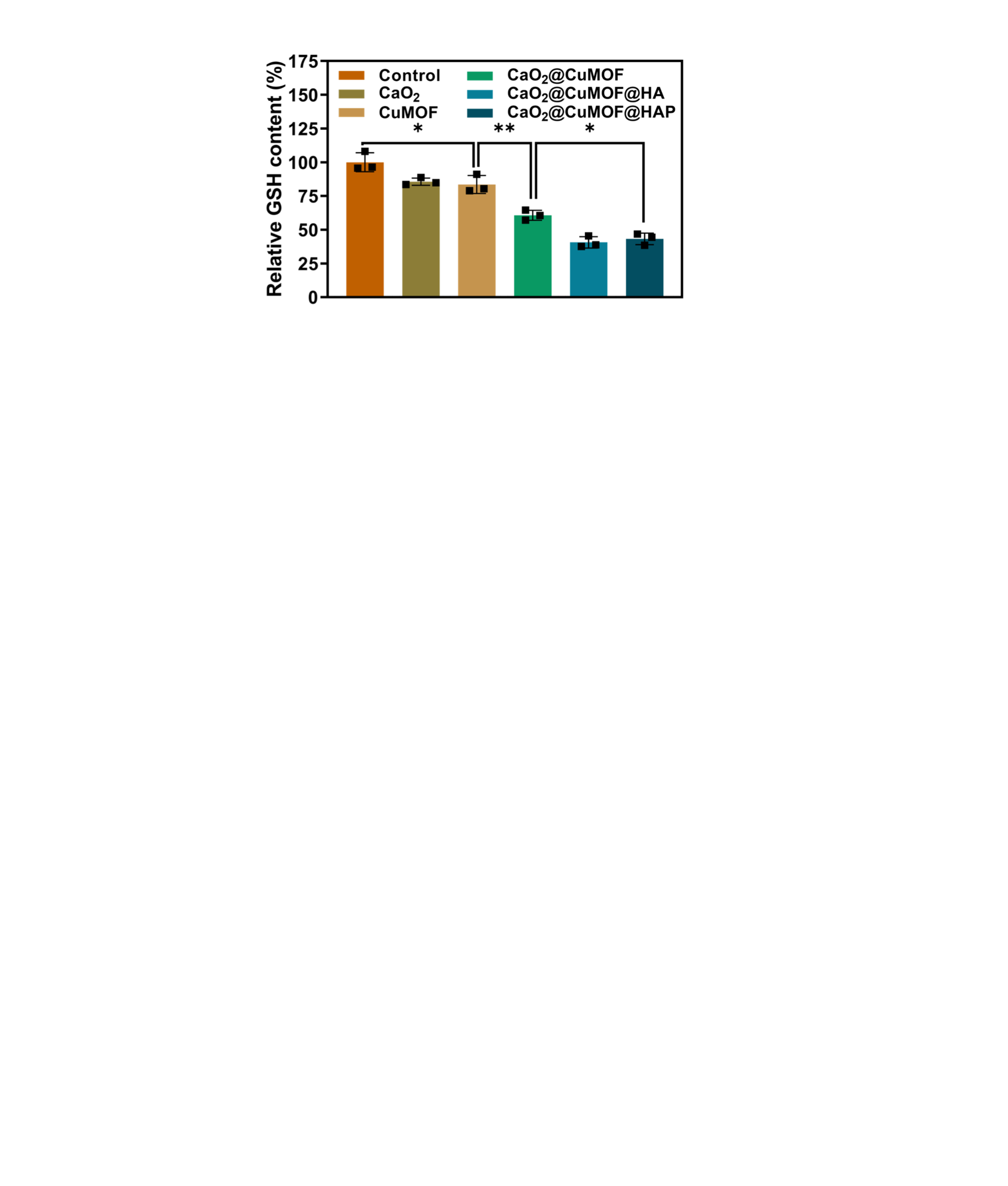


**Figure S14.** The relative GSH content in MOC2 cells after treated with PBS, CaO_2_, CuMOF, CaO_2_@CuMOF, CaO_2_@CuMOF@HA, and CaO_2_@CuMOF@HAP for 6 h detecting with a GSH Assay Kit. Data were performed as the mean ± SD (n = 3 biologically independent samples). One-way ANOVA with Tukey’s post-hoc test was used for multiple comparisons. **p* < 0.05, ***p* < 0.01.


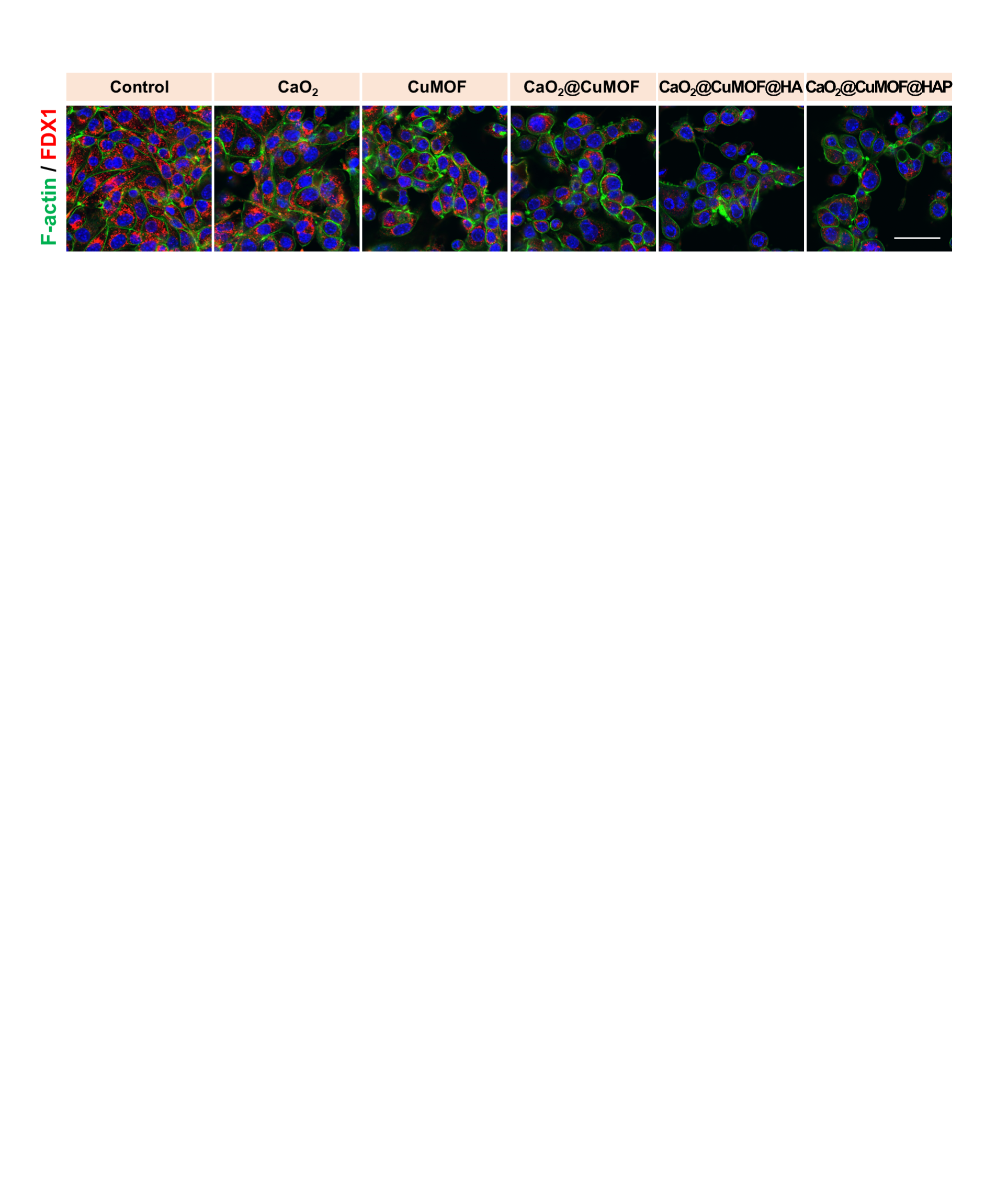


**Figure S15.** Immunofluorescent staining of FDX1 and F-actin in MOC2 cells following different treatments. Green: F-actin (Phalloidin); Red: FDX1; Blue: DAPI. Scale bar: 50 μm.


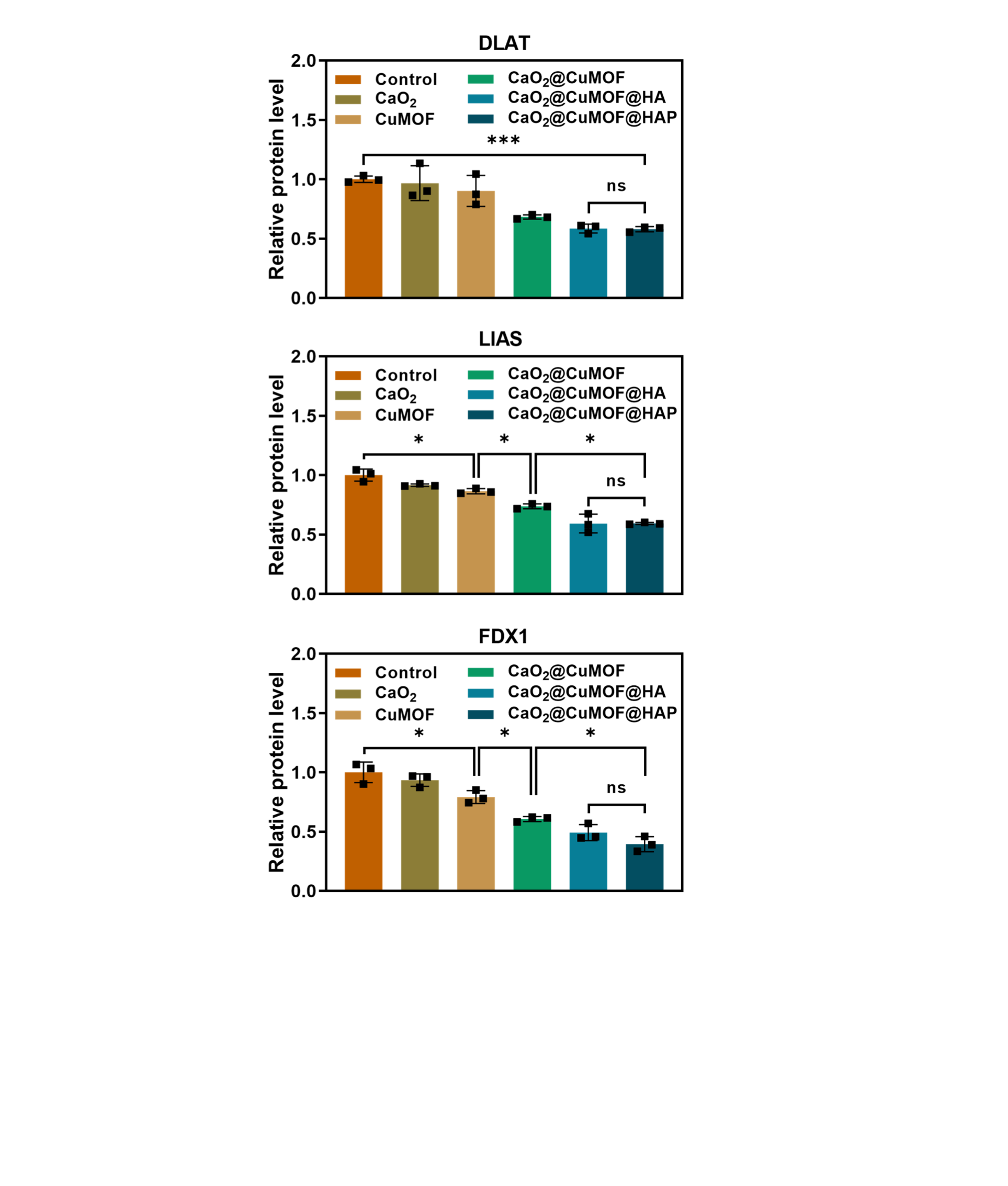


**Figure S16.** The quantitative analysis of DLAT, LIAS, and FDX1 via western blotting. Data were performed as the mean ± SD (n = 3 biologically independent samples). One-way ANOVA with Tukey’s post-hoc test was used for multiple comparisons. **p* < 0.05, ****p* < 0.001.


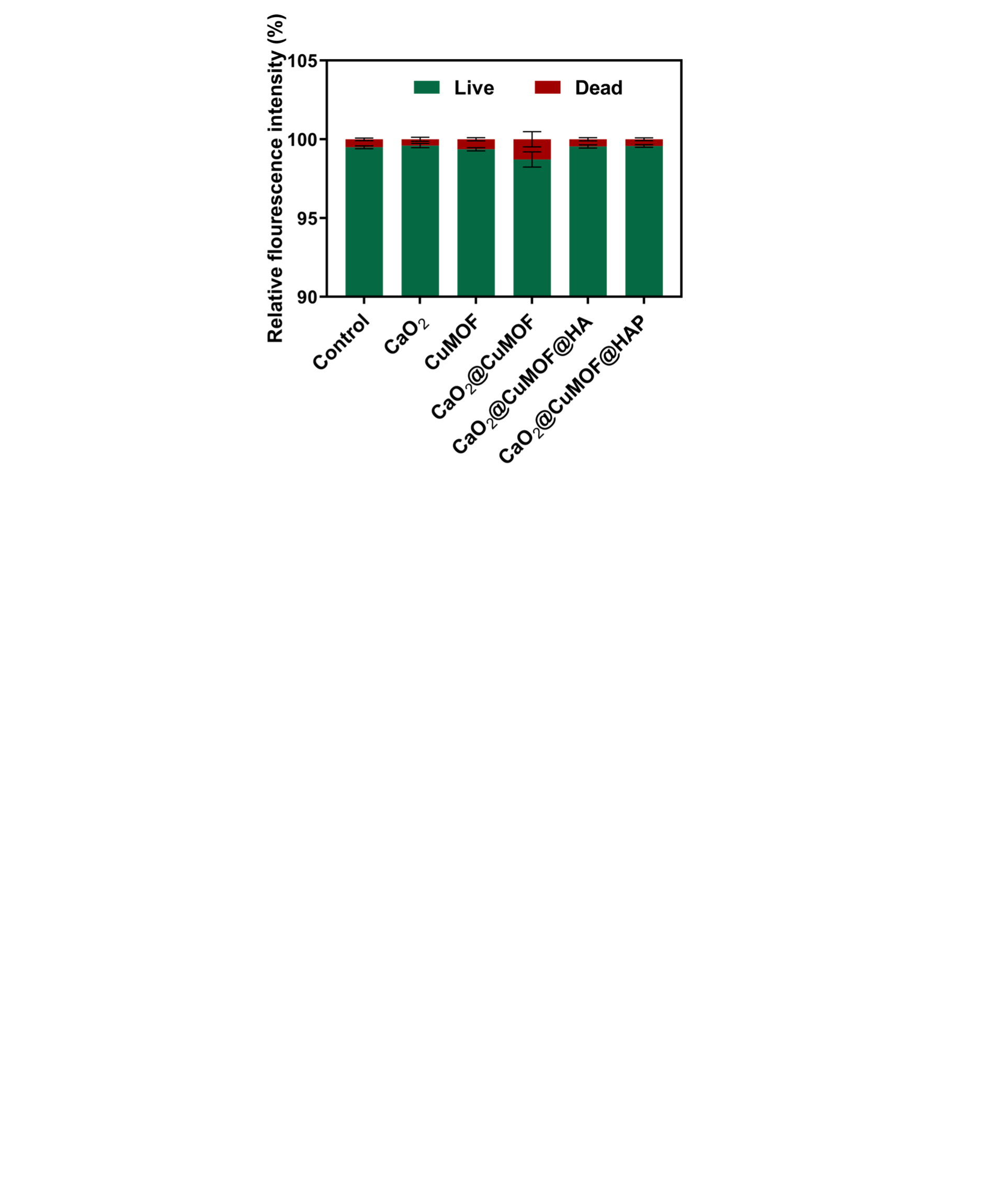


**Figure S17.** The relative fluorescence ratio of MC3T3 cells treated with PBS, CaO_2_, CuMOF, CaO_2_@CuMOF, CaO_2_@CuMOF@HA, and CaO_2_@CuMOF@HAP nanoparticles for 72 h detecting with Calcein and PI staining. Data were performed as the mean ± SD (n = 3 biologically independent samples).

**
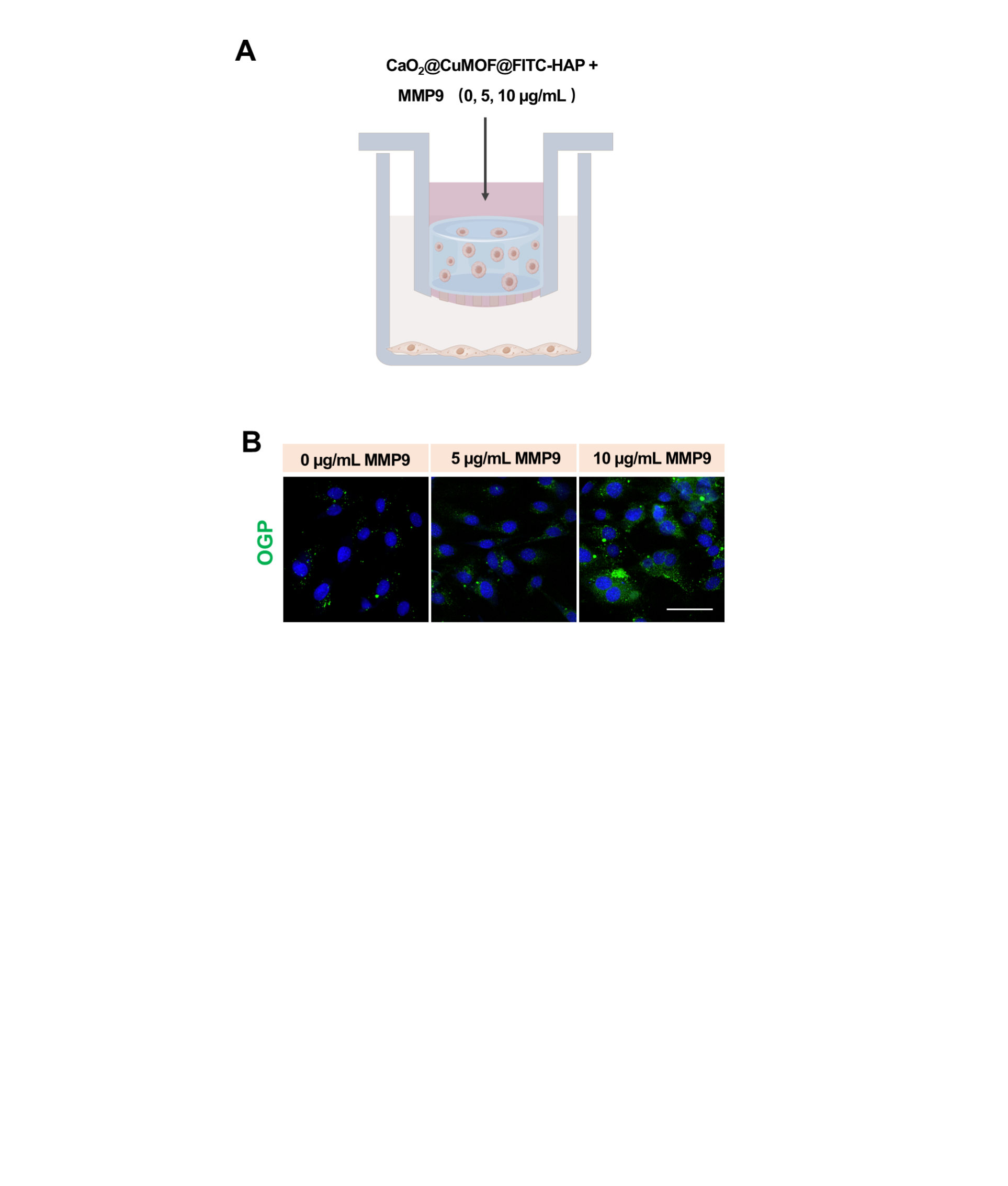
**

**Figure S18.** A) The illustration of Transwell co-culture system. MC3T3 cells were seeded in the lower chamber, while MOC2 cells embedded in matrigel were placed in the upper chamber. CaO₂@CuMOF@FITC- HAP was added to the upper chamber medium under three conditions: (1) 0 μg/mL MMP9, (2) 5 μg/mL MMP9, and (3) 10 μg/mL MMP9. B) CLSM images of MC3T3 cells after different treatments for 24 h. Green: FITC-labeled OGP; Blue: DAPI-stained nuclei. Scale bar: 50 μm.

**
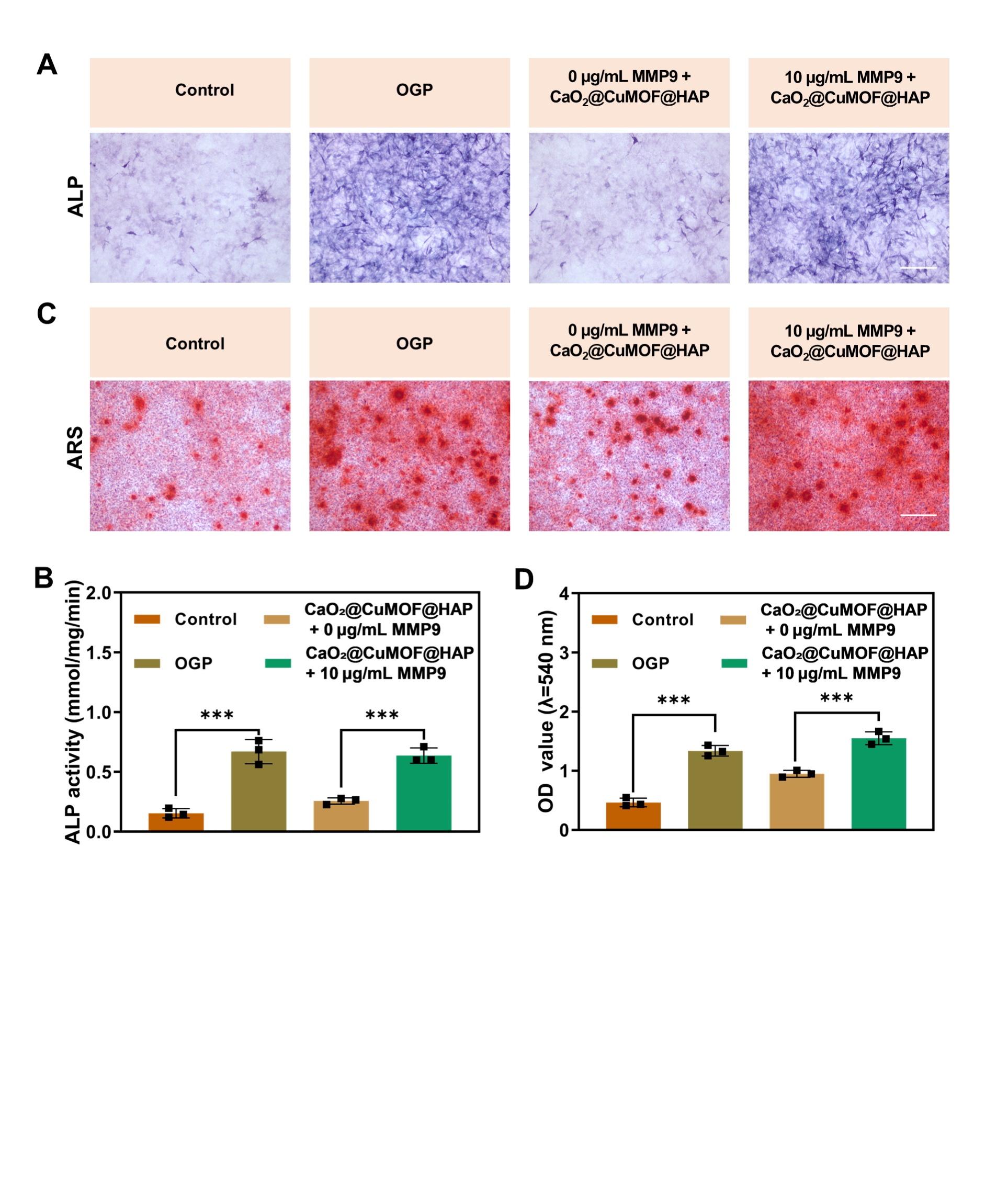
**

**Figure S19.** A) ALP staining and B) quantified ALP activity of MC3T3 cells after various treatments for 7 days. Scale bar: 500 μm. C) ARS staining of MC3T3 cells after various treatments for 21 days. Scale bar: 500 μm. D) Quantitative analysis of calcium nodules after various treatments for 21 days. Data were performed as the mean ± SD (n = 3 biologically independent samples). One-way ANOVA with Tukey’s post-hoc test was used for multiple comparisons in B) and D). ****p* < 0.001.

**
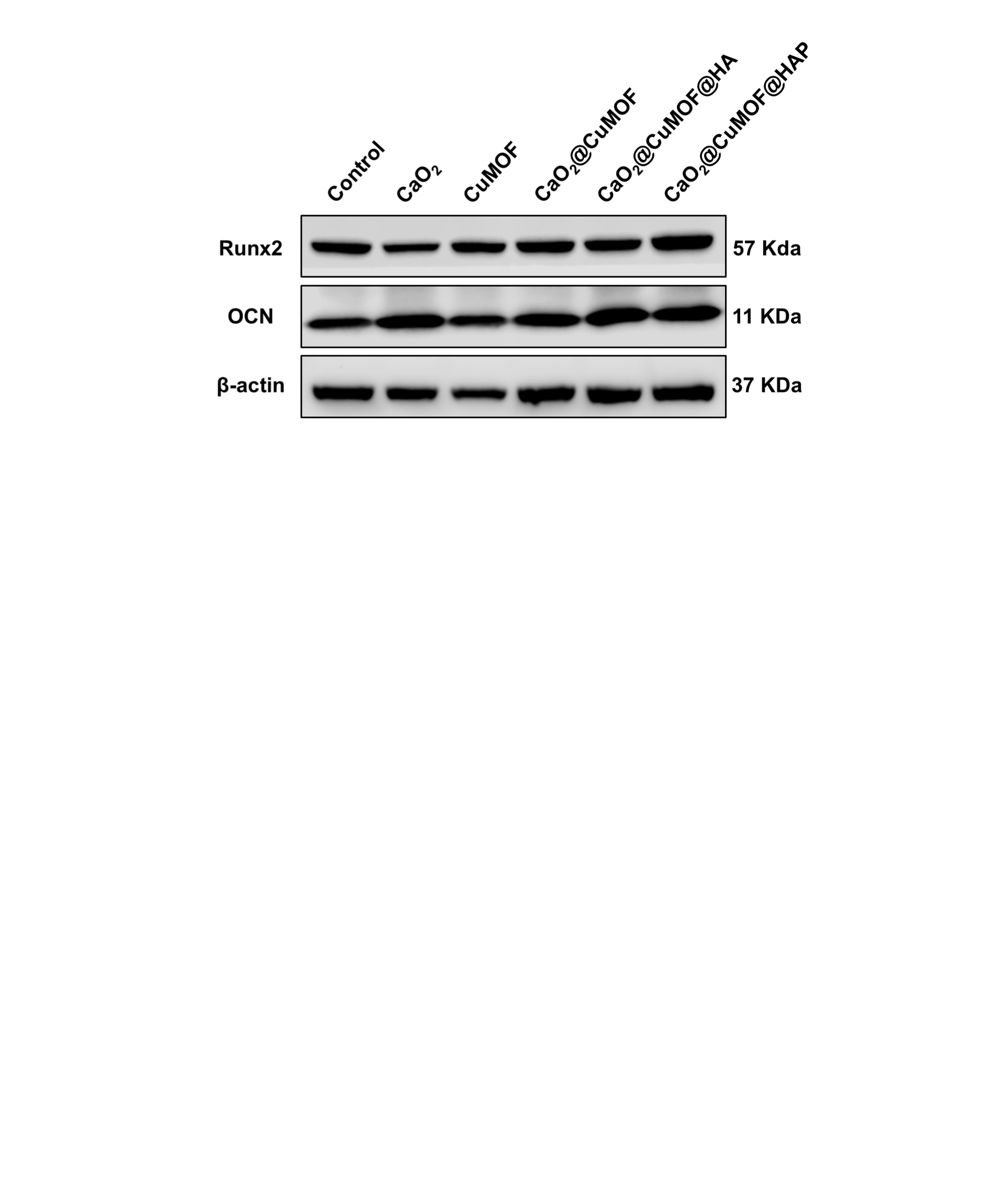
**

**Figure S20.** Western blot analysis of Runx2 and OCN.


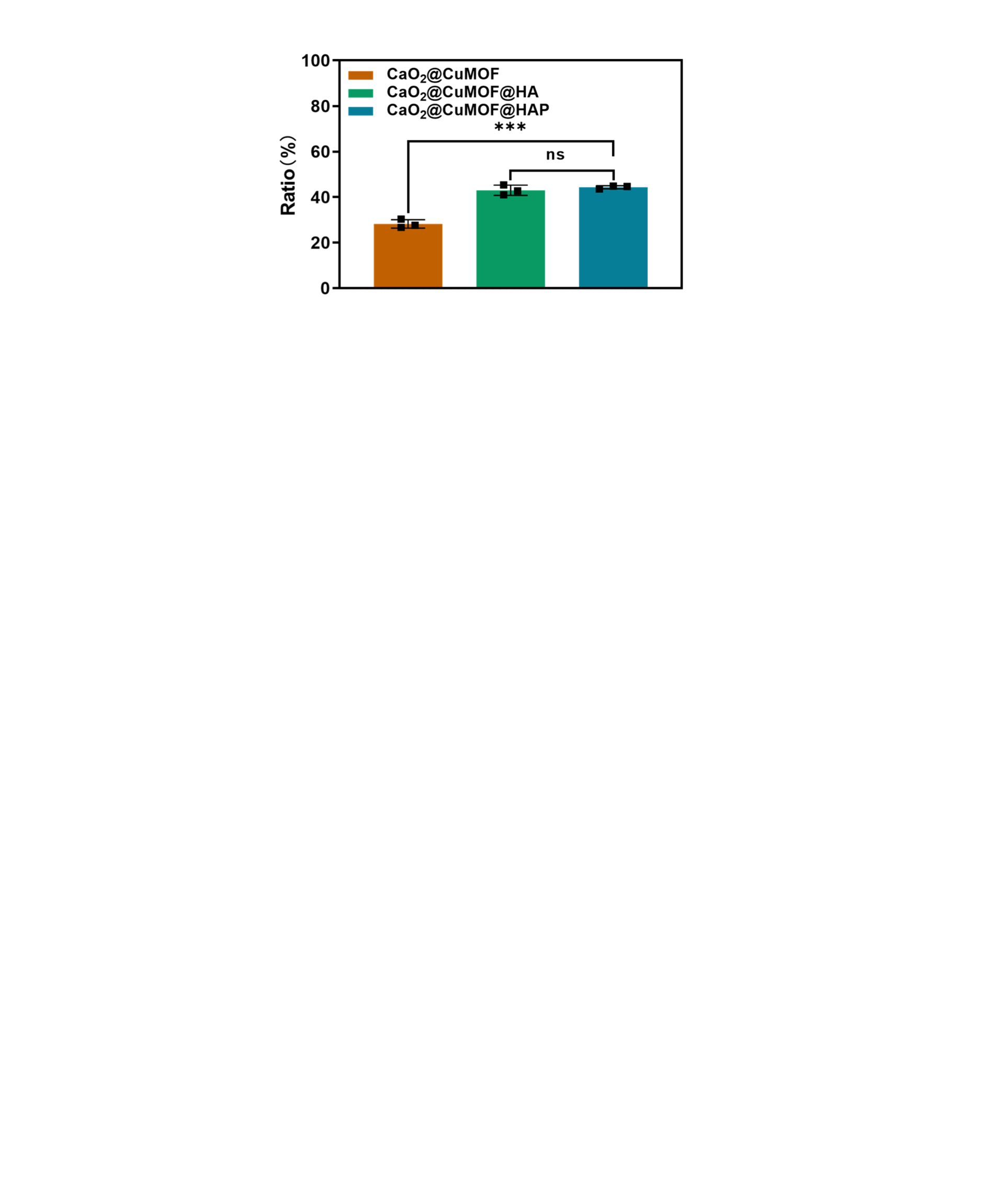


**Figure S21.** The relative fluorescence ratio of isolated tumors at 24 h postinjection. Data were performed as the mean ± SD (n = 3 biologically independent samples). One-way ANOVA with Tukey’s post-hoc test was used for multiple comparisons. ****p* < 0.001.


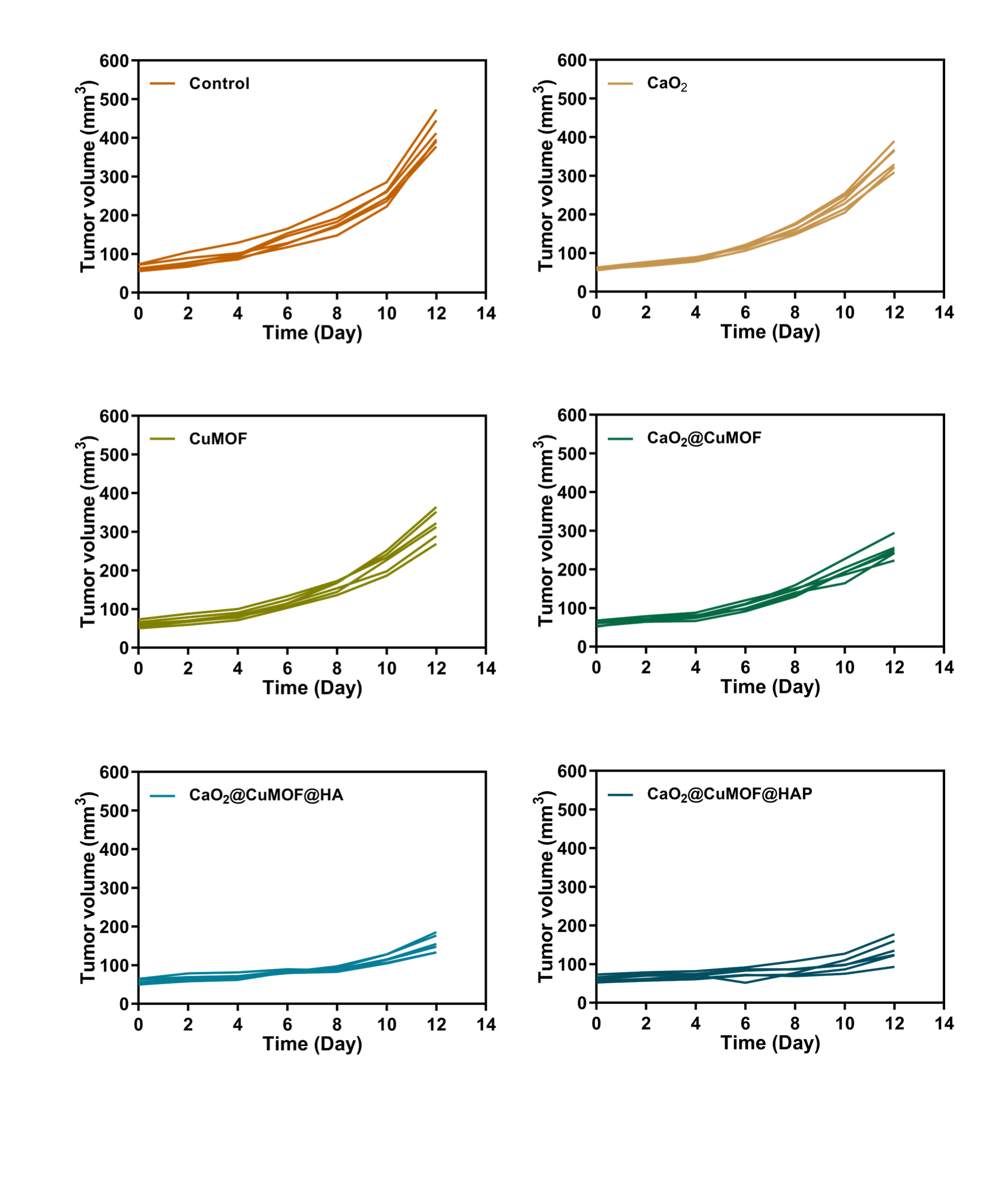


**Figure S22.** The individual tumor growth trajectories following different treatments (n = 6 biologically independent samples).


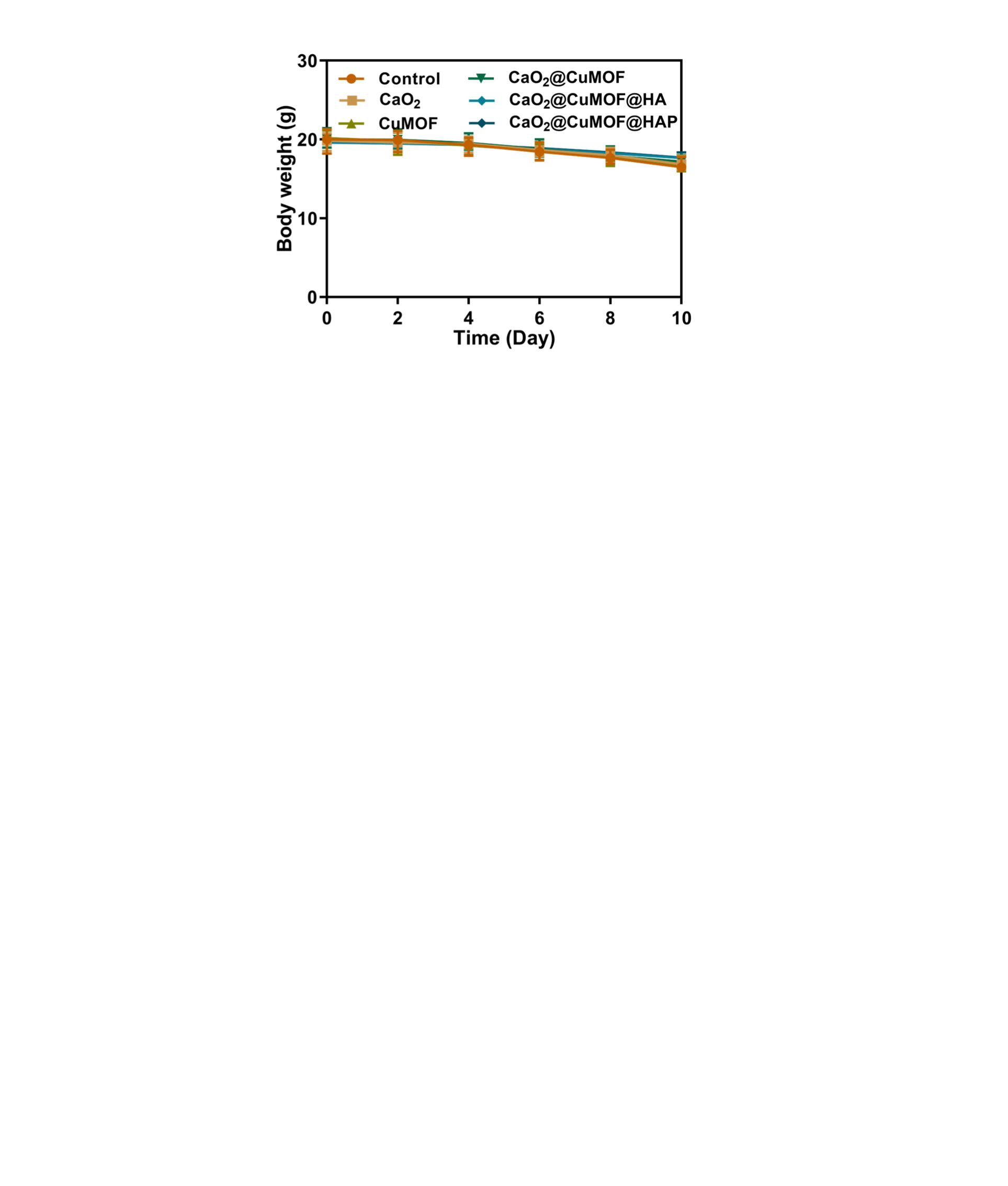


**Figure S23.** Body weight changes of the tumor-bearing mice during different treatments. Data were performed as the mean ± SD (n = 6 biologically independent samples).


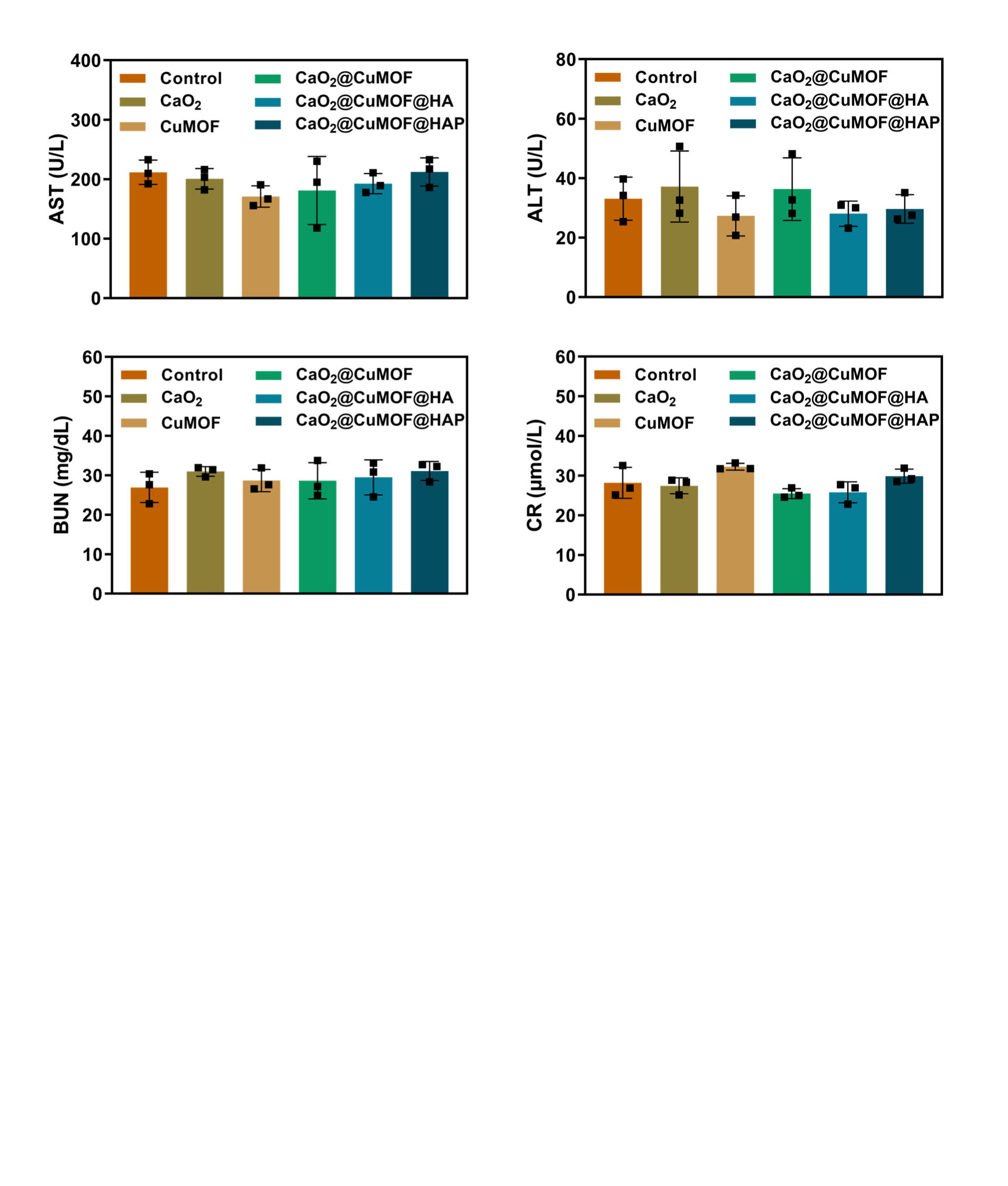


**Figure S24.** Serum biochemical analysis of hepatic function markers, aspartate aminotransferase (AST) and alanine aminotransferase (ALT), as well as renal function markers, blood urea nitrogen (BUN) and creatinine (CR). Data were performed as the mean ± SD (n = 3 biologically independent samples). One-way ANOVA with Tukey’s post-hoc test was used for multiple comparisons.

**
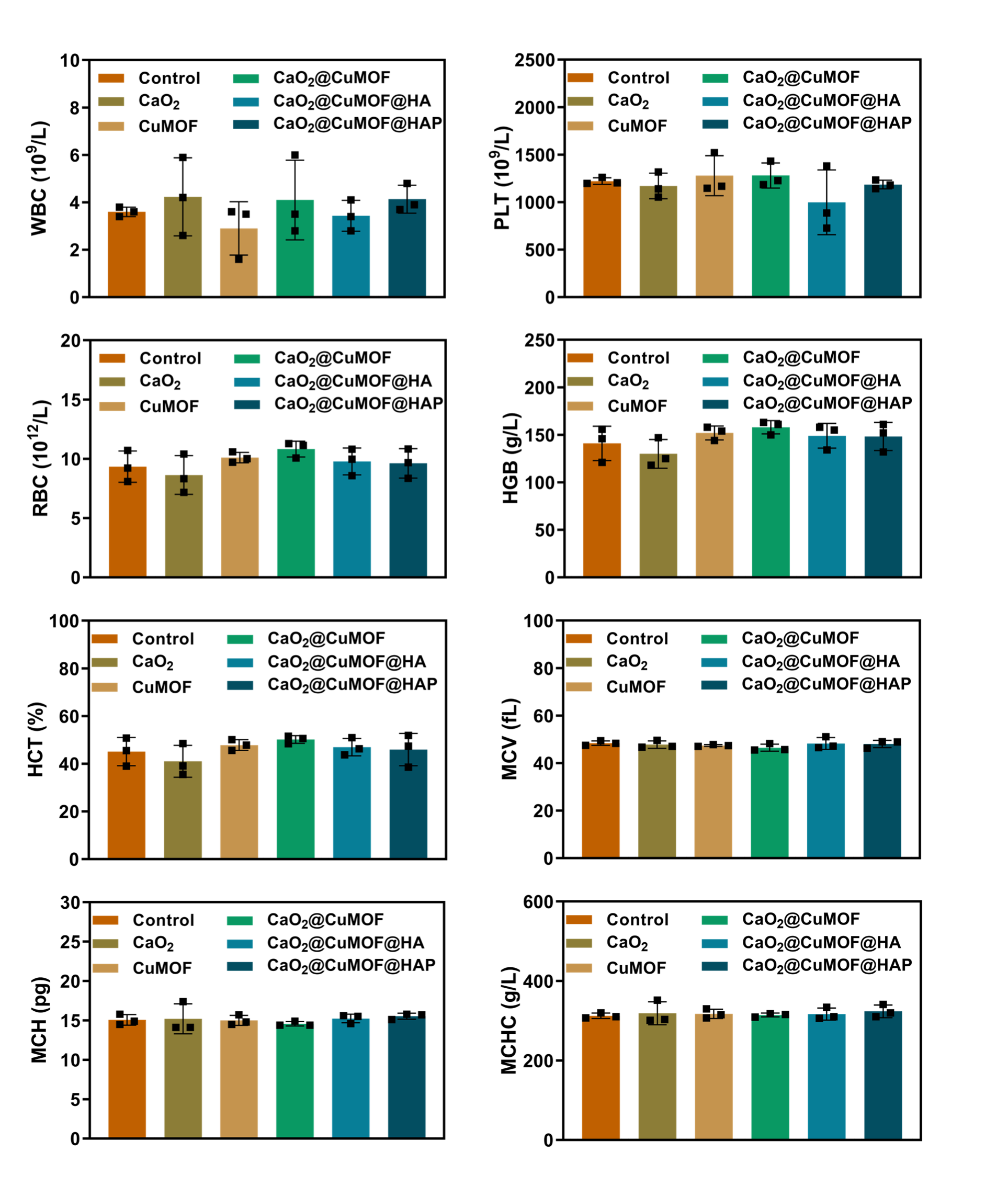
**

**Figure S25.** Complete blood count of mice with orthotopic OSCC after different treatments. Data were performed as the mean ± SD (n = 3 biologically independent samples). One-way ANOVA with Tukey’s post-hoc test was used for multiple comparisons.


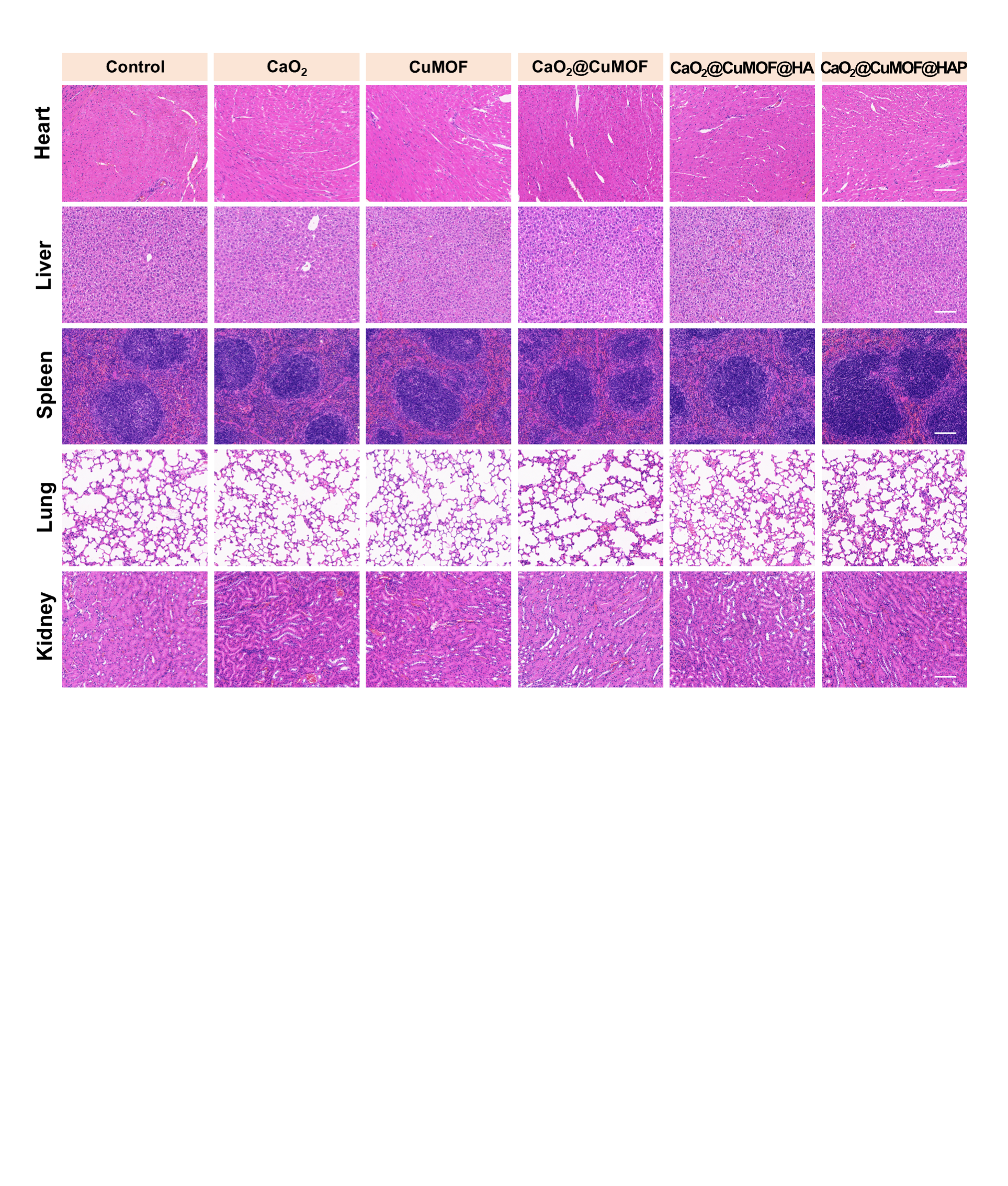


**Figure S26.** H&E staining images of major organs in mice bearing orthotopic oral squamous cell carcinoma (OSCC) after different treatments. Scale bar: 100 μm.

**
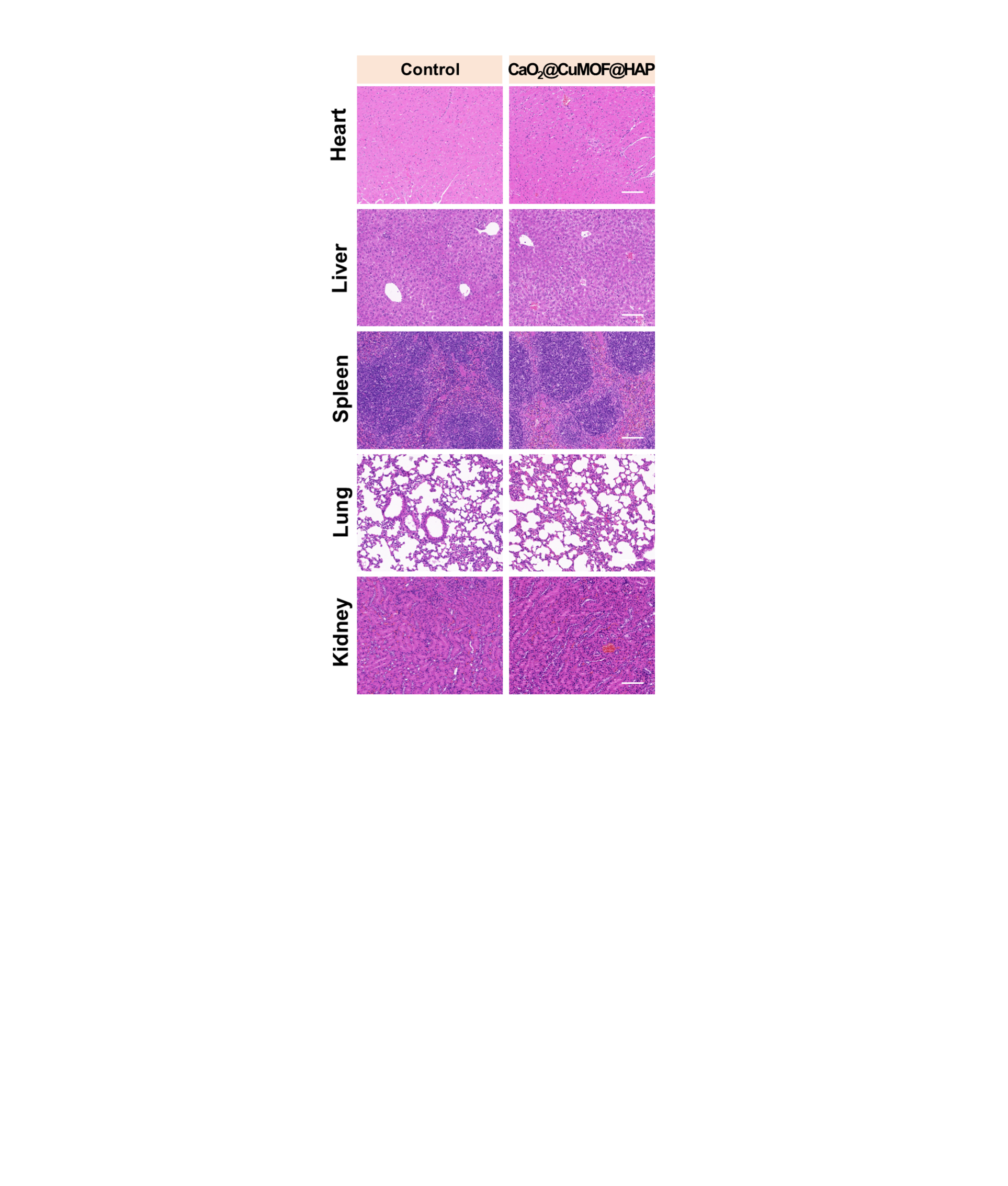
**

**Figure S27.** H&E staining images of major organs in mice after different treatments for 28 days. Scale bar: 100 μm.

**Figure S28.** MS analysis of the commercially synthesized GPLGLPGYGFGG.

**Figure S29.** MS analysis of the commercially synthesized GPLGLPGK(FITC)YGFGG.

Structure :GPLGLPGYGFGG

Lot NO :P240530-LR1172422

Number :0200049

Column :250*4.6mm,Kromasil-C18-5um

Solvent A:0.1%TFA in 100%water

Solvent B:0.1%TFA in 100%acetonitrile

Gradient : A B

0.1min 73% 27%

25.0min 48% 52%

25.1min 0% 100%

30.0min stop

Flow rate:1.0ml/min

Wavelength(nm):220

Volume :10ul

────────────────────────────

Rank Time Conc. Area Height

────────────────────────────

1 11.207 0.3576 14920.055 1569.720

2 11.703 0.6027 25145.834 2097.476

3 12.215 4.4200 184403.250 35479.992

4 12.358 92.2807 3849958.750 405429.59

5 12.358 2.1670 90408.289 7028.580

6 13.577 0.0759 3165.502 464.296

7 19.828 0.0960 4006.097 285.978

────────────────────────────

Total 100.00

**Figure S30.** HPLC analysis of the commercially synthesized GPLGLPGYGFGG.

Structure :GPLGLPGK(FITC)YGFGG

Lot NO :P240530-LR1172425

Number :0200049

Column :4.6×250mm,ChromCore 120 C18 5u

Solvent A:0.1%TFA in 100%water

Solvent B:0.1%TFA in 100%acetonitrile

Gradient : A B

0.1min 63% 37%

25.0min 38% 62%

25.1min 0% 100%

30.0min stop

Flow rate:1.0ml/min

Wavelength(nm):220

Volume :10ul

────────────────────────────

Rank Time Conc. Area Height

────────────────────────────

1 7.335 2.201 185189 26346

2 9.158 0.3964 33354 5773

3 9.378 94.02 7911002 740750

4 9.654 2.822 237421 30970

5 16.640 0.5601 47131 7349

────────────────────────────

Total 100 8414097 811188

**Figure S31.** HPLC analysis of the commercially synthesized GPLGLPGK(FITC)YGFGG.

**Table S1.** Primer sequences used for qRT-PCR.

| Species | Target gene | Forward sequence (5’-3’) | Reverse sequence (5’-3’) |
| --- | --- | --- | --- |
| Mouse | *Runx2* | GGGAACCAAGAAGGCACAGA | GGATGAGGAATGCGCCCTAA |
| Mouse | *Osx* | AGTGGGAACAAGAGTGAGCTG | TAGTGAGCTTCTTCCTGGGT |
| Mouse | *Col1a1* | GCCCAGACCTAGCAGACAC | TGGGCTTGGCATCTGTGAG |
| Mouse | *BSP* | TGCCGAAAGGAAGGTTAAGAAGA | TCGAGAAAGCACAGGCCATT |
| Mouse | *OCN* | GCCCAGACCTAGCAGACAC | TGGGCTTGGCATCTGTGAG |
| Mouse | *OPN* | CCTGGCTGAATTCTGAGGGAC | GCAGGCTGTAAAGCTTCTTCTC |
| Mouse | *β-actin* | GGCTGTATTCCCCTCCATCG | CCAGTTGGTAACAATGCCATGT |
